# The Human‐Imprinted Regional Species Pool: Functional Trait Patterns of Commercial and Established Plants

**DOI:** 10.1002/ece3.74610

**Published:** 2026-11-01

**Authors:** Vivien Grothe, Gala Mona Louise Dädlow, Joan Casanelles‐Abella, Sebastian Ruile, Marco Moretti, Bertrand Fournier

**Affiliations:** ^1^ Institute of Environmental Science and Geography University of Potsdam Potsdam Germany; ^2^ Urban Productive Ecosystems Technical University of Munich Freising Germany; ^3^ Biodiversity and Conservation Biology Swiss Federal Research Institute WSL Birmensdorf Switzerland; ^4^ Department of Environmental Systems Science ETH Zürich Zürich Switzerland

**Keywords:** commercial plant trade, community assembly, functional diversity, functional traits, human facilitation, species pools, urban ecology, urban green spaces

## Abstract

Urban plant communities assemble from a regional species pool shaped by both ecological processes and horticultural trade, yet how these two forces jointly structure its functional composition remains poorly understood. We characterized the potential regional species pool of Switzerland by compiling 5295 vascular plant species spanning herbs, shrubs, and trees and comparing established species (wild‐established only), commercial species (horticulturally available only), and overlapping species (both). Using a trait database covering life‐history strategies, morphology, environmental preferences, and human use, we examined functional trait space using principal component analysis and quantified functional richness, dispersion, and originality. We further tested which traits were associated with wild establishment among non‐native species and invasive status among overlapping species using Firth logistic regression. Commercial species were unevenly distributed across growth forms, dominating among trees (67.9%) and shrubs (59.7%) but less represented among herbs (17.9%). Functional differentiation was strongest along an environmental niche axis, with commercial herbs associated with drier conditions and commercial woody species with warmer conditions. Commercial species consistently had the lowest functional richness, dispersion, and originality across all growth forms. Wild establishment was associated with multiple environmental, reproductive, and ecological traits, whereas invasive status was associated with warmer temperature preference and anthropogenic tolerance, although the latter analysis was exploratory. These results indicate that commercial availability creates a functionally selective pool of species that may influence later urban community assembly. By shaping the functional possibilities available before local assembly occurs, horticultural trade may therefore leave an important human imprint on urban biodiversity, with potential downstream consequences for ecosystem functioning and trophic interactions.

## Graphical abstract

Urban plant communities assemble from a regional species pool shaped by both ecological processes and horticultural trade, yet how these forces structure its functional composition remains poorly understood. Using a comprehensive trait dataset across herbs, shrubs, and trees, we show that commercially available species occupy narrower and more clustered regions of trait space than established species, indicating strong functional convergence. This suggests that horticultural trade pre‐filters the functional potential of urban ecosystems before community assembly begins.

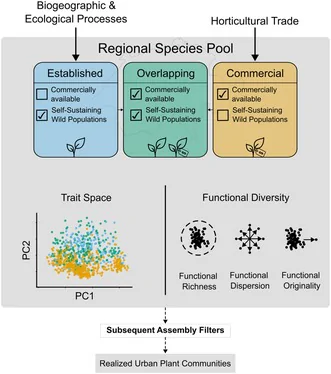

## 1 Introduction

Urbanization profoundly transforms landscapes, creating novel environments where plant communities are shaped under the combined influence of natural and anthropogenic factors (Aronson et al. 2016). Within cities, plant biodiversity contributes to ecosystem functions and services by regulating microclimates and nutrient cycling, providing habitat for diverse taxa, supporting biotic interactions such as pollination and herbivory, and enhancing human well‐being (Richards et al. 2022; Weiskopf et al. 2024). Unlike in natural ecosystems, however, urban plant community assembly is strongly influenced by human choices and management. These influences operate not only at the local scale by determining which species are planted or maintained but also at broader scales by shaping which species are available to enter urban communities in the first place.

A central concept for understanding community assembly is the regional species pool: the set of species available for local assembly within a geographic region (Zobel 2016). In ecological theory, species enter this pool through biogeographic and evolutionary processes (Cornell and Harrison 2014). In urban contexts, however, species can also become regionally available through human‐mediated pathways, particularly horticultural trade, regardless of their geographic origin (Aronson et al. 2016; Avolio et al. 2021; Cavender‐Bares et al. 2020). Local urban plant communities subsequently assemble from this pool through dispersal, environmental, and human‐related filters (Aronson et al. 2016; Cornell and Harrison 2014). Yet while considerable attention has been given to these local filters, far less is known about the functional structure of the regional pool from which urban communities assemble and how horticultural availability contributes to that structure.

Building on the conceptual distinctions introduced by Avolio et al. (2021), we distinguish two broad groups of plant species that together comprise what we term the *potential regional species pool for Swiss urban environments*, hereby simply referred to as the “regional species pool”. The first group, established species—analogous to “naturally dispersed” species in Avolio et al. (2021)—occur in the wild in Switzerland and maintain self‐sustaining populations without human intervention. This includes native species, whose presence in the regional pool reflects long‐standing biogeographical and eco‐evolutionary processes, as well as naturalized non‐native species, which arrived through past human‐mediated introductions but have since formed self‐sustaining populations (Cornell and Harrison 2014; Jiménez‐Alfaro et al. 2018). The second group, commercial species—corresponding to the subset of “human‐dispersed” species defined by Avolio et al. (2021)– enter the pool exclusively through horticultural trade: they are intentionally selected and planted, and do not maintain self‐sustaining wild populations (Avolio et al. 2021; Cavender‐Bares et al. 2020; Reichard and White 2001). Their presence in the pool reflects market incentives shaped by esthetic, cultural, and utilitarian preferences (Ellis et al. 2012; Goodness 2018; Van Kleunen et al. 2018; Williams et al. 2009). Importantly, these two groups are not mutually exclusive: species that are both established and commercially available are here termed *overlapping species*. Together, these three groups constitute our regional species pool: the set of species available for urban plant community assembly prior to local environmental and management filtering.

Because plant functional traits determine how species acquire resources, tolerate stress, and interact with their environment (Lavorel and Garnier 2002), the composition of this pool defines the range of ecological strategies available for urban communities, shaping the potential ecosystem functions and services they can provide (Díaz et al. 2007; Reich 2014). Horticultural trade may alter this composition by preferentially supplying species with particular trait combinations, for example those associated with ornamental value, ease of cultivation, or tolerance of stressful urban conditions (Ellis et al. 2012; Goodness 2018; Williams et al. 2009). Indeed, commercial availability can represent a non‐random subset of regional plant diversity: among native species in the Midwestern United States, commercially available species differed systematically from the wider regional flora in ecological characteristics, phenology, and phylogenetic representation (Zinnen et al. 2025). Depending on their position in trait space, commercial species could therefore either reinforce functional strategies already represented in the established flora or expand the regional pool by introducing underrepresented trait combinations. This can have important implications for competition, temporal resource dynamics (e.g., flowering phenology), interactions with organisms from other trophic levels, and the potential for future naturalization or invasiveness (Erickson et al. 2022; Lerch et al. 2024; Moreira and Abdala‐Roberts 2023). However, functional differences between commercial and established species need not reflect horticultural availability alone. Among non‐native species, they may also reflect which species are able to establish and persist outside cultivation. Identifying traits associated with wild establishment can therefore help distinguish these processes. Moreover, because horticultural trade is an important pathway for plant introductions (Hulme et al. 2018), understanding whether particular traits are also associated with invasive status is important for evaluating the potential ecological implications of the commercial pool.

Here, we examine the functional structure of the potential regional species plant pool for Swiss urban environments, integrating both commercially available and ecologically established species (Figure 1). Because herbs, shrubs, and trees differ in life‐history strategies (Díaz et al. 2016) and in the ways humans select and manage them in cities (Avolio et al. 2018; Gao et al. 2021), we analyze these growth forms separately. We first ask whether commercial, established, and overlapping species differ systematically in functional trait space and functional diversity. We then examine which traits are associated with wild establishment among non‐native species and with invasive status among overlapping species, extending our analysis from the functional structure of the regional pool to the processes potentially associated with persistence and spread beyond cultivation.

**FIGURE 1:**
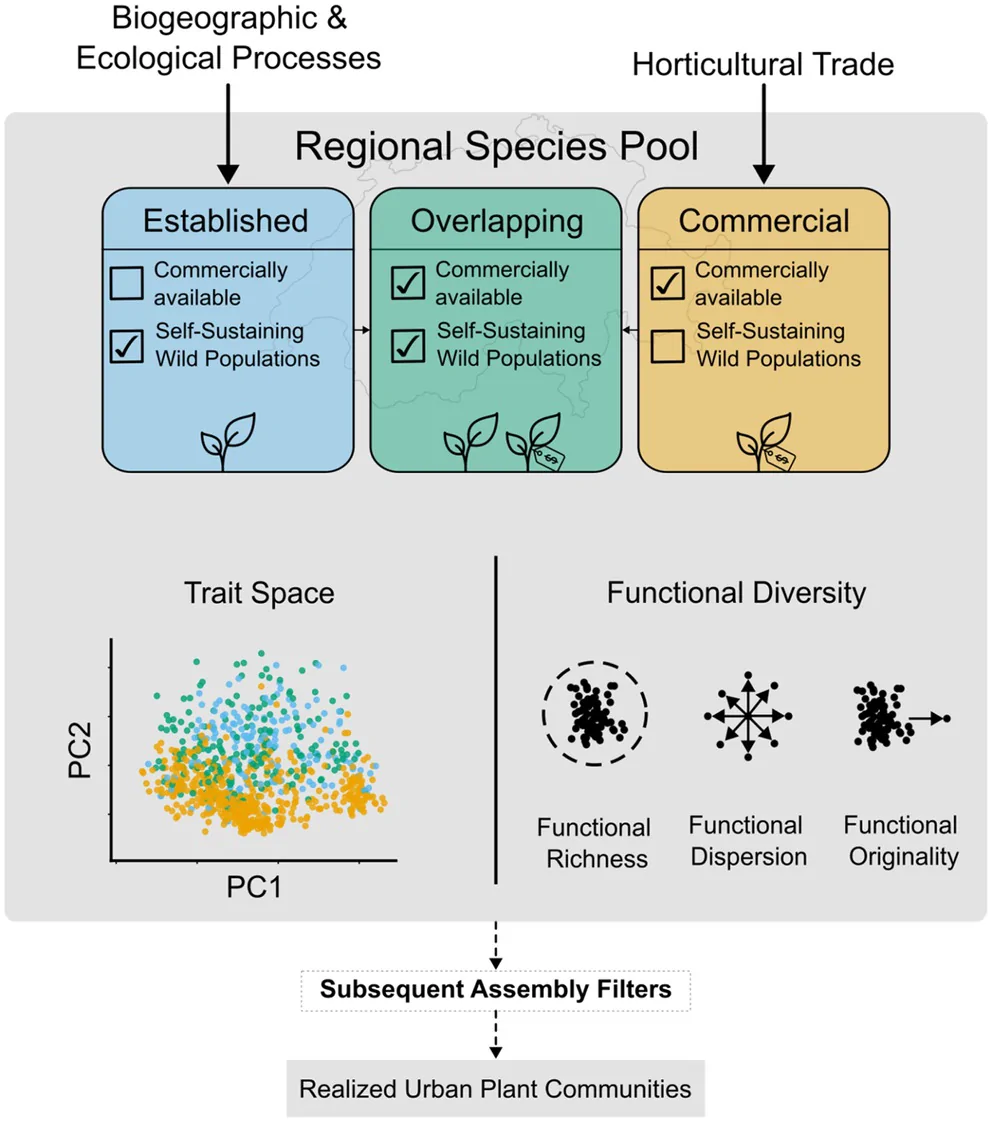
Conceptual representation of the potential regional species pool for Swiss urban environments. The regional species pool comprises established species, which maintain self‐sustaining wild populations but are not commercially available; commercial species, which are commercially available but do not maintain self‐sustaining wild populations; and overlapping species, which are both established and commercially available. These groups represent different contributions of ecological establishment and horticultural availability to the pool and are compared in this study in terms of their functional trait space and functional diversity. Local environmental, dispersal, management, and other human‐related filters subsequently act on this potential pool to shape realized urban plant communities; this subsequent stage of community assembly lies beyond the scope of the present study.

We expected commercial species to occupy a narrower and more clustered region of functional trait space than established species, reflecting the selective nature of horticultural trade. Because commercial availability is shaped by human preferences and market demands, we expected these species to converge around functional strategies associated with ornamental appeal, ease of cultivation and maintenance, and environmental tolerance. In contrast, we expected established species to span a broader range of functional strategies, as they comprise the full diversity of species capable of persisting in the wild in Switzerland. We further expected overlapping species to occupy an intermediate position, combining characteristics of both ecologically established and commercially selected species. Finally, because shrubs and trees are more strongly influenced by intentional planting than herbaceous species, we expected functional differences among species groups to be more pronounced for woody growth forms. To test these expectations, we addressed four questions:

- How are commercial, established, and overlapping plant species represented across growth forms (herbs, shrubs, trees) within the potential regional species pool for Swiss urban environments?
- How do commercial, established, and overlapping plant species differ in their occupation of multidimensional functional trait space, and do differences vary among growth forms?
- How do these three plant species groups differ in functional diversity metrics, and what does this reveal about how commercial availability constrains or expands functional strategies available to urban plant communities?
- Which functional traits are associated with establishment among non‐native species, and which traits are associated with invasive status among overlapping species?

By linking horticultural availability, functional trait structure, and ecological establishment, this study treats the regional species pool as an intermediate stage through which human selection can influence subsequent urban community assembly. This framework provides a basis for understanding not only which functional strategies are made available to urban plantings but also whether the same strategies may be associated with persistence and spread beyond cultivation. This understanding represents an essential first step toward managing urban biodiversity in increasingly human‐dominated landscapes.

## 2 Materials and Methods

### 2.1 Study Area

Switzerland was chosen as the study system. The country spans pronounced climatic and biogeographic gradients, from collinean lowlands to alpine zones, and is undergoing rapid urbanization, with more than three‐quarters of the population living in cities (FDFA 2023) and settlement areas having expanded by over a third since 1985 (Federal Office for the Environment FOEN 2022).

### 2.2 Defining the Regional Species Pool

We define the regional species pool as all plant species that could potentially occur in Swiss urban environments, comprising three groups: established, commercial, and overlapping species. Established species are those naturally occurring in Switzerland that propagate without human intervention, including native species and naturalized non‐natives with self‐sustaining wild populations (Avolio et al. 2021). Commercial species are available for purchase in Switzerland but have not formed self‐sustaining wild populations; they enter cities exclusively through intentional planting. Overlapping species are both naturally established and commercially available. Together, these three groups constitute the regional species pool in our analyses. Hereafter, we refer to these collectively as “groups,” and any comparisons between groups refer specifically to comparisons between commercial, established, and overlapping species.

We define establishment at the national rather than urban scale because our regional pool represents species potentially available for local urban assembly, rather than species already occurring within particular urban communities. Restricting this pool to species currently recorded in cities could exclude species with genuine colonization potential, as dispersal and establishment depend on spatially variable factors such as proximity, propagule pressure, and local environmental conditions that cannot be reliably predicted a priori. Such a definition would also make the regional pool dependent on the particular cities and habitats sampled. We therefore use the national established flora as the starting point for representing species with potential to enter Swiss urban communities, while recognizing that subsequent dispersal, environmental, and management filters determine which species occur locally. We additionally used an independent survey of urban green spaces to assess and complement the coverage of this national‐scale species pool (see Section 2.3).

### 2.3 Species Data Collection

Established species were obtained from the national data and information centre of the Swiss flora (Info Flora 2023b; https://www.infoflora.ch/en/; downloaded 23.06.2023), which integrates occurrence records collected within the Swiss Biodiversity Monitoring (BDM) programme. The BDM only records plant species that maintain self‐sustaining, persistent populations over at least the past decade and function as integral components of native vegetation in both natural and human‐modified habitats. We filtered the dataset to include only spermatophyte taxa (excluding fully aquatic plant species) and restricted the time period to 2013–2022, resulting in a final list of 3648 established species (5127 plots with a mean of 5.8 taxa per plot).

Commercial species were primarily sourced from InfoFlora's Green List, a list that provides planting recommendations for private households and indicates whether species are horticulturally available in Switzerland (Info Flora Green List 2023a; https://www.infoflora.ch/en/; downloaded 23.06.2023). This was complemented by a species list provided by JardinSuisse, the national association representing Swiss horticultural enterprises, which includes species sold by major Swiss plant vendors and nurseries (https://www.jardinsuisse.ch/de/home/; pers. comm.; received 17.11.2023). Together, these sources capture both private and professional horticultural availability within Switzerland. A total of 2736 plant species commonly sold in Switzerland were identified. While additional species can be ordered online from international retailers, including these would substantially expand the species pool beyond what is commonly planted in Switzerland and thus beyond the scope of this study.

To complement and assess the coverage of our regional species pool, we incorporated plant occurrence data from a field sampling campaign conducted in July 2023 as part of the PAPPUS project (Casanelles‐Abella et al. 2026). Plant occurrences were recorded in 180 urban green spaces across Zurich, Geneva, and Lugano using a random stratified sampling design along an urbanization gradient and spanning five habitat types: allotments, private gardens, parks, ruderal areas, and real estate gardens. Details of the sampling design are provided in Casanelles‐Abella et al. (2026). The survey identified 383 additional species that were not represented in the established or commercial source lists described above. Because these species were absent from Info Flora and therefore not recorded as having self‐sustaining populations in Switzerland, their occurrence within the sampled urban green spaces was interpreted as resulting from intentional buying and planting, and they were assigned to the commercial group.

All plant taxa were standardized against the World Flora Online taxonomic backbone (WFO: World Flora Online 2022; downloaded 21.12.2023) using the *WorldFlora* R package (Kindt 2020). Accepted WFO names were preferentially retained, while synonyms listed in the WFO backbone were stored as alternative names and used where necessary for matching taxa to trait databases. Subspecies, varieties, and hybrids were retained where recognized by WFO. Cultivars, however, were not treated as separate taxonomic units but were reduced to their underlying species. This was necessary to maintain a consistent species‐level framework across data sources, as cultivar‐specific trait information was not available from the trait databases used in this study. Ambiguous taxonomic matches were manually inspected and resolved where possible; taxa for which a reliable taxonomic assignment could not be made were excluded.

### 2.4 Native/Non‐Native Classification

Native/non‐native status was derived from the Info Flora Checklist 2017 (Juillerat et al. 2017; downloaded 20.07.2026), the official taxonomic reference database for vascular plants in Switzerland. Taxon names in the checklist were harmonized against the World Flora Online taxonomic backbone (WFO; downloaded 21.12.2023). Taxa classified by Info Flora as indigenous, archaeophytes (present in Switzerland before 1500), or neo‐indigenous (presumed to have colonized Switzerland after 1500 without human assistance) were considered native. Neophytes (appearing in Switzerland after 1500), as well as cultivated archaeophytes (cultivated plants present before 1500 that cannot maintain populations without human assistance) and cultivated neophytes (cultivated plants introduced after 1500 with no tendency to naturalize), were considered non‐native. Commercial species that were not present in the Info Flora Checklist were classified as non‐native. Taxa with ambiguous classifications or for which no origin status was available were excluded from analyses requiring native/non‐native status. This resulted in the exclusion of 117 herb and 13 shrub species; no tree species were excluded.

### 2.5 Trait Data Collection

The selected plant traits capture key aspects of species' life histories, phenology, morphology, and physiology. In addition to widely used plant traits such as specific leaf area (SLA), plant height, and seed mass, we also included traits describing environmental preferences (e.g., Landolt indicator values (Landolt et al. 2010)), ecological strategy (e.g., CSR categories (Grime 1977)) and human‐disturbance tolerance. In addition, to better reflect the processes shaping urban plant communities, we included traits related to flower morphology, toxicity, and human uses (e.g., food, medicinal, or ornamental uses), which may influence species selection and management in cities. Together, this trait set captures both ecological and human‐mediated dimensions of plant performance in cities. A full list of traits used, including their sources and short descriptions, is provided in the Supporting Information (Appendix S1).

Trait data primarily originated from the TRY Plant Trait Database (Kattge et al. 2020; downloaded 04.12.2023; Appendix S7). Records of juvenile plants, saplings, and individuals grown under experimental conditions were excluded. For numerical traits, we followed the outlier detection approach described by the *rtry* R package vignette (Lam et al. 2024). Specifically, we removed trait records with values > 3 standard deviations from the species mean. For categorical traits, we assigned each species its most frequently reported value. We complemented our TRY data with the Botanical Information Ecology Network (BIEN) (Enquist et al. 2016; Maitner et al. 2018; version 4.2.8), iGarten (Brüngger, Felix; www.igarten.ch; version 7.0.0; pers. comm.; downloaded 01.03.2024), the Toxic Plants‐Phytotoxin database (TPPT) (Günthardt et al. 2018), Flora Indicativa (Landolt et al. 2010) and Botanica (Burnie et al. 2003). Finally, the 10 most extreme values of each trait were manually checked for reliability.

Prior to imputation, trait coverage averaged 62%, but varied substantially among traits. Some traits showed near‐complete coverage (e.g., flower color and leaf compoundness, up to 99%), whereas others were considerably less complete (e.g., life span, 43%). To address missing values, we evaluated five imputation methods: mean imputation, predictive mean matching, classification and regression trees, random forest implemented in the *mice* package (Buuren & Groothuis‐Oudshoorn, 2011), and missForest, another imputation method based on random forest implemented in the missForest package (Stekhoven and Bühlmann 2012). Imputation performance was assessed by artificially introducing missing values into the dataset and comparing imputed values to the original observed values. As an initial screening step, we compared the distributions of original and imputed data and excluded methods that substantially altered trait distributions. We then quantified imputation performance by comparing the imputed datasets to the original dataset (prior to the introduction of artificial missing values). For continuous traits, performance was evaluated using the root mean squared error (RMSE) and *R* ^2^, with lower RMSE and higher *R* ^2^ indicating better performance. For categorical traits, we used Cohen's *κ* and overall accuracy, giving priority to *κ* (with accuracy used as a secondary criterion when *κ* values were similar). Across all traits, missForest consistently performed best (average *κ* = 0.425; and *R* ^2^ = 0.375). Full results of the imputation evaluation are provided in the Supporting Information (Appendix S2).

Following imputation, categorical traits were converted to numerical variables. Ordered traits retained their ordinal scores, whereas unordered traits were one‐hot encoded, with each category represented by a separate binary (0/1) column. When preparing the data for PCA, one category/column was omitted from each mutually exclusive categorical trait to avoid including a redundant binary variable. Multilabel traits, such as CSR strategy, retained one binary column for each possible category because species could belong to several categories simultaneously.

### 2.6 Analysis of Functional Trait Space

We examined the functional trait space of the regional species pool using Principal Component Analysis (PCA). Separate PCAs were conducted for herbs, shrubs, and trees to allow for a more detailed examination of trait variation within each growth form. PCAs were performed using the *prcomp* function from the *stats* package. Before conducting the PCAs, traits showing zero variance were removed and all remaining traits were standardized.

To test whether commercial, established, and overlapping species occupied different positions in trait space, we conducted Kruskal–Wallis tests on species' coordinates along the first principal component axis (PC1) and the second principal component axis (PC2) using the kruskal.test function from the *stats* package. To assess whether group differences also occurred in dimensions not represented by PC1 and PC2, we additionally performed Kruskal–Wallis tests on all remaining PCA axes retained for the functional‐space analyses (see next section). For each axis, we calculated rank‐based eta‐squared (*η* ^2^ *H*) effect sizes with 95% confidence intervals using the *kruskal_effsize* function from the *rstatix* package (Kassambara 2023). For PC1 and PC2, significant overall differences were followed by pairwise Dunn's tests with Holm correction, implemented using the *dunn_test* function from *rstatix* to identify which groups differed significantly.

### 2.7 Quantifying Functional Trait Space With Diversity Indices

To complement our PCA analysis, we quantified how commercial, established, and overlapping species occupy functional trait space using three α‐diversity indices: functional richness (FRic), defined as the volume of the convex hull occupied by species in multidimensional trait space; functional dispersion (FDis), defined as the mean distance of species to the centroid of the assemblage; and functional originality (FOri), defined as the mean distance between each species and its nearest neighbor across all species in the trait space. For each growth form, we calculated pairwise functional distances among all species using Gower distance and constructed a multidimensional trait space via Principal Coordinates Analysis (PCoA), selecting the number of axes that minimized the deviation between original Gower distances and their Euclidean representation in the reduced space (Maire et al. 2015). Commercial, established, and overlapping species were then defined as separate assemblages within this shared trait space.

To account for unequal species group sizes, which can inflate FRic estimates for larger groups, we calculated all three indices using a rarefaction approach. For each growth form, we repeatedly subsampled each group to a standardized sample size (trees: *n* = 25; shrubs: *n* = 150; herbs: *n* = 600) without replacement across 1000 iterations. FRic was calculated as the scaled convex hull volume following the geometric definitions of Magneville et al. (2022); FDis and FOri were calculated using the *alpha.fd.multidim* function from the *mFD* package (Magneville et al. 2022). For each group and index, we then calculated the mean across the 1000 iterations, with the 2.5th and 97.5th percentiles used as 95% rarefaction intervals.

### 2.8 Wild Establishment and Invasive Status

To examine whether functional differences between commercial and established species reflect horticultural selection alone, or may also arise because species with certain traits are simply more likely to establish in the wild, we tested trait associations with wild establishment among non‐native species. We focused on non‐native species because their occurrence in the established flora reflects successful establishment following introduction, allowing us to compare wild‐established species (established‐only and overlapping) with species that remain commercial‐only.

In a separate analysis addressing a different question, we examined which functional traits were associated with invasive status among overlapping species. We focused on the overlapping group because these species are both commercially available and established in the wild, making their invasive status particularly relevant when considering their potential use in urban planting. Invasive status was defined independently of our commercial and establishment classifications based on whether a species was listed as invasive in Switzerland (InfoSpecies 2021).

Both analyses used Firth's penalized logistic regression implemented in the *logistf* package (Heinze et al. 2025). Growth forms were pooled because sample sizes, particularly for trees and invasive species, were insufficient for separate analyses, and growth form was therefore included as a covariate in all models. The same a priori set of functional traits was evaluated in both analyses. Candidate traits were screened for near‐zero variance and pairwise collinearity (Pearson's |*r*| > 0.7); no traits were excluded based on these criteria. Each trait was then evaluated separately in a Firth logistic regression model that included growth form as a covariate. To account for multiple testing, *p*‐values were adjusted across trait models using the Benjamini‐Hochberg procedure. Associations are reported as odds ratios (ORs) with 95% confidence intervals. Because the invasive‐status analysis included only 25 invasive species, these results were considered exploratory.

All data preparation and analyses were conducted in R version 4.4.3 (R Core Team 2025).

## 3 Results

### 3.1 Composition of the Regional Species Pool

The regional species pool comprised a total of 5295 plant species, with a predominance of herbs (3879 species, 73.3%), followed by shrubs (921 species, 17.4%) and trees (495 species, 9.3%) (Figure 2). Of all species in the pool, 48.3% were classified as established, 29.9% as commercial, and 21.8% as overlapping (i.e., species both sold commercially and found established in the wild). The relative importance of these groups varied markedly across growth forms. Trees showed the highest proportion of commercial species (67.9%), followed by shrubs (59.7%) and herbs (17.9%). Conversely, herbs exhibited the highest proportion of established species (60.9%), while trees showed the lowest (6.6%).

**FIGURE 2:**
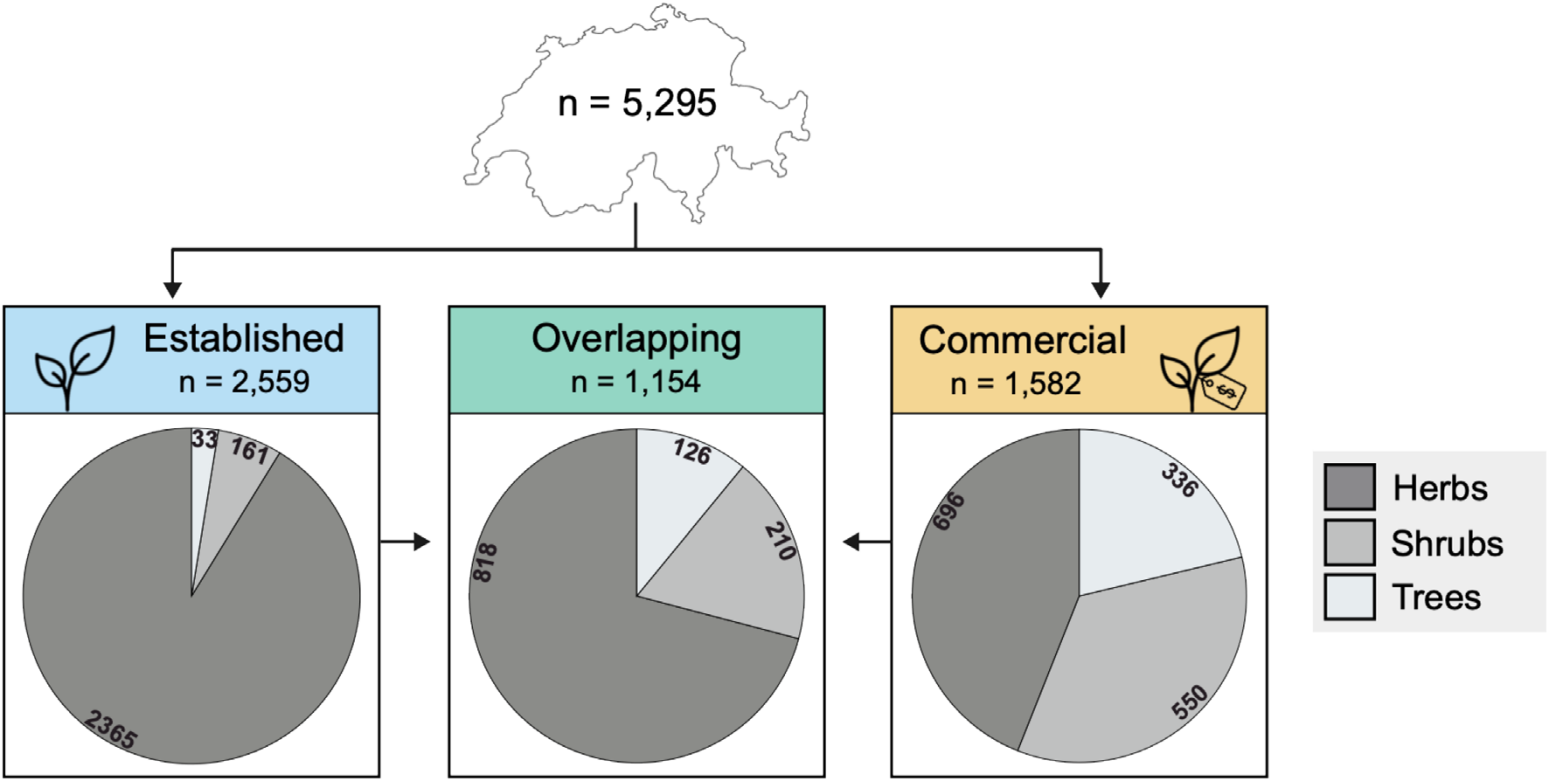
Composition of the regional species pool, comprising established (self‐sustaining in the wild), commercial (available commercially), and overlapping species (both established and commercially available). Total species counts (*n*) are shown for each group and growth form. Most herbs were classified as established (60.9%), whereas most shrubs (59.7%) and trees (67.9%) were classified as commercial.

### 3.2 Functional Trait Space Patterns

We used PCAs to explore how commercial, established, and overlapping species occupy the functional trait space of our regional species pool. The percentage of variance explained by each axis and trait loadings are provided in the Supporting Information (Appendix S3).

#### 3.2.1 Herbs

PC1 and PC2 together explained 22% of the total trait variation among herbs (Figure 3). Kruskal–Wallis tests revealed significant differences in species coordinates between the three groups along both PC1 (*χ* ^2^ = 75.8, df = 2, *p* < 0.001, *η* ^2^ *H* = 0.019, 95% CI = 0.01–0.03) and PC2 (*χ* ^2^ = 592.3, df = 2, *p* < 0.001, *η* ^2^ *H* = 0.152, 95% CI = 0.13–0.17). However, the effect was small along PC1 and large along PC2. Although significant group differences were also detected along the remaining PCA axes, PC2 was the only axis showing a large effect (Appendix S4, Table A).

**FIGURE 3:**
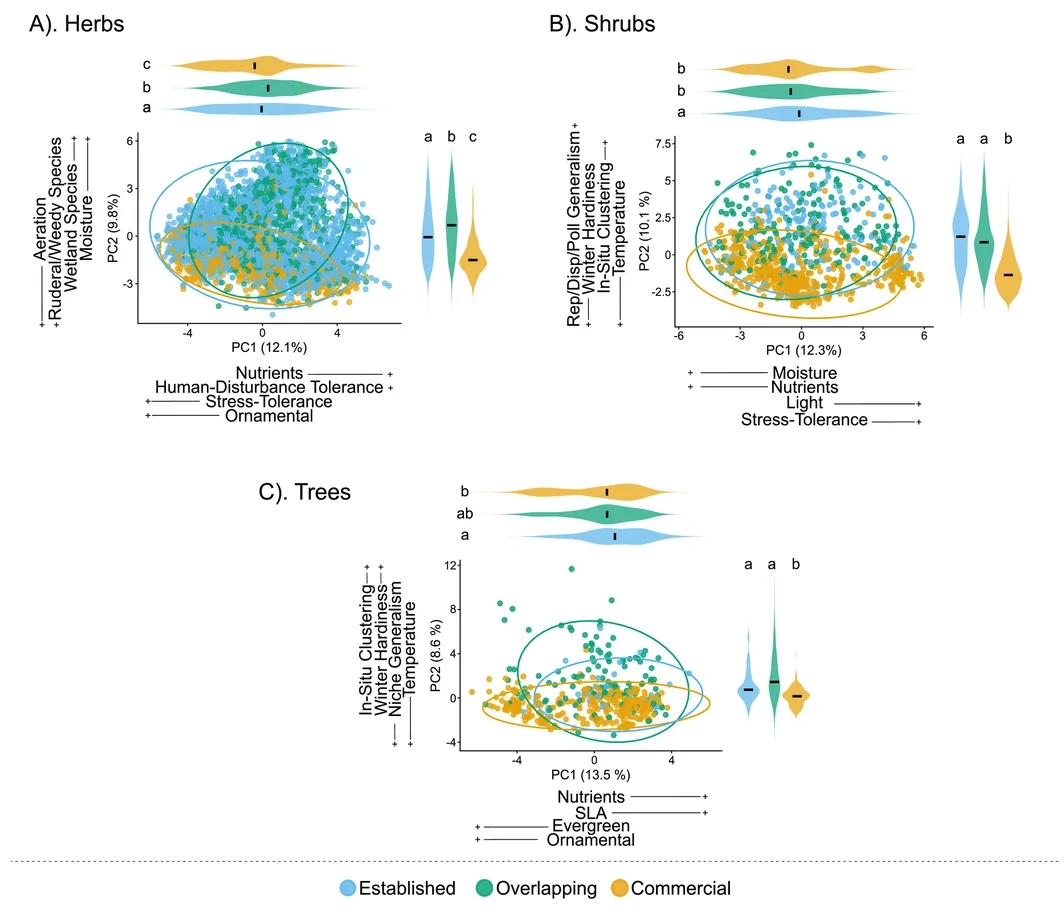
Functional trait space of the regional species pool. PCAs of species‐level trait data were conducted separately for (A) herbs (*n* = 3879, 48 traits), (B) shrubs (*n* = 921, 47 traits), and (C) trees (*n* = 495, 44 traits). Each point represents a single species, color‐coded by group (commercial, established, or overlapping). Trait labels below the axes show the four traits with the highest contributions to each axis, with the trait having the largest contribution placed nearest to each axis. Horizontal and vertical violin plots show the density distribution of commercial, established, and overlapping species along PC1 and PC2, respectively. Letters above the violins show results of post hoc Dunn's tests; groups sharing a letter do not differ significantly in their PC scores (*α* = 0.05).

PC1 separated disturbance‐tolerant herbs requiring high nutrient levels from stress‐tolerant species adapted to nutrient‐poor sites (Figure 3A). Pairwise Dunn's test revealed significant pairwise differences among groups, indicating that commercial, established, and overlapping species occupy distinct areas along this axis (Appendix S4, Table B). Trait means revealed that commercial herbs exhibited higher mean human‐disturbance tolerance than established and overlapping species (Appendix S8). In contrast, overlapping species exhibited higher average stress tolerance and nutrient demand than the other groups (Appendix S8).

PC2 distinguished wetland herbs adapted to moist environments from ruderal and weedy herbs suited to drier, more aerated soils. Commercial species clustered toward the drier, ruderal end of the axis, while established and overlapping species extended toward wetter conditions (Figure 3A). Pairwise Dunn's tests again showed significant pairwise differences among groups (Appendix S4, Table B), which is also supported by trait means: commercial herbs included fewer wetland species and more ruderal taxa, while the other groups contained a higher proportion of wetland, moisture‐adapted species (Appendix S8).

#### 3.2.2 Shrubs

PC1 and PC2 together explained 22% of the total trait variation among shrubs (Figure 3). Kruskal–Wallis tests revealed significant differences in species coordinates between the three groups along both PC1 (*χ* ^2^ = 9.56, df = 2, *p* = 0.008, *η* ^2^ *H* = 0.008, 95% CI = 0–0.03) and PC2 (*χ* ^2^ = 360.21, df = 2, *p* < 0.001, *η* ^2^ *H* = 0.39, 95% CI = 0.34–0.44). However, the effect was small along PC1 and large along PC2. Although significant group differences were also detected along some of the remaining PCA axes, PC2 was the only axis showing a large effect (Appendix S4, Table A).

PC1 reflected a gradient from low stress‐tolerant, high‐moisture, and nutrient‐demanding shrubs to stress‐tolerant shrubs from drier, nutrient‐poor habitats that are also more light‐demanding. Visual inspection suggested that there is no distinct group clustering along this axis. Indeed, pairwise Dunn's tests revealed no significant differences between commercial and overlapping species, while established species differed significantly from both other groups (Appendix S4, Table B). Trait means showed that established shrubs are generally more stress‐tolerant, whereas commercial shrubs are more tolerant of human disturbances (Appendix S8).

PC2 was driven by temperature tolerance and reproductive traits. It separated cold‐tolerant shrubs with high winter hardiness from shrubs associated with warmer habitats. This axis also captured variation in reproductive strategy and growth patterns, contrasting species that employ a broad range of pollination, dispersal, and reproductive strategies and tend to grow in clusters with those that are more sparsely distributed and rely on a narrower set of strategies. Visually, commercial species formed a distinct cluster in the lower end of PC2, associated with warmer conditions, more specialized reproductive strategies, and non‐clustered growth. Pairwise Dunn's tests provided further support for this pattern, with commercial species differing significantly from established and overlapping species along PC2, but established and overlapping species not differing significantly (Appendix S4, Table B). Trait means aligned with these results: commercial shrubs showed higher temperature requirements, narrower reproductive breadth, and less‐clustered growth than established or overlapping species, while the latter two groups displayed broadly similar trait means (Appendix S8).

#### 3.2.3 Trees

PC1 and PC2 together explained 22% of the total trait variation among trees (Figure 3). Kruskal–Wallis tests revealed significant differences in species coordinates between the three groups along both PC1 (*χ* ^2^ = 6.72, df = 2, *p* = 0.03, *η* ^2^ *H* = 0.01, 95% CI = −0.002–0.04) and PC2 (*χ* ^2^ = 116.6, df = 2, *p* < 0.001, *η* ^2^ *H* = 0.233, 95% CI = 0.16–0.31). However, the effect was small along PC1 and large along PC2. Although significant group differences were also detected along some of the remaining PCA axes, PC2 was the only axis showing a large effect (Appendix S4, Table A).

PC1 captured a trade‐off between deciduous, fast‐growing trees with high specific leaf area (SLA) and nutrient needs, versus evergreen, low‐SLA trees with higher ornamental value. Commercial species extended toward the latter end of this axis, suggesting a prevalence of “slow,” evergreen species with high ornamental purpose. Pairwise Dunn's tests revealed significant pairwise differences along PC1 only between established and commercial species (Appendix S4, Table B). Trait means support this pattern: commercial trees included, on average, a higher share of evergreen species, lower SLA values, lower nutrient requirements, and greater ornamental use than established species (Appendix S8).

PC2 contrasted dry‐adapted, warm‐temperature trees with low winter hardiness and low niche generalism against wetter‐adapted, cold‐tolerant trees with broader niche generalism. In situ clustering also contributed to this axis. Commercial species are clearly separated from established and overlapping species along PC2, clustering toward the warmer, drier end of the gradient and associated with narrower niche variation and non‐clustered growth. Pairwise Dunn's tests revealed that commercial species differed significantly from the other two groups, while established and overlapping species did not differ significantly (Appendix S4, Table B). Trait means reinforce this pattern: commercial trees are associated with warmer and drier habitats, lower winter hardiness, reduced niche generalism, and less‐clustered growth compared to established and overlapping species (Appendix S8).

### 3.3 Functional Diversity Metrics

#### 3.3.1 Herbs

Functional indices were calculated within a multidimensional trait space derived from PCoA, retaining 9 axes. Established herbs had the highest mean functional richness (FRic: 0.33), functional dispersion (FDis: 0.62), and functional originality (FOri: 0.26), indicating a broader trait space and greater internal spread than the other groups (Figure 4). Overlapping species showed intermediate values for functional richness (FRic: 0.22), dispersion (FDis: 0.56), and originality (FOri: 0.25). Commercial species had the lowest mean values for all three indices (FRic: 0.19, FDis: 0.55, FOri: 0.16), indicating a comparatively narrower and less functionally distinctive trait space. Mean values and 95% rarefaction intervals are provided in Appendix S5.

**FIGURE 4:**
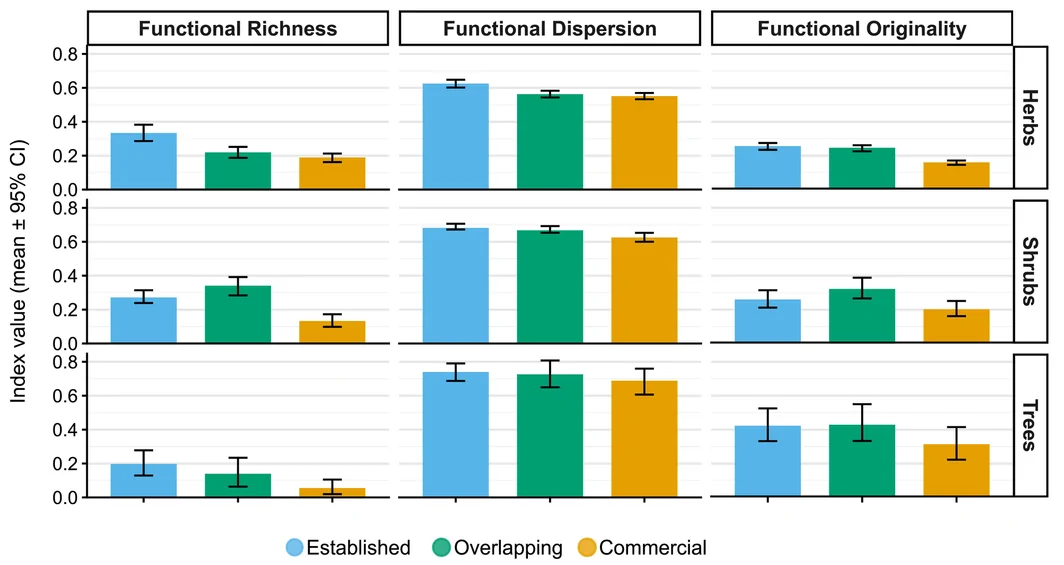
Mean functional richness, dispersion, and originality of established, overlapping, and commercial species across growth forms. Bar plots showing mean functional richness, functional dispersion, and functional originality for herbs, shrubs, and trees, based on multidimensional trait spaces derived from PCoA. To account for differences in species richness among groups, indices were calculated across 1000 rarefaction iterations using equal numbers of species per group within each growth form (herbs: *N* = 600; shrubs: *N* = 150; trees: *N* = 25). Bars represent mean values across rarefaction iterations and error bars show 95% rarefaction intervals.

#### 3.3.2 Shrubs

For shrubs, functional indices were calculated in a PCoA‐based trait space with 7 retained axes. Overlapping shrubs had the highest mean functional richness (FRic: 0.34) and functional originality (FOri: 0.32), followed by established shrubs (FRic: 0.27, FOri: 0.26), whereas commercial shrubs had the lowest values for both indices (FRic: 0.13, FOri: 0.20) (Figure 4). Differences in functional dispersion were comparatively small (established: FDis = 0.68; overlapping: FDis = 0.67; commercial: FDis = 0.63), with overlapping rarefaction intervals indicating limited differentiation among groups for this index. Mean values and 95% rarefaction intervals are provided in Appendix S5.

#### 3.3.3 Trees

Among trees, indices were calculated in a PCoA‐based trait space with 6 retained axes. Established trees had the highest mean functional richness (FRic: 0.20) and functional dispersion (FDis: 0.74), as well as relatively high functional originality (FOri: 0.42) (Figure 4). Overlapping species had lower functional richness (FRic: 0.14), similar dispersion (FDis: 0.73), and similar but higher mean originality (FOri: 0.43). Commercial species had the lowest mean values across all three indices (FRic: 0.06, FDis: 0.69, FOri: 0.31). However, rarefaction intervals were relatively wide and overlapped considerably among groups, particularly for FDis and FOri, indicating limited differentiation among groups for these indices. Mean values and 95% rarefaction intervals are provided in Appendix S5.

### 3.4 Trait Associations With Wild Establishment and Invasive Status

Functional traits were associated with wild establishment among non‐native species (Figure 5A). Sexual reproduction was positively associated with establishment, with species capable of sexual reproduction having 2.60 times the odds of being established in the wild rather than commercial‐only (OR = 2.60, 95% CI = 2.15–3.15, adjusted *p* < 0.001). Reproductive, pollination, and dispersal generalism was also positively associated with establishment (OR = 2.27, 95% CI = 2.04–2.52, adjusted *p* < 0.001), as were niche generalism (OR = 1.69, 95% CI = 1.51–1.91), moisture preference (OR = 1.43, 95% CI = 1.34–1.52), human‐disturbance tolerance (OR = 1.33, 95% CI = 1.20–1.48), and SLA (OR = 1.08, 95% CI = 1.07–1.09; all adjusted *p* < 0.001). In contrast, LDMC was negatively associated with establishment (OR = 0.005, 95% CI = 0.002–0.018, adjusted *p* < 0.001). Higher winter‐hardiness values, which indicate lower cold tolerance, were also associated with lower odds of establishment (OR = 0.96, 95% CI = 0.95–0.97, adjusted *p* < 0.001), meaning that more winter‐hardy species were more likely to be established. Temperature preference, light preference, seed mass, and plant height were not significantly associated with establishment (adjusted *p* > 0.05). Full model results are provided in Appendix S6.

**FIGURE 5:**
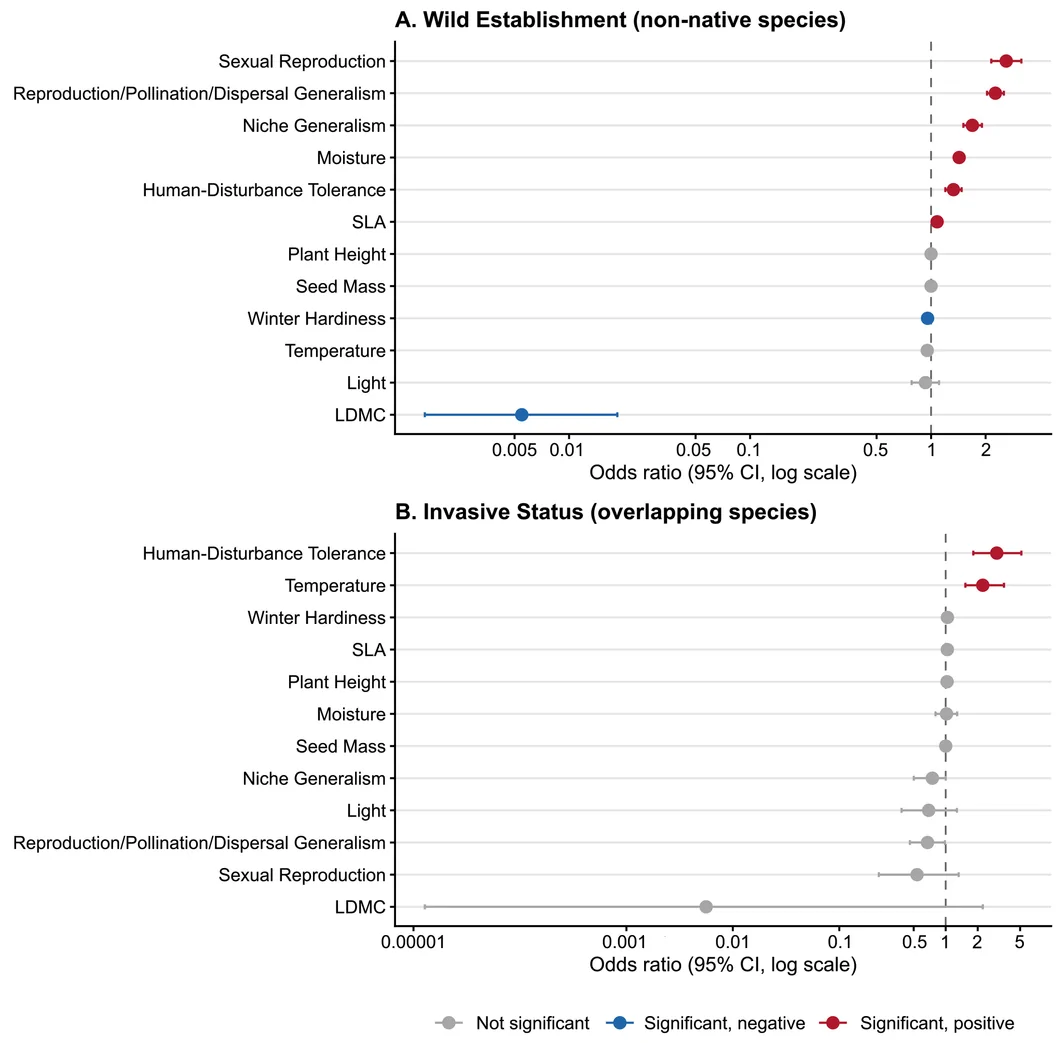
Associations of functional traits with wild establishment and invasive status. Odds ratios (ORs) and 95% confidence intervals from Firth penalized logistic regression models examining (A) wild establishment among non‐native species, comparing species established in the wild (established‐only and overlapping species) with commercial‐only species, and (B) invasive status among overlapping species. Each trait was analyzed in a separate model with growth form included as a covariate. Points represent odds ratios and horizontal lines their 95% confidence intervals; the dashed vertical line indicates no association (OR = 1). Red and blue indicate significant positive and negative associations, respectively (Benjamini–Hochberg‐adjusted *p* < 0.05), while gray indicates non‐significant associations. Note that odds ratios represent a one‐unit increase in each trait and therefore effect sizes should not be directly compared among traits measured on different scales. LDMC, leaf dry matter content; SLA, specific leaf area. Results for invasive status should be interpreted as exploratory because of the small number of invasive species (*n* = 25).

Among overlapping species, anthropogenic tolerance and temperature preference were positively associated with invasive status (Figure 5B). Higher anthropogenic tolerance was associated with greater odds of being invasive (OR = 3.02, 95% CI = 1.82–5.14), as was warmer temperature preference (OR = 2.23, 95% CI = 1.53–3.52). These were the only associations that remained significant after correction (adjusted *p* < 0.05). The remaining traits showed no significant associations with invasive status after correction (adjusted *p* > 0.05). Given the small number of invasive species (*n* = 25), associations with invasive status should be interpreted as exploratory. Full model results are provided in Appendix S6.

## 4 Discussion

This study characterizes the functional structure of the potential regional species pool for Swiss urban environments, comprising commercial species (horticulturally available, not wild‐established), established species (wild‐established, not commercially available), and overlapping species (both). By analyzing trait variation and functional diversity across herbs, shrubs, and trees, we provide a first quantitative portrait of the functional possibilities available to urban plant biodiversity prior to local assembly.

Across all growth forms, trait variation was structured along a slow‐fast axis and an environmental niche axis (Díaz et al. 2016; Reich 2014), with differences among species groups being substantially stronger along the latter. Commercial species were generally associated with warmer and drier conditions. This pattern may reflect horticultural selection for such species (Géron et al. 2021; Kendal et al. 2012), but could also partly arise because species with particular environmental preferences are less likely to establish under current Swiss conditions and therefore remain commercial‐only. Indeed, moisture preference was associated with wild establishment among non‐native species, although temperature preference was not. Functional indices further showed that commercial species consistently had the lowest functional richness, dispersion, and originality, with established species generally showing the highest values except for functional richness and originality in shrubs. Finally, among overlapping species, warmer temperature preference and anthropogenic tolerance were associated with invasive status, linking human selection to potential ecological impacts beyond cultivation.

### 4.1 Composition of the Regional Species Pool

The composition of the regional species pool varied markedly across growth forms. While 60% of shrubs and 68% of trees were strictly commercial, only 18% of herbs belonged to this group. This imbalance suggests that horticultural supply plays a much greater role in shaping the potential species pool for woody plants than for herbs. Although most studies of urban plant diversity focus on realized communities rather than potential species pools, their findings align with this pattern. For example, tree species richness in Swiss urban forests was over 17 times higher than in surrounding natural forests, driven largely by non‐native introductions (Augustinus et al. 2024), while cultivated species in urban yards tend to reflect horticultural offerings and purchasing behavior (Avolio et al. 2018; Cavender‐Bares et al. 2020). Together, these findings highlight the importance of commercial availability in determining which species, particularly woody species, are available to contribute to urban plant communities.

The prominence of commercial trees and shrubs likely reflects contrasting pathways through which woody and herbaceous species enter the regional pool. Woody plants face greater barriers to spontaneous establishment, including limited rooting space, long juvenile phases, and high early mortality (Del Tredici 2010; Wattenhofer and Johnson 2021), while their size, longevity, and multifunctional roles, including shade, esthetics, and climate regulation, make intentional selection and planting particularly important (Avolio et al. 2018; Roman et al. 2018; Turner‐Skoff and Cavender 2019). Herbaceous species, by contrast, often have shorter generation times, high fecundity, and lower space requirements, facilitating spontaneous colonization of disturbed urban environments (Gao et al. 2021). Consequently, horticultural availability may have a particularly strong influence on the woody regional pool, whereas spontaneous establishment contributes more strongly to the herbaceous pool. This distinction also has practical implications: herbaceous diversity may be supported by maintaining colonization opportunities such as unsealed soils and early‐successional habitats (Del Tredici 2010; Hou et al. 2023), whereas the diversity of woody species available for urban planting depends more directly on the breadth of horticultural supply. Given that the woody regional pool is dominated by commercial‐only species, functional biases within this pool could therefore limit the range of functional strategies available for selection and, potentially, influence subsequent local community assembly.

### 4.2 Functional Patterns Across Major Ecological Axes

Regional species pool variation aligned with two major ecological axes. PC1 broadly captured the slow‐fast spectrum, reflecting trade‐offs in growth rate, resource demand, and life‐history strategy (Díaz et al. 2016; Grime 1977; Reich 2014), while PC2 reflected environmental niche differentiation, particularly moisture preference for herbs and temperature preference for shrubs and trees. Although commercial, established, and overlapping species differed significantly along both axes, effect sizes were relatively small for PC1 but consistently large for PC2 across all three growth forms. This suggests that the three groups are distinguished primarily by their environmental niche preferences rather than by a general shift along the slow‐fast spectrum.

Differences along PC1 were comparatively weak and varied among growth forms. Commercial herbs tended toward faster, more resource‐demanding and disturbance‐tolerant strategies, whereas established and overlapping herbs tended toward more conservative strategies. Commercial herbs also had the highest proportion of ornamental species, suggesting that their position along the slow–fast spectrum may partly reflect horticultural preferences. Ornamental plants are widely used in urban green spaces for their esthetic value and can also contribute to human well‐being and ecosystem services such as pollination (Erickson et al. 2022; Francini et al. 2022). Among trees, however, the pattern was reversed: commercial species tended toward more conservative strategies and were more frequently evergreen, whereas established and overlapping trees were more frequently deciduous. This distinction may reflect the different characteristics valued in woody plants, such as year‐round foliage (Li et al. 2024). Overall, these contrasting patterns suggest that horticultural availability does not consistently favor either fast or slow strategies, but may instead reflect different horticultural preferences among growth forms.

Species groups diverged much more strongly along PC2. Commercial species were generally associated with drier conditions among herbs and warmer conditions among shrubs and trees, suggesting that environmental preferences are an important dimension along which commercial species differ from established ones. This pattern may partly reflect horticultural selection for species able to tolerate urban heat and water limitation while requiring less maintenance, such as irrigation (Cavender‐Bares et al. 2020; Jenerette et al. 2016). Such traits may become increasingly relevant under projected warmer and drier conditions across much of Europe, including Switzerland (Vittoz et al. 2013). However, the distinction between commercial and established species may not arise from horticultural selection alone. As explored in the following section, differences in environmental preferences could also partly reflect which non‐native species are able to establish under current Swiss conditions.

### 4.3 Trait Associations With Wild Establishment and Invasive Status

Having established how commercial, established, and overlapping species differ functionally, we further examined whether some of these differences could reflect differential wild establishment rather than horticultural selection alone. Among non‐native species, wild establishment was associated with several environmental, reproductive, and ecological traits. Species associated with moister conditions, sexual reproduction, greater reproductive/pollination/dispersal generalism, broader ecological niches, higher anthropogenic tolerance, higher SLA, and greater winter hardiness had higher odds of establishment, while higher LDMC was associated with lower establishment odds. These associations suggest that the functional differentiation among commercial and established species may arise from multiple processes: horticultural trade determines which non‐native species are introduced and available for planting, while subsequent establishment may further filter this pool according to species traits. Our observational analysis cannot disentangle the relative contributions of these processes, but it shows that differences between commercial and established species should not be attributed to horticultural selection alone. This is particularly relevant for interpreting differences along PC2. Higher moisture preference was associated with establishment and may therefore partly contribute to the drier affinities of commercial herbs: species associated with drier conditions may be more likely to remain commercial‐only, whereas those associated with moister conditions are more likely to enter the established pool. In contrast, temperature preference was not associated with establishment, suggesting that the warmer affinities of commercial woody species cannot simply be explained by temperature‐related differences in establishment success.

Among overlapping species, anthropogenic tolerance and warmer temperature preference were the only traits associated with invasive status. The association with temperature is particularly notable given that warmer temperature preferences also characterized the commercial woody pool. Although temperature preference was not associated with wild establishment, once species were both commercially available and established, warmer‐affinity species were more likely to be invasive. This is relevant under continued climate warming, as alien species in temperate European cities often originate from warmer regions and changing climatic conditions may facilitate their persistence and spread beyond urban environments (Géron et al. 2021). More broadly, traits that allow species to tolerate urban and disturbed environments have also been linked to the establishment and spread of non‐native plants (Bradley et al. 2010; Vilà et al. 2007). These associations are particularly important in the context of horticultural trade, which represents a major pathway for plant introductions and invasions through the widespread cultivation of alien species (Hulme et al. 2018). Thus, while some of the environmental characteristics represented within the commercial pool may be valuable under warmer urban conditions, they may also warrant consideration in relation to invasion risk.

Importantly, the traits associated with wild establishment and invasive status were only partly overlapping. Establishment was associated with a broader range of environmental, reproductive, and ecological traits, whereas invasive status was associated specifically with anthropogenic tolerance and warmer temperature preference. This suggests that the traits associated with successful establishment may differ from those associated with invasive status once establishment has occurred. Given the small number of invasive species (*n* = 25), however, the latter associations should be considered exploratory.

### 4.4 Functional Diversity Across the Regional Species Pool

Functional diversity showed a consistent pattern across growth forms. After accounting for differences in species richness through rarefaction, commercial species had the lowest functional richness, dispersion, and originality among herbs, shrubs, and trees. Established species generally showed the highest values, although overlapping shrubs had the highest functional richness and originality, while functional originality was similar between established and overlapping trees. Overall, these patterns indicate that commercial species occupy a smaller volume of trait space, are more clustered within that space, and contain fewer functionally distinctive species than the established and overlapping groups.

The consistently lower functional diversity of commercial species may reflect horticultural selection for particular combinations of traits suited to common urban planting requirements. Preferences for characteristics such as tolerance of heat and water limitation and reduced maintenance requirements could favor functionally similar species within the commercial pool (Cavender‐Bares et al. 2020; Jenerette et al. 2016), consistent with the differentiation observed along PC2. Such functional convergence may reduce the range of ecological strategies represented among species available through horticulture and potentially limit opportunities for complementary ecosystem functions (Blüthgen and Klein 2011). At the same time, the greater functional richness, dispersion, and originality generally found among established and overlapping species show that substantially broader functional possibilities exist within the regional species pool. This highlights potential opportunities to broaden the functional diversity represented in horticultural supply by considering currently underrepresented strategies from the wider regional pool. However, greater functional diversity alone does not indicate that individual species are suitable for planting, particularly given the presence of invasive species within the overlapping group and the associations with invasive status identified here. Any expansion of the planting palette would therefore need to consider functional diversity alongside ecological suitability and invasion risk.

### 4.5 Limitations and Future Directions

This study provides a first step toward understanding the functional structure of the regional species pool before local community assembly occurs. However, our analyses characterized the species and functional strategies potentially available to Swiss urban communities rather than the species that are ultimately planted or established at individual sites. A logical next step is therefore to examine how environmental, management, and socio‐economic filters act on this regional pool to shape realized urban communities. Combining regional‐pool data with fine‐scale information on planting decisions, environmental conditions, and management regimes would help distinguish the relative influence of ecological filtering and human selection on urban plant composition (Kendal et al. 2012). Ultimately, linking these stages of assembly would allow us to assess whether the functional biases identified in the regional pool are retained, strengthened, or weakened at the local community level.

Several limitations of the underlying species and trait data should also be considered. Commercial availability does not necessarily reflect actual planting frequency, as species offered through horticultural sources may differ substantially in how commonly they are purchased and planted. Moreover, our commercial species pool represents a snapshot of horticultural availability based on the data sources included here and is therefore unlikely to capture the full or temporally dynamic range of species available to consumers and practitioners. Future studies could incorporate additional sources, including online vendors and international suppliers, and track changes in horticultural offerings over time. Trait‐data availability represents a further limitation. Missing trait values required substantial imputation, and cultivars could not be incorporated at the same level of detail, despite their importance in horticultural trade. This reflects a broader limitation of existing plant trait databases (Cornwell et al. 2019) and highlights the need for targeted measurements of traits relevant to urban environments, including at the cultivar level. Expanding social‐ecological trait information related to how plants are selected, managed, and used would further strengthen trait‐based approaches to urban vegetation (Chang et al. 2024).

Finally, the establishment and invasion analyses extend our description of the regional pool by identifying traits associated with different species statuses, but they remain observational and should not be interpreted as demonstrating that these traits cause establishment or invasion. This is particularly important for the invasion analysis, which was based on only 25 invasive species and should therefore be regarded as exploratory. Extending these analyses across larger species pools, regions, and time periods would allow the associations identified here to be tested more robustly and could help determine whether traits associated with establishment and invasion are consistent across environmental and horticultural contexts. More broadly, linking horticultural availability and regional species pools to realized urban functional diversity and ecosystem functions would provide an important next step toward understanding the ecological consequences of horticultural trade and identifying opportunities to broaden the functional strategies available for urban planting.

## 5 Conclusion

Our results show that the regional species pool available to Swiss urban environments is not shaped by ecological processes alone, but may already be functionally structured through horticultural supply. Commercial species represented a narrower and less distinctive range of functional strategies, while establishment and invasion analyses showed that traits may also be associated with which species persist and spread beyond cultivation. Together, these findings highlight the regional species pool as an important, yet often overlooked, stage at which human and ecological processes shape the functional possibilities available for local urban community assembly. Broadening functional diversity within horticultural supply, while considering ecological suitability and invasion risk, may therefore expand these possibilities.

## Author Contributions

**Vivien Grothe:** conceptualization (equal), data curation (equal), formal analysis (lead), investigation (equal), methodology (equal), software (lead), visualization (lead), writing – original draft (equal), writing – review and editing (equal). **Gala Mona Louise Dädlow:** data curation (equal), writing – review and editing (equal). **Joan Casanelles‐Abella:** data curation (equal), writing – review and editing (equal). **Sebastian Ruile:** data curation (equal), writing – review and editing (equal). **Marco Moretti:** conceptualization (supporting), data curation (equal), funding acquisition (lead), project administration (equal), supervision (equal), writing – review and editing (equal). **Bertrand Fournier:** conceptualization (equal), data curation (equal), formal analysis (supporting), investigation (equal), methodology (equal), project administration (equal), software (supporting), supervision (equal), writing – original draft (supporting), writing – review and editing (equal).

## Funding

This study was funded by the Swiss National Science Foundation (SNF) Synergia Program within the project Plants and People in Urban Systems (PAPPUS) (grant number: 213584). Joan Casanelles‐Abella was supported by the SNF 325 Postdoc. Mobility fellowship (grant number: 217754).

## Conflicts of Interest

The authors declare no conflicts of interest.

## Supporting information


**Appendix S1:** Summary of plant traits included in the study.
**Appendix S2:** Performance of five imputation methods used to complete plant trait data.
**Appendix S3:** PCA summary statistics.
**Appendix S4:** Kruskal–Wallis and Dunn's Post Hoc Test results.
**Appendix S5:** Functional diversity indices.
**Appendix S6:** Firth penalized logistic regression models results.
**Appendix S7:** References for individual contributing datasets within the TRY Plant Trait database.

**Appendix S8:** Mean trait values table.

## Data Availability

The trait data underlying this study were compiled from multiple sources, some of which are subject to third‐party redistribution restrictions. Therefore, the compiled trait dataset is available upon reasonable request, while the R code and accompanying metadata are openly available. All materials are deposited in the EnviDat repository: https://www.doi.org/10.16904/envidat.745.
