# Supplementary material for: The Human‐Imprinted Regional Species Pool: Functional Trait Patterns of Commercial and Established Plants
Source: Ecol Evol. 2026 Nov 1;16(11):e74610. doi: 10.1002/ece3.74610 (PMC13724295; doi:10.1002/ece3.74610)
Supplement: Supplementary file 1 [file ECE3-16-e74610-s002.docx]

Supporting information to the paper

Grothe et al. (2026), The Human-Imprinted Regional Species Pool: Functional Trait Patterns of Commercial and Established Plants. *Ecology and Evolution.*

**Appendix S1.** Summary of plant traits included in the study, providing brief descriptions and the sources from which each trait was obtained (TRY, BIEN, iGarten, TPPT, Flora Indicativa, and Botanica; see Methods section for full citations). Additional details on data types and the full set of possible values are provided in the metadata accompanying the dataset in the public data repository.

| Trait Category | Trait | Source | Description |
| --- | --- | --- | --- |
| Environment / Niche | Temperature | Flora Indicativa | Landolt indicator value indicating a species’ relative preference for mean growing-season temperature along the Swiss elevational gradient. Values range from cold, high-elevation conditions to warm, low-elevation conditions. |
|  | Light | Flora Indicativa | Landolt indicator value indicating a species’ light preference in its typical habitat in Switzerland. Values range from deeply shaded to fully exposed conditions. |
|  | Moisture | Flora Indicativa | Landolt indicator value indicating a species’ preference for soil moisture during its growing season in Switzerland. **Values range from very dry to permanently flooded conditions.** |
|  | Soil pH | Flora Indicativa | Landolt indicator value indicating a species’ preference for soil pH in its typical habitat in Switzerland. **Values range from acidic to alkaline soils.** |
|  | Nutrients | Flora Indicativa | Landolt indicator value indicating a species’ preference for soil nutrient availability in its typical habitat in Switzerland.  Values range from very nutrient-poor to very nutrient-rich soil conditions. |
|  | Humus | Flora Indicativa | Landolt indicator value indicating a species’ preference for soil humus content in its typical habitat in Switzerland. **Values range from little or no humus to high humus content in the soil.** |
|  | Aeration | Flora Indicativa | Landolt indicator value indicating a species’ preference for soil aeration in its typical habitat in Switzerland. **Values range from poorly aerated to well-aerated soil conditions.** |
|  | Winter Hardiness | TRY, iGarten & Botanica | Indicates the minimum winter temperature a species can tolerate, based on USDA hardiness zones. Low values correspond to species that are more cold-tolerant, high values reflect species that are less cold-tolerant.  Unit: degrees Celsius (°C) |
|  | Niche Generalism | Flora Indicativa | **Composite index of ecological niche breadth derived from variability in the seven Landolt indicator traits.** For each indicator, Flora Indicativa provides the degree of variation (i.e., whether a species occurs within a narrow or broad range of indicator values), and this index equals the number of indicators with large variation (range 0–7). Higher values indicate niche generalism, whereas lower values indicate niche specialization. |
|  | Ecological Groups | Flora Indicativa | Broad classification of the primary habitat types in which a species typically occurs in Switzerland. |
|  |  |  |  |
|  |  |  |  |
|  |  |  |  |
|  |  |  |  |
| Life History / Growth Strategy | CSR - Competitive | Flora Indicativa | Ecological strategy based on CSR theory (Grime, 1977). Describes species with a high capacity to compete for resources. Note that a species can employ more than one CSR strategy. |
|  | CSR – Stress-Tolerance | Flora Indicativa | Ecological strategy based on CSR theory (Grime, 1977). Describes species adapted to environments with frequent stress. Note that a species can employ more than one CSR strategy. |
|  | CSR - Ruderal | Flora Indicativa | Ecological strategy based on CSR theory (Grime, 1977). Describes species adapted to high disturbance environments. Note that a species can employ more than one CSR strategy. |
|  | Woodiness | TRY, BIEN, iGarten & Botanica | Indicates whether a species develops lignified (woody) stems and branches. |
|  | Life Span | iGarten | Indicates within how many seasons a species completes its life cycle (i.e., annual, biennial, perennial). |
|  |  |  |  |
|  |  |  |  |
|  | Photosynthesis Pathway | TRY | **Primary photosynthetic pathway of a species.** |
|  |  |  |  |
|  | In-Situ Clustering | Flora Indicativa | **Typical spatial growth pattern of the species in its natural habitat, indicating whether it occurs scattered, in groups, or forms large dominant stands.** |
|  | Seed Mass | TRY & BIEN | Seed dry mass.  Unit: milligrams (mg). |
| Morphology | Flower Colour | TRY, BIEN, iGarten & Botanica | Primary flower colour of a species. |
|  | Flower Multicolourity | TRY, BIEN, iGarten & Botanica | **Indicates whether the flowers of a species are single-coloured or multi-coloured.** |
|  | Flower Symmetry | TRY, iGarten & Botanica | **Indicates the symmetry of a species’ flowers.** |
|  | Fleshy Fruit | TRY, BIEN, iGarten & Botanica | Indicates whether a species produces fleshy or dry fruits. |
|  | Leaf Compoundness | TRY, iGarten | Indicates whether a species has simple or compound leaves. |
|  | Leaf Phenology | TRY, iGarten, Flora Indicativa & Botanica | Indicates whether a species retains its leaves year-round (evergreen) or sheds them seasonally (deciduous). |
|  | Fall Colouration | TRY, iGarten | Indicates whether a species exhibits visually colourful foliage during autumn. |
|  | Plant Height | TRY, Bien, iGarten & Botanica | Vegetative plant height.  Unit: meters. |
| Reproduction/Pollination/ Dispersal | Dispersal Syndrome | TRY & Flora Indicativa | Primary dispersal mechanism of a species. |
|  |  |  |  |
|  |  |  |  |
|  |  |  |  |
|  |  |  |  |
|  | Pollination Syndrome | TRY, BIEN & Flora Indicativa | Primary pollination mechanism of a species. |
|  |  |  |  |
|  |  |  |  |
|  |  |  |  |
|  |  |  |  |
|  | Reproduction Type | TRY & iGarten | Primary reproduction mode of a species. |
|  |  |  |  |
|  | Reproduction/ Pollination/ Dispersal Generalism | TRY, BIEN, Flora Indicativa & iGarten | Composite index of strategy breadth derived from accessory variability traits associated with reproduction type, pollination syndrome, and dispersal syndrome. This index equals the number of these three traits in which a species shows variation (range 0–3), with higher values indicating generalism and lower values indicating specialization. |
|  | Flowering Start | Flora Indicativa | Indicates the month when a species usually starts flowering. |
|  | Flowering Duration | Flora Indicativa | **Number of months a species usually flowers.** |
| Physiology | SLA | TRY & BIEN | Specific leaf area (SLA), defined as the ratio of leaf area to leaf dry mass.  Unit: square millimetres per milligram (mm²/mg). |
|  | Leaf Nitrogen Content per Leaf Dry Mass | TRY & BIEN | Leaf nitrogen content per leaf dry mass.  Unit: milligrams per gram (mg/g). |
|  | LDMC | TRY & BIEN | Leaf dry matter content (LDMC), defined as the ratio of leaf dry mass to leaf fresh mass.  Unit: grams per gram (g/g). |
|  | Toxicity | TRY, iGarten, Flora Indicativa & TPPT | Indicates whether any part of a species is reported as toxic. A species is classified as toxic if it is highly toxic, minimally toxic, phototoxic, or allergenic. |
| Human-Related | Human Usage | TRY & iGarten | Documented human use of a species. |
|  |  |  |  |
|  |  |  |  |
|  |  |  |  |
|  |  |  |  |
|  | Human Disturbance Tolerance | Flora Indicativa | Indicates a species’ tolerance to human disturbance, ranging from urbanophobous (close to natural conditions) to urbanophilous (associated with human-influenced environments). |

Grothe et al. (2026), The Human-Imprinted Regional Species Pool: Functional Trait Patterns of Commercial and Established Plants. *Ecology and Evolution.*

**Appendix S2.** Performance of five imputation methods used to complete plant trait data. The column “% Coverage” indicates the proportion of observed (non-missing) values in the original, trait dataset prior to imputation. Imputation performance is assessed using R² and RMSE for continuous traits, and Cohen’s kappa and accuracy for categorical traits. Higher R², kappa, and accuracy values and lower RMSE values indicate better imputation performance. For each trait, the best-performing values across methods are shown in bold.

| **Data Type** | **Trait** | **Coverage (%)** | **Method** | **r2** | **RMSE** | **Kappa** | **Accuracy** |
| --- | --- | --- | --- | --- | --- | --- | --- |
| Continuous | Plant Height | 82.43 | mean | 0.000 | 1.481 | / | / |
|  |  |  | pmm | 0.455 | 1.167 | / | / |
|  |  |  | cart | 0.446 | 1.195 | / | / |
|  |  |  | rf | 0.369 | 1.287 | / | / |
|  |  |  | missForest | **0.715** | **0.791** | / | / |
|  | Seed Mass | 68.76 | mean | 0.000 | 2.318 | / | / |
|  |  |  | pmm | 0.221 | 2.400 | / | / |
|  |  |  | cart | 0.160 | 2.546 | / | / |
|  |  |  | rf | 0.097 | 2.821 | / | / |
|  |  |  | missForest | **0.418** | **1.771** | / | / |
|  | SLA | 48.46 | mean | 0.000 | 0.841 | / | / |
|  |  |  | pmm | 0.034 | 1.556 | / | / |
|  |  |  | cart | 0.065 | 0.877 | / | / |
|  |  |  | rf | 0.017 | 1.078 | / | / |
|  |  |  | missForest | **0.144** | **0.783** | / | / |
|  | Leaf Nitrogen Content per Leaf Dry Mass | 27.98 | mean | 0.000 | 0.367 | / | / |
|  |  |  | pmm | 0.185 | 0.425 | / | / |
|  |  |  | cart | 0.130 | 0.471 | / | / |
|  |  |  | rf | 0.033 | 0.538 | / | / |
|  |  |  | missForest | **0.382** | **0.289** | / | / |
|  | LDMC | 41.64 | mean | 0.000 | 0.518 | / | / |
|  |  |  | pmm | 0.147 | 0.532 | / | / |
|  |  |  | cart | 0.171 | 0.509 | / | / |
|  |  |  | rf | 0.053 | 0.659 | / | / |
|  |  |  | missForest | **0.373** | **0.412** | / | / |
|  | Winter Hardiness | 61.3 | mean | 0.000 | 10.352 | / | / |
|  |  |  | pmm | 0.017 | 13.742 | / | / |
|  |  |  | cart | 0.004 | 14.160 | / | / |
|  |  |  | rf | 0.005 | 14.506 | / | / |
|  |  |  | missForest | **0.115** | **10.162** | / | / |
| Categorical | Temperature | 61.92 | mean | / | / | 0.000 | 0.253 |
|  |  |  | pmm | / | / | 0.023 | 0.159 |
|  |  |  | cart | / | / | 0.129 | 0.253 |
|  |  |  | rf | / | / | 0.135 | 0.262 |
|  |  |  | missForest | / | / | **0.227** | **0.335** |
|  | Light | 65.17 | mean | / | / | 0.000 | 0.510 |
|  |  |  | pmm | / | / | 0.151 | 0.480 |
|  |  |  | cart | / | / | 0.226 | 0.532 |
|  |  |  | rf | / | / | 0.237 | 0.548 |
|  |  |  | missForest | / | / | **0.426** | **0.667** |

|  | Moisture | 63.72 | mean | / | / | 0.000 | 0.237 |
| --- | --- | --- | --- | --- | --- | --- | --- |
|  |  |  | pmm | / | / | 0.067 | 0.207 |
|  |  |  | cart | / | / | 0.086 | 0.221 |
|  |  |  | rf | / | / | 0.079 | 0.219 |
|  |  |  | missForest | / | / | **0.268** | **0.376** |
|  | Soil pH | 63.21 | mean | / | / | 0.000 | 0.801 |
|  |  |  | pmm | / | / | 0.131 | 0.692 |
|  |  |  | cart | / | / | 0.177 | 0.719 |
|  |  |  | rf | / | / | 0.083 | 0.729 |
|  |  |  | missForest | / | / | **0.295** | **0.794** |
|  | Nutrients | 64.81 | mean | / | / | 0.000 | 0.354 |
|  |  |  | pmm | / | / | 0.016 | 0.324 |
|  |  |  | cart | / | / | 0.221 | 0.464 |
|  |  |  | rf | / | / | 0.198 | 0.459 |
|  |  |  | missForest | / | / | **0.407** | **0.602** |
|  | Aeration | 64.28 | mean | / | / | 0.000 | 0.537 |
|  |  |  | pmm | / | / | 0.276 | 0.568 |
|  |  |  | cart | / | / | 0.427 | 0.659 |
|  |  |  | rf | / | / | 0.415 | 0.658 |
|  |  |  | missForest | / | / | **0.595** | **0.764** |
|  | Photosynthesis Pathway | 72.29 | mean | / | / | 0.000 | **0.979** |
|  |  |  | pmm | / | / | 0.143 | 0.948 |
|  |  |  | cart | / | / | 0.169 | 0.956 |
|  |  |  | rf | / | / | -0.017 | 0.965 |
|  |  |  | missForest | / | / | **0.310** | 0.978 |
|  | Life Span | 43.02 | mean | / | / | 0.000 | 0.904 |
|  |  |  | pmm | / | / | 0.405 | 0.895 |
|  |  |  | cart | / | / | 0.368 | 0.886 |
|  |  |  | rf | / | / | 0.380 | 0.910 |
|  |  |  | missForest | / | / | **0.621** | **0.945** |
|  | Flower Colour | 92.84 | mean | / | / | 0.000 | 0.287 |
|  |  |  | pmm | / | / | 0.027 | 0.219 |
|  |  |  | cart | / | / | 0.077 | 0.259 |
|  |  |  | rf | / | / | 0.070 | 0.257 |
|  |  |  | missForest | / | / | **0.238** | **0.392** |
|  | Flower Multicolourity | 94.14 | mean | / | / | 0.000 | **0.853** |
|  |  |  | pmm | / | / | 0.125 | 0.786 |
|  |  |  | cart | / | / | 0.149 | 0.786 |
|  |  |  | rf | / | / | 0.041 | 0.816 |
|  |  |  | missForest | / | / | **0.154** | 0.835 |
|  | Flower Symmetry | 94.2 | mean | / | / | 0.000 | 0.775 |
|  |  |  | pmm | / | / | 0.092 | 0.690 |
|  |  |  | cart | / | / | 0.143 | 0.705 |
|  |  |  | rf | / | / | 0.148 | 0.753 |
|  |  |  | missForest | / | / | **0.316** | **0.812** |

|  | Flowering Start | 65.34 | mean | / | / | 0.000 | 0.355 |
| --- | --- | --- | --- | --- | --- | --- | --- |
|  |  |  | pmm | / | / | 0.058 | 0.285 |
|  |  |  | cart | / | / | 0.085 | 0.295 |
|  |  |  | rf | / | / | 0.076 | 0.308 |
|  |  |  | missForest | / | / | **0.280** | **0.461** |
|  | Flowering Duration | 65.34 | mean | / | / | 0.000 | 0.335 |
|  |  |  | pmm | / | / | 0.065 | 0.311 |
|  |  |  | cart | / | / | 0.101 | 0.342 |
|  |  |  | rf | / | / | 0.082 | 0.341 |
|  |  |  | missForest | / | / | **0.216** | **0.441** |
|  | Fleshy/Dry Fruit | 63.87 | mean | / | / | 0.000 | 0.825 |
|  |  |  | pmm | / | / | 0.507 | 0.854 |
|  |  |  | cart | / | / | 0.461 | 0.852 |
|  |  |  | rf | / | / | 0.498 | 0.876 |
|  |  |  | missForest | / | / | **0.572** | **0.892** |
|  | Leaf Compoundness | 99.84 | mean | / | / | 0.000 | **0.877** |
|  |  |  | pmm | / | / | 0.153 | 0.796 |
|  |  |  | cart | / | / | 0.120 | 0.805 |
|  |  |  | rf | / | / | 0.025 | 0.836 |
|  |  |  | missForest | / | / | **0.217** | 0.874 |
|  | Leaf Phenology | 85.83 | mean | / | / | 0.000 | 0.600 |
|  |  |  | pmm | / | / | 0.188 | 0.613 |
|  |  |  | cart | / | / | 0.224 | 0.629 |
|  |  |  | rf | / | / | 0.199 | 0.625 |
|  |  |  | missForest | / | / | **0.442** | **0.736** |
|  | Fall Colouration | 11.69 | mean | / | / | 0.000 | 0.637 |
|  |  |  | pmm | / | / | 0.194 | 0.613 |
|  |  |  | cart | / | / | 0.179 | 0.613 |
|  |  |  | rf | / | / | 0.288 | 0.661 |
|  |  |  | missForest | / | / | **0.438** | **0.734** |
|  | Dispersal Syndrome | 72.54 | mean | / | / | 0.000 | 0.384 |
|  |  |  | pmm | / | / | 0.185 | 0.358 |
|  |  |  | cart | / | / | 0.325 | 0.469 |
|  |  |  | rf | / | / | 0.306 | 0.470 |
|  |  |  | missForest | / | / | **0.481** | **0.602** |
|  | Pollination Syndrome | 57.5 | mean | / | / | 0.000 | 0.677 |
|  |  |  | pmm | / | / | 0.612 | 0.806 |
|  |  |  | cart | / | / | 0.717 | 0.862 |
|  |  |  | rf | / | / | 0.648 | 0.837 |
|  |  |  | missForest | / | / | **0.813** | **0.916** |
|  | Reproduction Type | 67.19 | mean | / | / | 0.000 | 0.756 |
|  |  |  | pmm | / | / | 0.268 | 0.726 |
|  |  |  | cart | / | / | 0.361 | 0.761 |
|  |  |  | rf | / | / | 0.172 | 0.736 |
|  |  |  | missForest | / | / | **0.417** | **0.802** |

|  | Toxicity | 52.84 | mean | / | / | 0.000 | 0.695 |
| --- | --- | --- | --- | --- | --- | --- | --- |
|  |  |  | pmm | / | / | 0.155 | 0.625 |
|  |  |  | cart | / | / | 0.219 | 0.666 |
|  |  |  | rf | / | / | 0.099 | 0.646 |
|  |  |  | missForest | / | / | **0.346** | **0.730** |
|  | Human Usage | 12.14 | mean | / | / | 0.000 | 0.388 |
|  |  |  | pmm | / | / | 0.034 | 0.248 |
|  |  |  | cart | / | / | 0.094 | 0.326 |
|  |  |  | rf | / | / | 0.075 | 0.326 |
|  |  |  | missForest | / | / | **0.234** | **0.434** |
|  | CSR - Competitive | 65.38 | mean | / | / | 0.000 | 0.821 |
|  |  |  | pmm | / | / | 0.389 | 0.824 |
|  |  |  | cart | / | / | 0.463 | 0.837 |
|  |  |  | rf | / | / | 0.332 | 0.829 |
|  |  |  | missForest | / | / | **0.609** | **0.893** |
|  | CSR – Stress-Tolerance | 65.38 | mean | / | / | 0.000 | 0.727 |
|  |  |  | pmm | / | / | 0.495 | 0.798 |
|  |  |  | cart | / | / | 0.458 | 0.786 |
|  |  |  | rf | / | / | 0.565 | 0.828 |
|  |  |  | missForest | / | / | **0.658** | **0.860** |
|  | CSR – Ruderal | 65.38 | mean | / | / | 0.000 | 0.486 |
|  |  |  | pmm | / | / | 0.309 | 0.655 |
|  |  |  | cart | / | / | 0.358 | 0.679 |
|  |  |  | rf | / | / | 0.407 | 0.704 |
|  |  |  | missForest | / | / | **0.544** | **0.773** |
|  | In-Situ Clustering | 65.38 | mean | / | / | 0.000 | 0.415 |
|  |  |  | pmm | / | / | 0.040 | 0.351 |
|  |  |  | cart | / | / | 0.078 | 0.376 |
|  |  |  | rf | / | / | 0.076 | 0.379 |
|  |  |  | missForest | / | / | **0.271** | **0.517** |
|  | Ecological Groups | 40.05 | mean | / | / | 0.000 | 0.323 |
|  |  |  | pmm | / | / | 0.203 | 0.377 |
|  |  |  | cart | / | / | 0.426 | 0.550 |
|  |  |  | rf | / | / | 0.477 | 0.594 |
|  |  |  | missForest | / | / | **0.678** | **0.748** |
|  | Human Disturbance Tolerance | 53.63 | mean | / | / | 0.000 | 0.449 |
|  |  |  | pmm | / | / | 0.180 | 0.433 |
|  |  |  | cart | / | / | 0.340 | 0.551 |
|  |  |  | rf | / | / | 0.301 | 0.535 |
|  |  |  | missForest | / | / | **0.491** | **0.655** |

Grothe et al. (2026), The Human-Imprinted Regional Species Pool: Functional Trait Patterns of Commercial and Established Plants. *Ecology and Evolution.*

## Appendix S3. PCA Summary Statistics

**Table A.** Summary of the first 9 PCs from the PCA of 3,879 herb species based on 48 traits. The table shows the standard deviation, the percentage of variance each PC explains and the cumulative proportion of variance explained. PC1 and PC2 together explain 21.89% of the trait variation; the first 9 PCs explain 50%.

| **PC** | **Standard deviation** | **Proportion of Variance** | **Cumulative Proportion** |
| --- | --- | --- | --- |
| PC1 | 2.4087 | 0.1209 | 0.1209 |
| PC2 | 2.1696 | 0.0981 | 0.2189 |
| PC3 | 1.6308 | 0.0554 | 0.2743 |
| PC4 | 1.6211 | 0.0548 | 0.3291 |
| PC5 | 1.5099 | 0.0475 | 0.3766 |
| PC6 | 1.2933 | 0.0349 | 0.4114 |
| PC7 | 1.2859 | 0.0345 | 0.4459 |
| PC8 | 1.2437 | 0.0322 | 0.4781 |
| PC9 | 1.1499 | 0.0276 | 0.5057 |

**Table B.** Loadings for the first 9 PCs from the PCA of 3,879 herb species based on 48 traits. Each value represents the contribution of a trait to a given PC, with higher absolute values indicating stronger contributions.

| **Trait** | **PC1** | **PC2** | **PC3** | **PC4** | **PC5** | **PC6** | **PC7** | **PC8** | **PC9** |
| --- | --- | --- | --- | --- | --- | --- | --- | --- | --- |
| CSR - Competitive | -0.1101 | 0.0867 | 0.0218 | -0.2239 | 0.2071 | -0.1811 | 0.1463 | -0.0824 | 0.1161 |
| CSR –  Stress-Tolerant | -0.2537 | 0.1973 | -0.0274 | -0.0018 | 0.1401 | 0.1446 | -0.0197 | 0.0913 | -0.0205 |
| CSR - Ruderal | 0.1898 | -0.1962 | 0.0588 | 0.1952 | -0.0062 | 0.1414 | 0.0911 | 0.0656 | -0.0402 |
| Temperature | 0.2116 | -0.2106 | 0.2275 | -0.1274 | 0.2404 | 0.0818 | -0.0668 | 0.2057 | -0.0484 |
| Light | -0.1153 | -0.1541 | 0.0075 | 0.1165 | -0.3414 | 0.0067 | -0.0111 | -0.0157 | -0.2654 |
| Moisture | 0.1250 | 0.3499 | -0.0899 | -0.1004 | -0.1247 | 0.0025 | 0.1602 | 0.0657 | 0.0414 |
| Soil pH | 0.0079 | 0.1028 | 0.0312 | -0.0077 | -0.0754 | 0.0803 | 0.1447 | 0.1184 | 0.0527 |
| Nutrients | 0.2888 | -0.0374 | -0.0205 | -0.0506 | 0.0082 | -0.1896 | 0.1845 | -0.0031 | 0.2014 |
| Humus | 0.1247 | 0.2157 | 0.0746 | -0.1188 | 0.1505 | 0.0825 | 0.2805 | 0.0939 | 0.0515 |
| Aeration | -0.1866 | -0.2620 | 0.0849 | 0.0912 | -0.0889 | -0.0806 | -0.2264 | -0.1453 | -0.0001 |
| Flowering Start | -0.0443 | -0.0075 | -0.0795 | -0.1008 | -0.2933 | -0.2241 | 0.2895 | 0.0165 | -0.1759 |
| Flowering Duration | 0.1373 | -0.0188 | 0.1028 | 0.2474 | 0.0471 | 0.0405 | 0.0136 | -0.0807 | 0.1893 |
| In-Situ Clustering | 0.0147 | 0.2427 | 0.1638 | 0.0162 | -0.0142 | -0.1521 | -0.0631 | -0.0415 | 0.1541 |
| Human-Disturbance Tolerance | 0.2674 | -0.2520 | 0.1296 | -0.0302 | 0.0031 | -0.0487 | 0.0999 | 0.0184 | 0.0002 |
| c3 Photosynthesis | -0.0091 | 0.0772 | -0.1703 | 0.0616 | 0.1111 | 0.1300 | 0.0159 | -0.1180 | -0.0536 |
| Woodiness | -0.0344 | -0.0662 | 0.0457 | -0.2314 | 0.0867 | -0.1243 | -0.1074 | -0.0822 | -0.0751 |
| Perennial | -0.2402 | 0.1651 | -0.0068 | -0.1751 | 0.1989 | -0.0365 | 0.0587 | 0.0566 | 0.0679 |
| Other Flower Colour | -0.0358 | -0.0551 | 0.1527 | -0.1343 | 0.0352 | 0.3264 | 0.3091 | -0.4249 | -0.0369 |
| White Flowers | -0.0076 | 0.0209 | -0.2788 | 0.0295 | 0.0047 | 0.1446 | -0.2271 | 0.3262 | 0.0229 |
| Yellow Flowers | 0.0329 | -0.0398 | -0.0156 | 0.1432 | 0.1069 | -0.4861 | -0.0449 | 0.1501 | -0.0671 |
| Multi-Coloured Flowers | -0.0215 | -0.0848 | 0.0873 | -0.1747 | -0.0560 | 0.2699 | 0.0951 | -0.1808 | -0.0794 |
| Radial Flower Symmetry | -0.0032 | -0.0474 | -0.0919 | 0.0242 | -0.0556 | -0.1958 | 0.0400 | 0.2325 | 0.1061 |
| Fleshy Fruit | 0.0624 | 0.0159 | -0.0959 | -0.2093 | -0.0144 | 0.0089 | -0.2232 | 0.1021 | 0.0985 |
| Compound Leaves | 0.0639 | 0.0344 | -0.1334 | 0.0484 | 0.1498 | -0.1017 | -0.0927 | -0.1609 | -0.2553 |
| Evergreen | -0.1742 | -0.0167 | 0.0466 | -0.0511 | -0.1213 | 0.0479 | -0.1021 | 0.1368 | 0.0882 |
| Fall Colouration | 0.0315 | -0.0849 | -0.0927 | -0.2825 | -0.0922 | -0.1586 | -0.0341 | -0.1609 | -0.0559 |
| Water Dispersal | 0.1001 | 0.2356 | 0.1015 | 0.0848 | -0.0077 | -0.0691 | 0.0125 | 0.0568 | -0.4090 |
| Animal Dispersal | 0.0622 | 0.0395 | -0.0197 | -0.0335 | 0.0164 | 0.0493 | -0.2779 | -0.2147 | 0.4635 |
| Insect Pollination | -0.0513 | -0.1428 | -0.2948 | 0.0124 | 0.2927 | -0.0037 | 0.2044 | 0.0518 | -0.0128 |
| Animal Pollination | 0.0464 | 0.0507 | 0.0679 | 0.0856 | 0.0088 | 0.0069 | -0.1055 | -0.1234 | -0.0326 |
| Sexual Reproduction | 0.1994 | 0.0709 | 0.0732 | 0.2289 | 0.0059 | -0.0529 | 0.0373 | -0.0893 | -0.0870 |
| Toxicity | 0.1264 | -0.0278 | -0.2483 | -0.1478 | 0.2035 | -0.0471 | -0.0649 | -0.1424 | -0.1731 |
| Food Usage | 0.1899 | 0.0827 | 0.1549 | -0.1682 | -0.3279 | 0.0654 | -0.2095 | 0.0202 | 0.0436 |
| Medicinal Usage | 0.1406 | 0.1353 | -0.1276 | -0.0142 | 0.1497 | -0.0398 | -0.1647 | -0.1981 | -0.1799 |
| Ornamental Usage | -0.2516 | -0.1600 | -0.0476 | 0.1527 | 0.1721 | -0.0246 | 0.2902 | 0.1193 | 0.0861 |
| Fertilized Meadow Species | 0.0180 | 0.0320 | 0.0113 | 0.0671 | 0.0596 | -0.0161 | -0.0257 | -0.0268 | 0.1567 |
| Mountain Species | -0.2398 | 0.0703 | -0.2663 | 0.0619 | -0.2783 | -0.0966 | 0.1034 | -0.2174 | 0.0523 |
| Dry Meadow Species | -0.1471 | -0.0786 | 0.2153 | 0.0678 | 0.2744 | 0.1422 | -0.2119 | 0.0989 | -0.1986 |
| Wetland Species | 0.0878 | 0.2886 | 0.1062 | -0.1152 | -0.1144 | 0.0911 | 0.0353 | 0.2368 | -0.2174 |
| Ruderal/Weedy Species | 0.2439 | -0.2736 | 0.0327 | 0.0582 | -0.0911 | -0.0686 | 0.1232 | -0.0030 | 0.0717 |
| Plant Height | 0.1102 | -0.0531 | 0.0258 | -0.3601 | 0.1086 | -0.2204 | 0.0199 | -0.0344 | 0.0182 |
| Seed Mass | 0.0648 | -0.0353 | -0.0726 | -0.1630 | 0.0057 | 0.0035 | -0.1519 | -0.0633 | -0.0986 |
| SLA | 0.1919 | 0.1502 | -0.1826 | 0.0913 | -0.0029 | 0.1557 | -0.0339 | 0.0619 | 0.1684 |
| Leaf Nitrogen Content per Leaf Dry Mass | 0.1925 | 0.0015 | -0.3823 | -0.0401 | -0.0185 | 0.1016 | -0.0588 | -0.0761 | -0.0693 |
| LDMC | -0.1795 | 0.0429 | 0.3652 | -0.0584 | 0.0158 | -0.2311 | -0.1240 | -0.1078 | -0.0275 |
| Winter Hardiness | -0.0018 | -0.1946 | -0.0679 | -0.2209 | -0.0318 | 0.1925 | -0.0303 | 0.1371 | -0.0990 |
| Reproduction/ Pollination/ Dispersal Generalism | 0.1521 | 0.1312 | -0.0411 | 0.2227 | 0.1223 | -0.0607 | 0.0035 | -0.1022 | -0.1210 |
| Niche Generalism | 0.0233 | 0.1519 | 0.0884 | 0.2186 | 0.0739 | -0.0332 | -0.0599 | -0.2469 | 0.0220 |

**Table C.** Summary of the first 7 PCs from the PCA of 921 shrub species based on 47 traits. The table shows the standard deviation, the percentage of variance each PC explains and the cumulative proportion of variance explained. PC1 and PC2 together explain 22.4% of the trait variation; the first 7 PCs explain 46%.

| **PC** | **Standard deviation** | **Proportion of Variance** | **Cumulative Proportion** |
| --- | --- | --- | --- |
| PC1 | 2.4039 | 0.1230 | 0.1230 |
| PC2 | 2.1791 | 0.1010 | 0.2240 |
| PC3 | 1.8644 | 0.0740 | 0.2979 |
| PC4 | 1.5485 | 0.0510 | 0.3490 |
| PC5 | 1.4251 | 0.0432 | 0.3922 |
| PC6 | 1.3764 | 0.0403 | 0.4325 |
| PC7 | 1.3025 | 0.0361 | 0.4686 |

**Table D.** Loadings for the first 7 PCs from the PCA of 921 shrub species based on 47 traits. Each value represents the contribution of a trait to a given PC, with higher absolute values indicating stronger contributions.

| **Trait** | **PC1** | **PC2** | **PC3** | **PC4** | **PC5** | **PC6** | **PC7** |
| --- | --- | --- | --- | --- | --- | --- | --- |
| CSR - Competitive | -0.0319 | -0.0389 | -0.0280 | 0.0756 | -0.1522 | 0.0762 | -0.0719 |
| CSR - Stress-Tolerant | 0.2676 | 0.2130 | 0.0353 | 0.1233 | 0.0821 | -0.1045 | 0.0520 |
| CSR - Ruderal | 0.0340 | 0.0668 | 0.2474 | -0.1583 | 0.2806 | -0.0797 | -0.0840 |
| Temperature | -0.0117 | -0.3444 | 0.1006 | -0.0180 | 0.1398 | -0.0415 | 0.2497 |
| Light | 0.2687 | 0.1122 | 0.0930 | 0.0244 | 0.0896 | -0.1105 | -0.0127 |
| Moisture | -0.2925 | 0.0890 | -0.0983 | -0.2494 | -0.0403 | -0.0238 | -0.1694 |
| Soil pH | -0.0118 | 0.1756 | -0.1901 | -0.2382 | 0.1203 | -0.0884 | 0.1077 |
| Nutrients | -0.2696 | -0.0713 | 0.1820 | -0.0590 | 0.0189 | 0.0161 | -0.0848 |
| Humus | -0.0935 | 0.1443 | -0.1898 | -0.2810 | 0.1548 | -0.0945 | 0.0973 |
| Aeration | 0.1713 | -0.1429 | 0.1376 | 0.3111 | -0.0977 | 0.0065 | 0.0408 |
| Flowering Start | 0.0241 | 0.1940 | 0.2076 | -0.0325 | -0.0479 | -0.1498 | -0.0671 |
| Flowering Duration | 0.1177 | 0.1314 | 0.1386 | -0.0379 | 0.1948 | 0.0804 | -0.0125 |
| In-Situ Clustering | 0.0663 | 0.2976 | 0.0033 | 0.0967 | 0.0033 | -0.0410 | 0.1203 |
| Human- Disturbance Tolerance | -0.1791 | -0.2602 | 0.1427 | -0.1822 | 0.0972 | 0.0198 | 0.0326 |
| c3 Photosynthesis | -0.0426 | 0.0193 | -0.0438 | 0.0343 | -0.0967 | 0.0164 | -0.0724 |
| Woodiness | -0.1510 | 0.0009 | -0.1969 | 0.0345 | -0.1849 | 0.1076 | 0.0781 |
| Perennial | 0.0113 | 0.0015 | -0.0960 | 0.0734 | -0.0804 | 0.0223 | -0.0081 |
| Other Flower Colour | 0.1118 | 0.0327 | 0.0885 | -0.2393 | -0.3427 | -0.0457 | 0.2962 |
| White Flowers | -0.1669 | -0.0268 | -0.0981 | 0.2177 | 0.1519 | -0.2024 | -0.1055 |
| Yellow Flowers | 0.0839 | -0.0281 | 0.0424 | 0.0446 | 0.1913 | 0.2874 | -0.2505 |
| Multi-Coloured Flowers | 0.0768 | -0.0469 | 0.0087 | -0.1573 | -0.2084 | -0.0800 | 0.2811 |
| Radial Flower Symmetry | -0.0518 | -0.0368 | -0.0669 | -0.0582 | 0.0336 | -0.2390 | 0.0420 |
| Fleshy Fruit | -0.2276 | 0.0369 | -0.1021 | 0.2964 | 0.2393 | -0.1030 | 0.0516 |
| Compound Leaves | -0.0227 | 0.0599 | 0.0882 | 0.0002 | -0.0939 | 0.2930 | -0.0796 |
| Evergreen | 0.2167 | -0.1406 | -0.1231 | 0.0004 | 0.2581 | -0.0082 | 0.0902 |
| Fall Colouration | -0.2106 | -0.2071 | -0.0800 | -0.1402 | -0.0553 | 0.1211 | -0.1299 |
| Water Dispersal | 0.0374 | 0.1678 | -0.1050 | -0.2031 | 0.0466 | -0.0098 | -0.1281 |
| Animal Dispersal | -0.2177 | 0.0521 | -0.1083 | 0.3353 | 0.1966 | -0.0800 | 0.0550 |
| Insect Pollination | -0.0143 | -0.1313 | 0.0747 | 0.0436 | -0.1965 | -0.3366 | -0.3313 |
| Animal Pollination | -0.0108 | 0.1232 | -0.0610 | -0.0373 | 0.1346 | 0.3692 | 0.2624 |
| Sexual Reproduction | -0.1767 | 0.2259 | 0.0524 | 0.1507 | 0.0298 | -0.0038 | 0.0442 |
| Toxicity | 0.0396 | -0.1987 | 0.0086 | -0.0949 | 0.1516 | 0.1961 | -0.0935 |
| Food Usage | -0.1513 | 0.0512 | 0.1129 | 0.0847 | 0.1502 | -0.1778 | 0.0975 |
| Medicinal Usage | -0.1254 | 0.0818 | 0.1210 | 0.0266 | -0.1563 | 0.0493 | 0.2581 |
| Ornamental Usage | 0.2523 | -0.1064 | -0.1307 | -0.1057 | 0.0015 | 0.0734 | -0.2887 |
| Mountain Species | 0.0803 | 0.1737 | -0.0410 | 0.0112 | -0.1548 | 0.0477 | -0.3038 |
| Dry Meadow Species | 0.2632 | 0.0070 | 0.0142 | 0.1515 | -0.0980 | -0.0052 | 0.1481 |
| Wetland Species | 0.0158 | 0.1643 | -0.1752 | -0.2954 | 0.1338 | -0.1534 | 0.0111 |
| Ruderal/Weedy Species | 0.0341 | 0.0203 | 0.2948 | -0.1267 | 0.2795 | -0.0576 | -0.0108 |
| Plant Height | -0.1837 | -0.1103 | -0.0188 | -0.0177 | 0.0132 | 0.1036 | 0.1302 |
| Seed Mass | -0.0554 | -0.0269 | -0.0474 | 0.0471 | 0.0927 | 0.2338 | 0.1816 |
| SLA | -0.2201 | 0.1269 | 0.2730 | -0.0008 | -0.1590 | 0.0470 | 0.0174 |
| Leaf Nitrogen Content per Leaf Dry Mass | -0.1070 | 0.1015 | 0.3501 | -0.0572 | -0.0184 | 0.1588 | -0.0941 |
| LDMC | 0.0143 | -0.0683 | -0.4272 | 0.0480 | -0.0428 | 0.0298 | -0.0232 |
| Winter Hardiness | 0.1502 | -0.2854 | 0.0436 | -0.0899 | 0.1358 | 0.0528 | 0.0689 |
| Reproduction/Pollination/Dispersal Generalism | -0.0238 | 0.2605 | -0.0623 | -0.0162 | 0.0370 | 0.1980 | -0.0017 |
| Niche Generalism | 0.0291 | 0.1478 | -0.0337 | 0.0968 | 0.0520 | 0.3207 | -0.0939 |

**Table E.** Summary of the first 7 PCs from the PCA of 495 tree species based on 44 traits. The table shows the standard deviation, the percentage of variance each PC explains and the cumulative proportion of variance explained. PC1 and PC2 together explain 22.1% of the trait variation; the first 7 PCs explain 45%.

| **PC** | **Standard deviation** | **Proportion of Variance** | **Cumulative Proportion** |
| --- | --- | --- | --- |
| PC1 | 2.4367 | 0.1349 | 0.1349 |
| PC2 | 1.9508 | 0.0865 | 0.2214 |
| PC3 | 1.6823 | 0.0643 | 0.2858 |
| PC4 | 1.5043 | 0.0514 | 0.3372 |
| PC5 | 1.3521 | 0.0416 | 0.3787 |
| PC6 | 1.3074 | 0.0389 | 0.4176 |

**Table F.** Loadings for the first 6 PCs from the PCA of 495 tree species based on 44 traits. Each value represents the contribution of a trait to a given PC, with higher absolute values indicating stronger contributions.

| **Trait** | **PC1** | **PC2** | **PC3** | **PC4** | **PC5** | **PC6** |
| --- | --- | --- | --- | --- | --- | --- |
| CSR - Stress-Tolerant | 0 | 0.1570 | -0.1811 | 0.0143 | -0.0897 | 0.2642 |
| CSR - Ruderal | 0.0452 | 0.0165 | -0.0807 | 0.3115 | -0.4132 | 0.0216 |
| Temperature | -0.0401 | -0.4209 | 0.0671 | 0.1136 | -0.0659 | -0.1045 |
| Light | -0.0763 | 0.0157 | -0.2019 | 0.1300 | -0.0795 | 0.0184 |
| Moisture | 0.2546 | 0.1939 | 0.1752 | -0.0943 | -0.0346 | -0.1626 |
| Soil pH | -0.0954 | 0.1262 | -0.1130 | -0.0476 | -0.0667 | -0.3085 |
| Nutrients | 0.3209 | 0.0658 | 0.1833 | 0.0609 | -0.0385 | -0.0975 |
| Humus | -0.0602 | 0.1314 | -0.0239 | -0.1669 | -0.1472 | -0.3899 |
| Aeration | 0.0055 | -0.2811 | -0.0759 | 0.0395 | 0.0449 | 0.0970 |
| Flowering Start | 0.1529 | 0.1432 | -0.1262 | 0.1124 | -0.1002 | -0.1272 |
| Flowering Duration | -0.0036 | 0.0393 | -0.3028 | 0.2257 | 0.0736 | -0.0576 |
| In-Situ Clustering | -0.1327 | 0.2851 | -0.0779 | 0.0470 | -0.1115 | -0.0778 |
| Human-Disturbance Tolerance | 0.1760 | -0.2116 | 0.2299 | -0.0457 | -0.1206 | -0.2169 |
| c3 Photosynthesis | 0.0028 | 0.0277 | -0.0073 | -0.0696 | -0.0480 | 0.0460 |
| Other Flower Colour | -0.1267 | 0.0010 | 0.1560 | 0.0831 | -0.2448 | 0.1273 |
| White Flowers | 0.2040 | -0.1015 | -0.2634 | -0.2276 | 0.0630 | 0.0067 |
| Yellow Flowers | 0.0244 | 0.0829 | 0.1317 | 0.1694 | 0.3108 | -0.1194 |
| Multi-Coloured Flowers | -0.0178 | -0.0009 | 0.1193 | 0.0481 | -0.1769 | -0.0154 |
| Radial Flower Symmetry | -0.0489 | -0.0714 | -0.1686 | -0.2016 | -0.2597 | -0.0363 |
| Fleshy Fruit | 0.1962 | -0.1427 | -0.3277 | -0.2246 | -0.0385 | 0.0561 |
| Compound Leaves | 0.0923 | -0.0022 | 0.0252 | 0.2351 | 0.1682 | -0.2385 |
| Evergreen | -0.2878 | -0.1822 | -0.1482 | 0.0052 | 0.0148 | -0.1517 |
| Fall Colouration | 0.2925 | -0.0159 | 0.1305 | -0.0315 | 0.0094 | -0.0426 |
| Water Dispersal | 0.0258 | 0.1952 | -0.0120 | -0.0256 | 0.0296 | 0.0802 |
| Animal Dispersal | 0.1897 | -0.1408 | -0.3251 | -0.2552 | -0.0447 | 0.0017 |
| Insect Pollination | 0.2571 | -0.1883 | -0.1334 | 0.0573 | 0.2123 | 0.0119 |
| Animal Pollination | 0.0539 | 0.1495 | -0.0556 | 0.0235 | 0.0206 | -0.0561 |
| Sexual Reproduction | -0.0423 | 0.0859 | -0.0321 | -0.2937 | -0.0155 | 0.0223 |
| Toxicity | -0.0155 | 0.0035 | -0.1798 | 0.3060 | 0.1265 | -0.2733 |
| Food Usage | 0.1540 | -0.1296 | -0.0791 | -0.0593 | -0.1820 | -0.2546 |
| Medicinal Usage | 0.0804 | 0.0844 | -0.0911 | 0.0570 | -0.1084 | 0.0646 |
| Ornamental Usage | -0.2494 | -0.0322 | -0.1110 | 0.1542 | 0.2478 | 0.0866 |
| Mountain Species | -0.0281 | 0.1669 | -0.1388 | 0.0289 | 0.0754 | 0.0981 |
| Dry Meadow Species | -0.0290 | 0.0002 | -0.1020 | 0.2038 | -0.1865 | 0.1868 |
| Wetland Species | -0.0325 | 0.1695 | -0.0533 | -0.1123 | -0.1093 | -0.2395 |
| Ruderal/Weedy Species | 0.0706 | -0.0175 | -0.0947 | 0.2224 | -0.3684 | 0.0122 |
| Plant Height | -0.2223 | 0.0309 | 0.1892 | -0.0993 | -0.1097 | -0.0759 |
| Seed Mass | 0.0097 | -0.0303 | 0.0237 | 0.0694 | 0.0466 | -0.2385 |
| SLA | 0.2963 | 0.0908 | 0.0976 | 0.1236 | -0.0328 | 0.1232 |
| Leaf Nitrogen Content per Leaf Dry Mass | 0.0811 | 0.0572 | 0.0626 | 0.0719 | 0.0571 | -0.0009 |
| LDMC | -0.2475 | -0.0138 | 0.0041 | -0.2367 | 0.1259 | -0.1513 |
| Winter Hardiness | -0.0939 | -0.2899 | -0.1007 | 0.1906 | -0.0426 | -0.2236 |
| Reproductive/Pollination/ Dispersal Generalism | 0.1877 | 0.1664 | -0.2101 | 0.0822 | 0.2244 | -0.0586 |
| Niche Generalism | -0.0688 | 0.2973 | -0.1793 | 0.0071 | -0.0055 | -0.1291 |

Grothe et al. (2026), The Human-Imprinted Regional Species Pool: Functional Trait Patterns of Commercial and Established Plants. *Ecology and Evolution.*

**Appendix S4**. Kruskal-Wallis and Dunn’s Post-Hoc Test Results

## Table A. Kruskal-Wallis tests comparing principal component (PC) coordinates among commercial, established, and overlapping species for herbs, shrubs, and trees. For each growth form, PC axes are reported up to the number of dimensions selected for representing functional trait space (herbs n = 9, shrubs n= 7, trees n= 6). Kruskal-Wallis tests were used to assess whether PC coordinates differed overall among the three species groups on each axis. χ² denotes the Kruskal-Wallis test statistic, df the degrees of freedom, and *p* the associated significance level. Effect sizes are reported as rank-based eta-squared (η²H) with 95% confidence intervals (CI) and corresponding magnitude classifications (small: 0.01 ≤ η²H < 0.06; moderate: 0.06 ≤ η²H < 0.14; large: η²H ≥ 0.14).

| **Growth Form** | **PC Axis** | **χ²** | **df** | ***p*** | **η²H** | **Magnitude** | **95% CI** |
| --- | --- | --- | --- | --- | --- | --- | --- |
| Herbs | PC1 | 75.841 | 2 | < 0.001 | 0.019 | small | 0.010 – 0.030 |
|  | PC2 | 592.308 | 2 | < 0.001 | 0.152 | large | 0.130 – 0.170 |
|  | PC3 | 37.631 | 2 | < 0.001 | 0.009 | small | 0.004 – 0.020 |
|  | PC4 | 355.363 | 2 | < 0.001 | 0.091 | moderate | 0.07 – 0.110 |
|  | PC5 | 342.703 | 2 | < 0.001 | 0.088 | moderate | 0.07 – 0.110 |
|  | PC6 | 59.28 | 2 | < 0.001 | 0.015 | small | 0.008 – 0.020 |
|  | PC7 | 23.171 | 2 | < 0.001 | 0.005 | small | 0.002 – 0.010 |
|  | PC8 | 57.665 | 2 | < 0.001 | 0.014 | small | 0.008 – 0.020 |
|  | PC9 | 12.133 | 2 | 0.001 | 0.003 | small | 0 – 0.007 |
| Shrubs | PC1 | 9.561 | 2 | 0.008 | 0.008 | small | 0 – 0.03 |
|  | PC2 | 360.209 | 2 | < 0.001 | 0.39 | large | 0.34 – 0.44 |
|  | PC3 | 1.243 | 2 | 0.5 | -0.001 | small | -0.002 – 0.01 |
|  | PC4 | 35.681 | 2 | < 0.001 | 0.037 | small | 0.02 – 0.07 |
|  | PC5 | 8.454 | 2 | 0.014 | 0.007 | small | 0 - 0.02 |
|  | PC6 | 75.928 | 2 | < 0.001 | 0.081 | moderate | 0.05 - 0.13 |
|  | PC7 | 9.434 | 2 | 0.008 | 0.008 | small | 0 – 0.03 |
| Trees | PC1 | 6.725 | 2 | 0.034 | 0.01 | small | -0.002 – 0.04 |
|  | PC2 | 116.595 | 2 | < 0.001 | 0.233 | large | 0.16 – 0.31 |
|  | PC3 | 6.465 | 2 | 0.039 | 0.009 | small | -0.002 – 0.04 |
|  | PC4 | 7.881 | 2 | 0.019 | 0.012 | small | -0.001 – 0.05 |
|  | PC5 | 0.312 | 2 | 0.855 | -0.003 | small | -0.004 – 0.02 |
|  | PC6 | 1.68 | 2 | 0.431 | -0.001 | small | -0.004 – 0.02 |

## Table B. Results of Dunn’s post-hoc tests comparing species coordinates of commercial, established and overlapping species groups along PC1 and PC2 for herbs, shrubs, and trees. Reported are Z-statistics, Holm-adjusted p-values and significance levels (α = 0.05).

| **PC Axis** | **Growth Form** | **Group Comparison** | **Z-Statistic** | **Holm Adjusted P-Value** | **Significance** |
| --- | --- | --- | --- | --- | --- |
| PC1 | Herbs | Commercial vs. Established | 6.19 | 1.23e- 9 | **** |
|  |  | Commercial vs. Overlapping | -8.68 | 1.24e-17 | **** |
|  |  | Established vs. Overlapping | -4.45 | 8.48e- 6 | **** |
|  | Shrubs | Commercial vs. Established | 3.04 | 0.00717 | ** |
|  |  | Commercial vs. Overlapping | -0.197 | 0.844 | ns |
|  |  | Established vs. Overlapping | 2.45 | 0.0290 | * |
|  | Trees | Commercial vs. Established | 2.57 | 0.0303 | * |
|  |  | Commercial vs. Overlapping | -0.723 | 0.470 | ns |
|  |  | Established vs. Overlapping | 2.01 | 0.0880 | ns |
| PC2 | Herbs | Commercial vs. Established | 20.3 | 3.71e- 91 | **** |
|  |  | Commercial vs. Overlapping | -23.4 | 2.52e-120 | **** |
|  |  | Established vs. Overlapping | -8.15 | 3.64e- 16 | **** |
|  | Shrubs | Commercial vs. Established | 14.2 | 2.94e-45 | **** |
|  |  | Commercial vs. Overlapping | -15.8 | 1.46e-55 | **** |
|  |  | Established vs. Overlapping | -0.0948 | 9.24e- 1 | ns |
|  | Trees | Commercial vs. Established | 3.97 | 1.47e- 4 | *** |
|  |  | Commercial vs. Overlapping | -10.5 | 1.70e-25 | **** |
|  |  | Established vs. Overlapping | -1.93 | 0.053 | ns |

Grothe et al. (2026), The Human-Imprinted Regional Species Pool: Functional Trait Patterns of Commercial and Established Plants. *Ecology and Evolution.*

## Appendix S5. Functional diversity indices (richness (FRic), dispersion (FDis), and originality (FOri)) of commercial, established, and overlapping herb, shrub and trees species. Indices were calculated within multidimensional trait spaces constructed using PCoA (*mFD* package). To account for differences in species richness among groups, indices were calculated across 1,000 rarefaction iterations using equal numbers of species per group within each growth form (herbs: n = 600; shrubs: n = 150; trees: n = 25). The table reports mean index values and 95% rarefaction intervals (2.5th and 97.5th percentiles) across iterations.

| **Growth Form** | **Group** | **FRic** | | **FDis** | | **FOri** | |
| --- | --- | --- | --- | --- | --- | --- | --- |
|  |  | **Mean** | **95% Rarefaction Interval** | **Mean** | **95% Rarefaction Interval** | **Mean** | **95% Rarefaction Interval** |
| Herbs | Established | 0.333 | 0.285-0.382 | 0.624 | 0.601-0.647 | 0.256 | 0.234-0.274 |
|  | Overlapping | 0.219 | 0.187-0.251 | 0.563 | 0.542-0.582 | 0.246 | 0.225-0.261 |
|  | Commercial | 0.189 | 0.189-0.162 | 0.551 | 0.532-0.569 | 0.160 | 0.145-0.171 |
| Shrubs | Established | 0.271 | 0.238-0.314 | 0.681 | 0.672-0.706 | 0.259 | 0.211-0.314 |
|  | Overlapping | 0.340 | 0.283-0.392 | 0.667 | 0.652-0.692 | 0.321 | 0.266-0.388 |
|  | Commercial | 0.132 | 0.098-0.172 | 0.625 | 0.600-0.652 | 0.201 | 0.161-0.250 |
| Trees | Established | 0.197 | 0.129-0.277 | 0.739 | 0.687-0.790 | 0.422 | 0.332-0.525 |
|  | Overlapping | 0.139 | 0.063-0.2339 | 0.725 | 0.649-0.807 | 0.428 | 0.333-0.550 |
|  | Commercial | 0.055 | 0.019-0.105 | 0.688 | 0.606-0.759 | 0.314 | 0.222-0.414 |

Grothe et al. (2026), The Human-Imprinted Regional Species Pool: Functional Trait Patterns of Commercial and Established Plants. *Ecology and Evolution.*

## Appendix S6. Results of growth-form-adjusted single-trait Firth penalized logistic regression models. Wild establishment was assessed among non-native species by comparing species established in the wild (established-only and overlapping species) with commercial-only species. Invasive status was assessed among overlapping species (i.e. species that were both established in the wild and commercially available) by comparing invasive with non-invasive species. Odds ratios (ORs) are reported with 95% confidence intervals, together with raw p-values and p-values adjusted for multiple testing within each analysis using the Benjamini–Hochberg procedure (α = 0.05). The invasive-status analysis should be interpreted as exploratory due to the small number of invasive species (n = 25).

| **Analysis** | **Trait** | **Odds ratio (OR)** | **95% CI** | **p-value** | **BH-adjusted p-value** |
| --- | --- | --- | --- | --- | --- |
| Wild Establishment | Temperature | 0.952 | 0.897-1.00 | 0.102 | 0.137 |
|  | Moisture | 1.429 | 1.342-1.524 | <0.001 | <0.001 |
|  | Light | 0.930 | 0.781-1.107 | 0.418 | 0.501 |
|  | SLA | 1.079 | 1.06-1.09 | <0.001 | <0.001 |
|  | LDMC | 0.005 | 0.001-0.018 | <0.001 | <0.001 |
|  | Plant Height | 1.000 | 0.983-1.016 | 0.957 | 0.957 |
|  | Seed Mass | 1.000 | 0.999-1.000 | 0.937 | 0.957 |
|  | Human-Disturbance Tolerance | 1.329 | 1.199-1.475 | <0.001 | <0.001 |
|  | Sexual Reproduction | 2.602 | 2.151-3.154 | <0.001 | <0.001 |
|  | Winter Hardiness | 0.956 | 0.947-0.965 | <0.001 | <0.001 |
|  | Reproduction/Pollination/Dispersal Generalism | 2.266 | 2.038-2.524 | <0.001 | <0.001 |
|  | Niche Generalism | 1.690 | 1.508-1.909 | <0.001 | <0.001 |
| Invasive Status | Temperature | 2.228 | 1.529- 3.524 | <0.001 | <0.001 |
|  | Moisture | 1.016 | 0.802-1.279 | 0.891 | 0.891 |
|  | Light | 0.691 | 0.384-1.273 | 0.232 | 0.316 |
|  | SLA | 1.035 | 0.993-1.066 | 0.091 | 0.182 |
|  | LDMC | 0.005 | 0.000-2.231 | 0.090 | 0.182 |
|  | Plant Height | 1.029 | 0.971-1.073 | 0.263 | 0.316 |
|  | Seed Mass | 1.000 | 0.998-1.000 | 0.395 | 0.431 |
|  | Human-Disturbance Tolerance | 3.018 | 1.816-5.139 | <0.001 | <0.001 |
|  | Sexual Reproduction | 0.537 | 0.235-1.323 | 0.168 | 0.253 |
|  | Winter Hardiness | 1.036 | 0.987-1.088 | 0.146 | 0.250 |
|  | Reproduction/Pollination/Dispersal Generalism | 0.674 | 0.461-0.981 | 0.039 | 0.152 |
|  | Niche Generalism | 0.748 | 0.501-1.000 | 0.050 | 0.152 |

Grothe et al. (2026), The Human-Imprinted Regional Species Pool: Functional Trait Patterns of Commercial and Established Plants. *Ecology and Evolution.*

## Appendix S7. References for individual contributing datasets within the TRY Plant Trait database from which trait data were obtained for this study.

| Dataset | Reference |
| --- | --- |
| Aboveground morphological traits of grassland species | Abakumova, M., Zobel K., Lepik, A., Semchenko, M. (2016) Plasticity in plant functional traits is shaped by variability in neighbourhood species composition. New Phytologist 211:455–463 doi:10.1111/nph.13935 |
| Jasper Ridge Californian Woody Plants Database | Ackerly, D. D. and W. K. Cornwell. 2007. A trait-based approach to community assembly: partitioning of species trait values into within- and among-community components. Ecology Letters 10:135-145. |
| Herbaceous leaf traits database from Mediterranean serpentine and non-serpentine soils | Adamidis GC, Kazakou E, Fyllas NM, Dimitrakopoulos PG (2014) Species adaptive strategies and leaf economic relationships across serpentine and non-serpentine habitats on lesbos, eastern Mediterranean. PLoS One 9:e96034. doi:10.1371/journal.pone.0096034 |
| Fagus sylvatica Paggeo Greece | Adamidis GC, Varsamis G, Tsiripidis I, Dimitrakopoulos PG, Papageorgiou AC. Patterns of Leaf Morphological Traits of Beech (Fagus sylvatica L.) along an Altitudinal Gradient. Forests. 2021; 12(10):1297. https://doi.org/10.3390/f12101297 |
| Functional Traits of Graminoids in Semi-Arid Steppes Database | Adler P. B. (2003) A comparison of livestock grazing effects on sagebrush steppe, USA, and Patagonian steppe, Argentina. PhD Thesis, Colorado State University |
| Functional Traits of Graminoids in Semi-Arid Steppes Database | Adler P. B., D. G. Milchunas, W. K. Lauenroth, O. E. Sala and I. C. Burke (2004) Functional traits of graminoids in semi-arid steppes: a test of grazing histories. Journal of Applied Ecology 2004 41, 653–663 |
| Functional traits explaining variation in plant life history strategies | Adler PB, R Salguero-Gómez, A Compagnoni, JS Hsu, J Ray-Mukherjee, C Mbeau-Ache, M Franco (2014) Functional traits explain variation in plant life history strategies. PNAS 111 (2) 740-745. doi: 10.1073/pnas.1315179111 |
| Ecophy | Albert CH, Thuiller W & Lavorel S, unpub. C.H. Albert PhD - Variabilité fonctionnelle intraspécifique: quantification in situ et implications dans une vallée alpine https://tel.archives-ouvertes.fr/tel-00450219 |
| Guisane2080 | Albert CH, Thuiller W, Yoccoz NG, Soudan A, Boucher F, Saccone P & Lavorel S (2010) Intraspecific functional variability : extent, structure and sources of variation within an alpine valley. Journal of Ecology, 98, 604–613 Albert CH, Thuiller W, Yoccoz NG, Douzet R, Aubert S & Lavorel S (2010) A multi-trait approach reveals the structure and the relative importance of intra- versus interspecific variability. Functional Ecology, 24, 1192–1201 PhD Cécile ALBERT: Variabilité fonctionnelle intraspécifique: quantification in situ et implications dans une vallée alpine - https://tel.archives-ouvertes.fr/tel-00450219 |
| Haean_South_Korea_Traits | Ali, H.E., Reineking, B. & Münkemüller, T. Plant Soil (2017) 411: 359. https://doi.org/10.1007/s11104-016-3036-5 |
| CPCRW Carbon Dynamics Along Permafrost Gradient: Specific Leaf Area of Alder and Spruce | Anderson, C. G., Bond-Lamberty, B., Stegen, J. C. Controls on and consequences of specific leaf area variation with permafrost depth in a boreal forest. Submitted |
| Daintree Rainforest Functional Traits Data | Apgaua, D. M., Tng, D. Y., Cernusak, L. A., Cheesman, A. W., Santos, R. M., Edwards, W. J., & Laurance, S. G. (2017). Plant functional groups within a tropical forest exhibit different wood functional anatomy. Functional ecology, 31(3), 582-591 |
| Daintree Rainforest Functional Traits Data | Apgaua, D.M., Ishida, F.Y., Tng, D.Y., Laidlaw, M.J., Santos, R.M., Rumman, R., Eamus, D., Holtum, J.A. and Laurance, S.G., 2015. Functional traits and water transport strategies in lowland tropical rainforest trees. PloS one, 10(6), p.e0130799 |
| Aspinwall et al. 2019 GCB | Aspinwall MJ, Pfautsch S, Tjoelker MG, Varhammar A, Possell M, Drake JE, Reich PB, Tissue DT, Atkin OK, Rymer PD, Dennison S, Van Sluyter SC. (2019). Range size and growth temperature influence Eucalyptus species responses to an experimental heatwave. Global Change Biology 25, 1665-1684 |
| Plant Physiology Database | Atkin, O. K., M. H. M. Westbeek, M. L. Cambridge, H. Lambers, and T. L. Pons. 1997. Leaf respiration in light and darkness - A comparison of slow- and fast-growing Poa species. Plant Physiology 113:961-965. |
| Plant Physiology Database | Atkin, O. K., M. Schortemeyer, N. McFarlane, and J. R. Evans. 1999. The response of fast- and slow-growing Acacia species to elevated atmospheric CO2: an analysis of the underlying components of relative growth rate. Oecologia 120:544-554. |
| Traits of Plants in Canada (TOPIC) | Aubin, I. & JP Ricard. 1999. Méthodes d’implantation d’espèces compatibles avec le réseau de distribution d’électricité. Méthodes alternatives à la plantation. Mise en place des dispositifs expérimentaux. Étude présentée à l’Unité Environnement – Direction Projets de distribution. Hydro-Québec. 49 p. |
| Traits of Plants in Canada (TOPIC) | Aubin, I. & JP Ricard. 2000. Ensemencement d’espèces compatibles dans les emprises de distribution d’électricité. Étude présentée à l’Unité Environnement – Direction Projets de distribution. Hydro-Québec. 70 p. |
| Traits of Plants in Canada (TOPIC) | Aubin, I. 2010. LTSP - Root and leaf nutrition of understory vegetation. Canadian Forest Service. Unpublished data |
| Traits of Plants in Canada (TOPIC) | Aubin, I., C. Messier, and D. Kneeshaw. 2005. Population structure and growth acclimation of mountain maple along a successional gradient in the southern boreal forest. Ecoscience 12:540-548 // Aubin, I., M. Beaudet, and C. Messier. |
| TOPIC (Traits of Plants in Canada) | Aubin, I., Messier, C., Gachet, S., Lawrence, K., McKenney, D., Arseneault, A., Bell, W., De Grandpré, L., Shipley, B., Ricard, J.P. and Munson, A.D., 2012. TOPIC–traits of plants in Canada. Natural Resources Canada, Canadian Forest Service, Sault Ste. Marie, Ontario. Online [URL] TOPIC website :http://cfs.cloud.nrcan.gc.ca/ctn/topic.php |
| TOPIC (Traits of Plants in Canada) | Aubin, I., Munson, A. D., Cardou, F., Burton, P. J., Isabel, N., … McKenney, D. (2016) Traits to stay, traits to move: a review of functional traits to assess sensitivity and adaptive capacity of temperate and boreal trees to climate change. Environmental Reviews 24:164-186 doi:10.1139/er-2015-0072 |
| Leaf Structure and Chemistry | Auger, S. 2012. MSc thesis, Université de Sherbrooke, Sherbrooke (Quebec) L'importance de la variabilité interspécifique des traits fonctionnels par rapport à la variabilité intraspécifique chez les jeunes arbres en forêt mature. |
| Leaf Structure and Chemistry | Auger, S., Shipley, B. (2012). : Interspecific and intraspecific trait variation along short environmental gradients in an old-growth temperate forest. Journal of Vegetation Science. DOI: 1111/j.1654-1103.2012.01473.x |
| JACARE A-Ci leaf trait data base 2017 | Bahar, NHA, Ishida, FY, Weerasinghe, LK, Guerrieri, R, OSullivan, OS, Bloomfield, KJ, Asner, GP, Martin, RE, Lloyd, J, Malhi, Y, Phillips, OL, Meir, P, Salinas, N, Cosio, EG, Domingues, TF, Quesada, CA, Sinca, F, Escudero Vega, A, Zuloaga Ccorimanya, PP, del Aguila-Pasquel, J, Quispe Huaypar, K, Cuba Torres, I, Butrón Loayza, R, Pelaez Tapia, Y, Huaman Ovalle, J, Long, BM, Evans, JR, Atkin, OK (2017) Leaf-level photosynthetic capacity in lowland Amazonian and high-elevation Andean tropical moist forests of Peru. New Phytologist 214, 1002-1018. |
| European Mountain Meadows Plant Traits Database | Bahn, M., G. Wohlfahrt, E. Haubner, I. Horak, W. Michaeler, K. Rottmar, U. Tappeiner, and A. Cernusca. 1999. Leaf photosynthesis, nitrogen contents and specific leaf area of 30 grassland species in differently managed mountain ecosystems in the Eastern Alps. Pages 247-255 in A. Cernusca, U. Tappeiner, and N. Bayfield, editors. Land-use changes in European mountain ecosystems. ECOMONT- Concept and Results. Blackwell Wissenschaft, Berlin. |
| The RAINFOR Plant Trait Database | Baker, T. R., O.L. Phillips, W.F. Laurance, N.C.A. Pitman, S. Almeida, L. Arroyo, A. DiFiore, T. Erwin, N. Higuchi, T.J. Killeen, S.G. Laurance, H. Nascimento, A. Monteagudo, D.A. Neill, J.N.M. Silva, Y. Malhi, G. Lopez Gonzalez, J. Peacock, C.A. Quesada, S. L. Lewis, and J. Lloyd. 2009. Do species traits determine patterns of wood production in Amazonian forests? Biogeosciences 6:297-307. |
| Colorado Plateau Restoration Species | Balazs K, Andrea Kramer, Seth Munson, Bradley Butterfield. The right trait in the right place at the right time: Matching traits to environment improves restoration outcomes. 2020. Ecological Applications |
| The Bridge Database | Baraloto, C., C. E. T. Paine, L. Poorter, J. Beauchene, D. Bonal, A.-M. Domenach, B. Herault, S. Patino, J.-C. Roggy, and J. Chave. 2010. Decoupled leaf and stem economics in rainforest trees. Ecology Letters 13:1338-1347 |
| The Bridge Database | Baraloto, C., C. E. T. Paine, S. Patino, D. Bonal, B. Herault, and J. Chave. 2010. Functional trait variation and sampling strategies in species-rich plant communities. Functional Ecology 24: 208-216 |
| Specific leaf area of Dodonaea viscosa along an elevational gradient in South Australia | Baruch, Z, Christmas, M, Breed, MF, Guerin, GR, Caddy-Retalic, S, McDonald, J, Jardine, DI, Leitch, EJ, Gellie, N, Hill, K, McCallum, K, Lowe, AJ (2017). Leaf trait associations with environmental variation in the wide-ranging shrub Dodonaea viscosa subsp. angustissima (Sapindaceae). Austral Ecology 43(5): 553-561. (doi:10.1111/aec.12474) |
| Specific leaf area of Dodonaea viscosa along an latitudinal gradient in Australia | Baruch, Z, Christmas, M, Breed, MF, Guerin, GR, Caddy-Retalic, S, McDonald, J, Jardine, DI, Leitch, EJ, Gellie, N, Hill, K, McCallum, K, Lowe, AJ (2017). Leaf trait associations with environmental variation in the wide-ranging shrub Dodonaea viscosa subsp. angustissima (Sapindaceae). Austral Ecology 43(5): 553-561. (doi:10.1111/aec.12474) |
| Hawaii native and non indigenous species. Traits and environment | Baruch, Z. & Goldstein, G. 1999. Leaf construction cost, nutrient concentration, and net CO2 assimilation of native and invasive species in Hawaii. Oecologia 121: 183-192 |
| Bauerle Vcmax and Jmax data | Bauerle, W.L., R. Oren, D.A. Way, S.S. Qian, P.C. Stoy, P.E. Thornton, J.D. Bowden, F.M. Hoffman, and R.F. Reynolds. 2012. Photoperiodic regulation of the seasonal pattern of photosynthetic capacity and the implications for carbon cycling. Proceedings of the National Academy of Sciences of the United States of America, 109:8612-8617 |
| Plant height of Mediterranean herb layer communities, Sardinia, Italy | Bazzato et al., in prep |
| Siberian shrub allometry | Berner, L. T., H. D. Alexander, M. M. Loranty, P. Ganzlin, M. C. Mack, S. P. Davydov, and S. J. Goetz (2015), Biomass allometry for alder, dwarf birch, and willow in boreal forest and tundra ecosystems of far northeastern Siberia and north-central Alaska, Forest Ecology and Management, 337, 110-118 |
| Tundra Trait Team | Bjorkman, AD, IH Myers-Smith, SC Elmendorf, S Normand, HJD Thomas, and 86 others (2018) Tundra Trait Team: a database of plant traits spanning the tundra biome. Global Ecology and Biogeography 27(12): 1402-1411. |
| ECOQUA South American Plant Traits Database | Blanco, C. C., E. E. Sosinski, B. R. C. dos Santos, M. A. da Silva, and V. D. Pillar. 2007. On the overlap between effect and response plant functional types linked to grazing. Community Ecology 8:57-65. |
| Fossil Leaf Traits | Blonder B, Royer DL, Johnson KR, Miller I, Enquist BJ (2014) Plant Ecological Strategies Shift Across the Cretaceous–Paleogene Boundary. PLOS Biology 12(9): e1001949. doi: 10.1371/journal.pbio.1001949 |
| Mt Baldy whole plant traits | Blonder, B, Kapas, RE, Dalton, RM, Graae, BJ, Heiling, JM, Opedal, ØH. Microenvironment and functional‐trait context dependence predict alpine plant community dynamics. J Ecol. 2018; 106: 1323– 1337. https://doi.org/10.1111/1365-2745.12973 |
| Leaf functional traits in the Hawaiian silversword alliance | Blonder, B., Baldwin, B., Enquist, B.J., Robichaux, R.H. (2016) Variation and macroevolution in leaf functional traits in the Hawaiian silversword alliance (Asteraceae). Journal of Ecology 104:219–228 DOI: 10.1111/1365-2745.12497 |
| Leaf Structure, Venation and Economic Spectrum | Blonder, B., Buzzard, B., Sloat, L., Simova, I., Lipson, R., Boyle, B., Enquist, B. (2012) The shrinkage effect biases estimates of paleoclimate. American Journal of Botany. 99.11 1756-1763 |
| Leaf Structure, Venation and Economic Spectrum | Blonder, B., Vasseur, F., Violle, C., Shipley, B., Enquist, B., Vile, D. Arabidopsis thaliana rejects theories for the origin of the leaf economics spectrum. (in review, New Phytologist) |
| Leaf Structure, Venation and Economic Spectrum | Blonder, B., Violle, C. and Enquist, B. J. (2013) Assessing the causes and scales of the leaf economics spectrum using venation networks in Populus tremuloides. Journal of Ecology 101: 981–989. doi:10.1111/1365-2745.12102 |
| Leaf Structure, Venation and Economic Spectrum | Blonder, B., Violle, C., Patrick, L., Enquist, B. Leaf venation networks and the origin of the leaf economics spectrum. Ecology Letters, 2011 |
| Plant Trait Database from Bajo Calima Region (Buenaventura, Colombia) | Bocanegra-Gonzáles KT, F Fernanadez-Mendez and JD Galvis-Jiménez (2015) Grupos funcionales de árboles en bosques secundarios de la región Bajo Calima (Buenaventura, Colombia). Boletin Cientitico de Museo de Historia Natural Uniersidad de Caldas, 19 (1): 17-40 DOI: 10.17151/bccm.2015.19.1.2 |
| Plant Traits of Canadian Forests | Bond-Lamberty, B., C. Wang, and S. T. Gower (2002), Above- and belowground biomass and sapwood area allometric equations for six boreal tree species of northern Manitoba, Can. J. For. Res., 32(8), 1441-1450. |
| Plant Traits of Canadian Forests | Bond-Lamberty, B., C. Wang, and S. T. Gower (2002), Leaf area dynamics of a boreal black spruce fire chronosequence, Tree Physiol., 22(14), 993-1001. |
| Plant Traits of Canadian Forests | Bond-Lamberty, B., C. Wang, and S. T. Gower (2003), The use of multiple measurement techniques to refine estimates of conifer needle geometry, Can. J. For. Res., 33(1), 101-105. |
| Plant Traits of Canadian Forests | Bond-Lamberty, B., C. Wang, and S.T. Gower (2004), Net primary production and net ecosystem production of a boreal black spruce fire chronosequence, Global Change Biol., 10(4), 473-487. |
| Tropical tree seedling above and below ground traits | Boonman, C.C.F., van Langevelde, F., Oliveras, I., Couédon, J., Luijken, N., Martini, D., and Veenendaal, E.M. (2019), On the importance of root traits in seedlings of tropical tree species. New Phytol. Accepted Author Manuscript |
| La Selva FT Data | Boukili, V. K., and R. L. Chazdon. 2017. Environmental filtering, local site factors and landscape context drive changes in functional trait composition during tropical forest succession. Perspectives in Plant Ecology, Evolution and Systematics 24 37–47 |
| Italian Alps Plant Traits Database | Bragazza L (2009) Conservation priority of Italian alpine habitats: a floristic approach based on potential distribution of vascular plant species. Biodiversity and Conservation 18: 2823–2835. |
| Xylem Functional Traits (XFT) Database: Nature Subset | Brendan Choat, Steven Jansen et al. (2012) Global convergence in the vulnerability of forests to drought. Nature 491, 752–755 doi:10.1038/nature11688 |
| Xylem Functional Traits (XFT) Database | Brendan Choat, Steven Jansen et al. (2012) Global convergence in the vulnerability of forests to drought. Nature 491, 752–755 doi:10.1038/nature11688 |
| Xylem Functional Traits (XFT) Database | Brendan Choat, Steven Jansen, Tim J. Brodribb, Herve Cochard, Sylvain Delzon, Radika Bhaskar, Sandra J. Bucci, Taylor S. Feild, Sean M. Gleason, Uwe G. Hacke, Anna L. Jacobsen, Frederic Lens, Hafiz Maherali, Jordi Martinez-Vilalta, Stefan Mayr, Maurizio Mencuccini, Patrick J. Mitchell, Andrea Nardini, Jarmila Pittermann, R. Brandon Pratt, John S. Sperry, Mark Westoby, Ian J. Wright & Amy E. Zanne (2012) Global convergence in the vulnerability of forests to drought. Nature 491:752-755 doi:10.1038/nature11688 |
| Traits of 48 native and alien Asteraceae in Germany (common-garden experiment) | Brendel, MR, Schurr, FM, Sheppard, CS. (2021) Inter- and intraspecific selection in alien plants: How population growth, functional traits and climate responses change with residence time. Global Ecol Biogeogr. Https://doi.org/10.1111/geb.13228 |
| Leaf-Height-Seed traits of Mediterranean mountain grassland | Bricca, A., Conti, L., Tardella, M. F., Catorci, A., Iocchi, M., Theurillat, J. P., & Cutini, M. (2019). Community assembly processes along a sub-Mediterranean elevation gradient: analyzing the interdependence of trait community weighted mean and functional diversity. Plant Ecology, 220(12), 1139-1151 |
| BiolFlor Database | Briemle, G., Nitsche, S. & Nitsche, L. (2002): Nutzungswertzahlen für Gefäßpflanzen des Grünlandes. In: Klotz, S., Kühn, I. & Durka, W. [eds.]: BIOLFLOR - Eine Datenbank zu biologisch-ökologischen Merkmalen der Gefäßpflanzen in Deutschland. -Schriftenreihe für Vegetationskunde 38. Bundesamt für Naturschutz, Bonn. |
| ParanaTrees2015 | Brumnich, F., Marchetti, Z. Y., & Pereira, M. S. (2019). Changes in forest diversity over a chronosequence of fluvial islands. iForest - Biogeosciences and Forestry, 12, 306-316 |
| Dataset on reproductive traits of Scandinavian alpine plants | Bruun, H. H. (2019) Dataset on reproductive traits of Scandinavian alpine plants. Data in Brief (accepted, DIB-D-19-01007R2) |
| Atractocarpus from new Caledonia | Bruy D, Hattermann T, Barrabé L, Mouly A, Barthélémy D, Isnard S. 2018. Evolution of Plant Architecture, Functional Diversification and Divergent Evolution in the Genus Atractocarpus (Rubiaceae) for New Caledonia. Frontiers in Plant Science 9 |
| Garmisch-Partenkirchen elevational gradients | Bucher, S.F., Auerswald, K., Grün-Wenzel, C., Higgins, S.I., Jorge, J.G. and Römermann, C. 2017: Stomatal traits relate to habitat preferences of herbaceous species in a temperate climate. FLORA - Morphology, Distribution, Functional Ecology of Plants 229, 107-115. |
| Garmisch-Partenkirchen elevational gradients | Bucher, S.F., Auerswald, K., Tautenhahn, S., Geiger, A., Otto, J., Müller, A. and Römermann, C. (2016) Intra- and interspecific variation in stomatal pore area index along altitudinal gradients and its relation to leaf functional traits. Plant Ecology 217, 229-240 |
| Garmisch-Partenkirchen elevational gradients | Bucher, S.F., König, P., Migliavacca, M., Menzel, A., Ewald J. and Römermann, C. 2018: Traits and climate are associated with first flowering day in herbaceous species along elevational gradients. Ecology and Evolution, in Press. Https://doi.org/10.1002/ece3.3720. |
| Arable weed trait data set | Buerger J, Malyshev AV, Colbach N (2020) Populations of arable weed species show intraspecific variability in germination base temperature but not in early growth rate. PLoS ONE 15(10): e0240538. https://doi.org/10.1371/journal.pone.0240538 |
| Plant Traits from Circeo National Park, Italy | Burrascano S, Copiz R, Del Vico E, Fagiani S, Giarrizzo E, Mei M, Mortelliti A, Sabatini FM, Blasi C (2015) Wild boar rooting intensity determines shifts in understorey composition and functional traits. COMMUNITY ECOLOGY 16(2) 244-253 DOI: 10.1556/168.2015.16.2.12 |
| Traits of US Desert Woody Plant Species | Butterfield, B.J. and J.M. Briggs. 2011. Regeneration niche differentiates functional strategies of desert woody plant species. Oecologia, 165:477-487. |
| Cadotte 2017 Ecology Letters: herbaceous traits measured in the filed | Cadotte, M. W. 2017. Functional traits explain ecosystem function through opposing mechanisms. Ecology Letters 20:989-996 |
| Ecophysiology of Selaginella and fern species in a Costa Rica wet tropical forest floor | Campany CE, L Martin, and JE Watkins. Convergence of ecophysiological traits drives floristic composition of early lineage vascular plants in a tropical forest floor 2018. Annals of Botany |
| Plant Physiology Database | Campbell, C., L. Atkinson, J. Zaragoza-Castells, M. Lundmark, O. Atkin, and V. Hurry. 2007. Acclimation of photosynthesis and respiration is asynchronous in response to changes in temperature regardless of plant functional group. New Phytologist 176:375-389. |
| Sardinia_elevation gradient | Campetella G., Chelli S., Wellstein C., Farris E., Calvia G., Simonetti E., Borsukiewicz L., Vanderplank S., Marignani M. (2019). Contrasting patterns in leaf traits of Mediterranean shrub communities along an elevation gradient: measurements matter. Plant Ecology, https://doi.org/10.1007/s11258-019-00951-y |
| Leaf Traits in Central Apennines Beech Forests | Campetella, G; Botta-Dukát, Z; Wellstein, C; Canullo, R; Gatto, S; Chelli, S; Mucina, L; Bartha, S (2011): Patterns of plant trait-environment relationships along a forest succession chronosequence. Agriculture, Ecosystems & Environment, 145(1), 38-48. doi:10.1016/j.agee.2011.06.025 |
| Leaf Traits in Italian Central Apennines Beech Forests | Campetella, G; Botta-Dukát, Z; Wellstein, C; Canullo, R; Gatto, S; Chelli, S; Mucina, L; Bartha, S (2011): Patterns of plant trait-environment relationships along a forest succession chronosequence. Agriculture, Ecosystems & Environment, 145(1), 38-48. doi:10.1016/j.agee.2011.06.025 |
| Photosynthetic Capacity Dataset | Carswell, F. E., Meir, P., Wandelli, E. V., Bonates, L. C. M., Kruijt, B., Barbosa, E. M., Nobre, A. D. & Jarvis, P. G. 2000 Photosynthetic capacity in a central Amazonian rain forest. Tree physiology. 20, 3, p. 179-186 8 p. |
| Fabio Carvalho lowland fen peatland | Carvalho F, Brown KA, Waller MP, Bunting MJ, Boom A, Leng MJ (2019) A method for reconstructing temporal changes in vegetation functional trait composition using Holocene pollen assemblages. PLoS ONE 14(5): e0216698. https://doi.org/10.1371/journal.pone.0216698 |
| Fabio Carvalho lowland fen peatland | Carvalho, F, Brown, KA, Waller, MP, Boom, A (2019) Leaf traits interact with management and water table to modulate ecosystem properties in fen peatlands. Plant and Soil. DOI: https://doi.org/10.1007/s11104-019-04126-6 https://doi.org/10.1007/s11104-019-04126-6 |
| Sheffield & Spain Woody Database | Castro-Diez, P., J. P. Puyravaud, and J. H. C. Cornelissen. 2000. Leaf structure and anatomy as related to leaf mass per area variation in seedlings of a wide range of woody plant species and types. Oecologia 124:476-486. |
| Sheffield & Spain Woody Database | Castro-Diez, P., J. P. Puyravaud, J. H. C. Cornelissen, and P. Villar-Salvador. 1998. Stem anatomy and relative growth rate in seedlings of a wide range of woody plant species and types. Oecologia 116:57-66. |
| Traits related to riparian plant invasion in South East Australia | Catford, J. A., Morris, W. K., Vesk, P. A., Gippel, C. J. & Downes, B. J. (2014) Species and environmental characteristics point to flow regulation and drought as drivers of riparian plant invasion. Diversity and Distributions, 20, 1084–1096. http://dx.doi.org/10.1111/ddi.12225 |
| Floridian Leaf Traits Database | Cavender-Bares, J., A. Keen, and B. Miles. 2006. Phylogenetic structure of floridian plant communities depends on taxonomic and spatial scale. Ecology 87:S109-S122. |
| Leaf and Whole-Plant Traits Database: Hydraulic and Gas Exchange Physiology, Anatomy, Venation Struc | Cavender-Bares, J., L. Sack, and J. Savage. 2007. Atmospheric and soil drought reduce nocturnal conductance in live oaks. Tree Physiology 27:611-620. |
| Malga San Simone Trait Database (MSS) | Cerabolini B., Pierce S., Luzzaro A., Ossola A. (2010) Species evenness affects ecosystem processes in situ via diversity in the adaptive strategies of dominant species. Plant Ecology, 207(2): 333-345 |
| Flora d’Italia Functional Traits Hoard (FIFTH) | Cerabolini B.E.L., Brusa G., Ceriani R.M., De Andreis R., Luzzaro A. & Pierce S. 2010. Can CSR classification be generally applied outside Britain? Plant Ecology 210: 253-261 |
| Functional Traits of Trees in Golfo Dulce, Costa Rica | Chacón-Madrigal, E., Wanek, W., Hietz, P., & S. Dullinger. 2018. Traits indicating a conservative resource strategy are weakly related to narrow range size in a group of neotropical trees. Perspectives in Plant Ecology, Evolution, and Systematics, https://doi.org/10.1016/j.ppees.2018.01.003 |
| Functional Traits of Trees in Golfo Dulce, Costa Rica | Chacón-Madrigal, E., Wolfgang, W., Hietz, P. & Dullinger, S. A stress-tolerance syndrome distinguishes endemic tropical trees from their widespread congeners. Unpublished |
| Temperate tree species in New Jersey USA | Chen et al. (2014) Species-Independent Down-Regulation of Leaf Photosynthesis and Respiration in Response to Shading: Evidence from Six Temperate Tree Species. PLoS ONE 9(4): e91798. https://doi.org/10.1371/journal.pone.0091798 |
| Fruit type, fruit dimension and flowering time | Chen S-C, W Cornwell, H-X Zhang & AT Moles. (2017) Plants show more flesh in the tropics: variation in fruit type along latitudinal and climatic gradients. Ecography 40(4): 531-538 |
| Growth form data for 3581 Australian species | Chen S-C, W Cornwell, H-X Zhang & AT Moles. (2017) Plants show more flesh in the tropics: variation in fruit type along latitudinal and climatic gradients. Ecography 40(4): 531-538 |
| Leaf inclination angle | Chianucci, F., Pisek, J., Raabe, K., Marchino, L., Ferrara, C. and Corona, P., 2018. A dataset of leaf inclination angles for temperate and boreal broadleaf woody species. Annals of Forest Science, 75(2), p.50. doi: 10.1007/s13595-018-0730-x |
| Leaf and Whole-Plant Traits Database: Hydraulic and Gas Exchange Physiology, Anatomy, Venation Struc | Choat, B., L. Sack, and N. M. Holbrook. 2007. Diversity of hydraulic traits in nine Cordia species growing in tropical forests with contrasting precipitation. New Phytologist 175:686-698. |
| 1000Seedweight | Chrobock T, Kempel A, Fischer M, van Kleunen M (2011) Introduction bias: cultivated plant species germinate faster and more profusely than native species in Switzerland. Basic and Applied Ecology 12:244-250 |
| Mediterranean psammophytes | Ciccarelli D. (2015) - Mediterranean coastal dune vegetation: Are disturbance and stress the key selective forces that drive the psammophilous succession? Estuarine, Coastal and Shelf Science 165(5):247–253 doi: 10.1016/j.ecss.2015.05.023 |
| Plant Traits from Romania | Ciocarlan V. (2009). The illustrated Flora of Romania. Pteridophyta et Spermatopyta. Editura Ceres, 1141 p (in Romanian). |
| Leaf and Whole-Plant Traits Database: Hydraulic and Gas Exchange Physiology, Anatomy, Venation Struc | Coomes, D. A., S. Heathcote, E. R. Godfrey, J. J. Shepherd, and L. Sack. 2008. Scaling of xylem vessels and veins within the leaves of oak species. Biology Letters 4:302-306. |
| Sheffield Database | Cornelissen, J. H. C. 1996. An experimental comparison of leaf decomposition rates in a wide range of temperate plant species and types. Journal of Ecology 84:573-582. |
| Sheffield Database | Cornelissen, J. H. C., B. Cerabolini, P. Castro-Diez, P. Villar-Salvador, G. Montserrat-Marti, J. P. Puyravaud, M. Maestro, M. J. A. Werger, and R. Aerts. 2003. Functional traits of woody plants: correspondence of species rankings between field adults and laboratory-grown seedlings? Journal of Vegetation Science 14:311-322. |
| Sheffield & Spain Woody Database | Cornelissen, J. H. C., B. Cerabolini, P. Castro-Diez, P. Villar-Salvador, G. Montserrat-Marti, J. P. Puyravaud, M. Maestro, M. J. A. Werger, and R. Aerts. 2003. Functional traits of woody plants: correspondence of species rankings between field adults and laboratory-grown seedlings? Journal of Vegetation Science 14:311-322. |
| Abisko & Sheffield Database | Cornelissen, J. H. C., H. M. Quested, D. Gwynn-Jones, R. S. P. Van Logtestijn, M. A. H. De Beus, A. Kondratchuk, T. V. Callaghan, and R. Aerts. 2004. Leaf digestibility and litter decomposability are related in a wide range of subarctic plant species and types. Functional Ecology 18:779-786. |
| Abisko & Sheffield Database | Cornelissen, J. H. C., M. J. A. Werger, P. CastroDiez, J. W. A. vanRheenen, and A. P. Rowland. 1997. Foliar nutrients in relation to growth, allocation and leaf traits in seedlings of a wide range of woody plant species and types. Oecologia 111:460-469. |
| Sheffield Database | Cornelissen, J. H. C., N. Perez-Harguindeguy, S. Diaz, J. P. Grime, B. Marzano, M. Cabido, F. Vendramini, and B. Cerabolini. 1999. Leaf structure and defence control litter decomposition rate across species and life forms in regional floras on two continents. New Phytologist 143:191-200. |
| Abisko & Sheffield Database | Cornelissen, J. H. C., P. C. Diez, and R. Hunt. 1996. Seedling growth, allocation and leaf attributes in a wide range of woody plant species and types. Journal of Ecology 84:755-765. |
| Sheffield Database | Cornelissen, J. H. C., P. C. Diez, and R. Hunt. 1996. Seedling growth, allocation and leaf attributes in a wide range of woody plant species and types. Journal of Ecology 84:755-765. |
| Sheffield Database | Cornelissen, J. H. C., R. Aerts, B. Cerabolini, M. J. A. Werger, and M. G. A. van der Heijden. 2001. Carbon cycling traits of plant species are linked with mycorrhizal strategy. Oecologia 129:611-619. |
| Sheffield & Spain Woody Database | Cornelissen, J.H.C. 1999. A triangular relationship between leaf size and seed size among woody species: allometry, ontogeny, ecology and taxonomy. Oecologia 118: 248-255. |
| A Global Dataset of Leaf Delta13C Data | Cornwell WK, Wright I, Turner J, Maire V, Barbour M, Cernusak L, Dawson T, Ellsworth D, Farquhar G, Griffiths H, Keitel C, Knohl A, Reich P, Williams D, Bhaskar R, Cornelissen JHC, Richards A, Schmidt S, Valladares F, Körner C, Schulze E, Buchmann N, Santiago L. 2017. A global dataset of leaf D13 C values. 10.5281/zenodo.569501 |
| Jasper Ridge Californian Woody Plants Database | Cornwell, W. K. and D. D. Ackerly. 2009. Community assembly and shifts in plant trait distributions across an environmental gradient in coastal California. Ecological Monographs 79:109-126. |
| Jasper Ridge Californian Woody Plants Database | Cornwell, W. K., D. W. Schwilk, and D. D. Ackerly. 2006. A trait-based test for habitat filtering: Convex hull volume. Ecology 87:1465-1471. |
| ArtDeco Database | Cornwell, W. K., J. H. C. Cornelissen, K. Amatangelo, E. Dorrepaal, V. T. Eviner, O. Godoy, S. E. Hobbie, B. Hoorens, H. Kurokawa, N. Pérez-Harguindeguy, H. M. Quested, L. S. Santiago, D. A. Wardle, I. J. Wright, R. Aerts, S. D. Allison, P. van Bodegom, V. Brovkin, A. Chatain, T. V. Callaghan, S. Díaz, E. Garnier, D. E. Gurvich, E. Kazakou, J. A. Klein, J. Read, P. B. Reich, N. A. Soudzilovskaia, M. V. Vaieretti, and M. Westoby. 2008. Plant species traits are the predominant control on litter decomposition rates within biomes worldwide. Ecology Letters 11:1065-1071. |
| Leaf and Whole-Plant Traits Database: Hydraulic and Gas Exchange Physiology, Anatomy, Venation Struc | Cornwell, W. K., R. Bhaskar, L. Sack, S. Cordell, and C. K. Lunch. 2007. Adjustment of structure and function of Hawaiian Metrosideros polymorpha at high vs. low precipitation. Functional Ecology 21:1063-1071. |
| Global Woodiness Database | Cornwell, W.K., FitzJohn, R., Stevens, P.F., Calaminus, A., Soltis, D.E., Soltis, P.S., Govaerts, R., Wright, I.J., Oleksyn, J., Reich, P.B., Royer, D.L., Aarssen, L., Hemmings, F., Leishman, M., Moles, A.T., Swenson, N.G., Warman, L., Bertin, R.I., Ordonez, A.,Zanne, A.E. Global woodiness database. Data from: Three keys to the radiation of angiosperms into freezing environments. Nature. Dryad Digital Repository.http://dx.doi.org/10.5061/dryad.63q27/2. (2013). |
| Plant Traits for Grassland Species (Konza Prairie, Kansas, USA) | Craine JM, Nippert JB, Towne EG, Tucker S, Kembel SW, Skibbe A, McLauchlan KK (2011) Functional consequences of climate-change induced plant species loss in a tallgrass prairie. Oecologia 165: 1109-1117 |
| Plant Traits for Grassland Species (Konza Prairie, Kansas, USA) | Craine JM, Ocheltree TW, Nippert JB, Towne EG, Skibbe AM, Kembel SW, Fargione JE (2012) Global diversity of drought tolerance and grassland climate-change resilience. Nature Climate Change |
| Plant Traits for Grassland Species (Konza Prairie, Kansas, USA) | Craine JM, Towne EG, Ocheltree TW, Nippert JB (2012) Community traitscape of foliar nitrogen isotopes reveals N availability patterns in a tallgrass prairie. Plant Soil 356: 395-403 |
| Global 15N Database | Craine, J. M., A. J. Elmore, M. P. M. Aidar, M. Bustamante, T. E. Dawson, E. A. Hobbie, A. Kahmen, M. C. Mack, K. K. McLauchlan, A. Michelsen, G. B. Nardoto, L. H. Pardo, J. Penuelas, P. B. Reich, E. A. G. Schuur, W. D. Stock, P. H. Templer, R. A. Virginia, J. M. Welker, and I. J. Wright. 2009. Global patterns of foliar nitrogen isotopes and their relationships with climate, mycorrhizal fungi, foliar nutrient concentrations, and nitrogen availability. New Phytologist 183:980-992. |
| Roots Of the World (ROW) Database | Craine, J. M., W. G. Lee, W. J. Bond, R. J. Williams, and L. C. Johnson. 2005. Environmental constraints on a global relationship among leaf and root traits of grasses. Ecology 86:12-19. |
| Panama Tree Traits | Craven, D., D. Braden, M. S. Ashton, G. P. Berlyn, M. Wishnie, and D. Dent. 2007. Between and within-site comparisons of structural and physiological characteristics and foliar nutrient content of 14 tree species at a wet, fertile site and a dry, infertile site in Panama. Forest Ecology and Management 238:335-346. |
| Panama Tree Traits | Craven, D., D. Dent, D. Braden, M. S. Ashton, G. P. Berlyn, and J. S. Hall. 2011. Seasonal variability of photosynthetic characteristics influences growth of eight tropical tree species at two sites with contrasting precipitation in Panama. Forest Ecology and Management 261:1643-1653. |
| mixed_US_rangeland_size_biomass | Cunliffe, A., K. Anderson, F. Boschetti, R. Brazier, H. Graham, I. Myers-Smith, T. Astor, M. Boer, L. Calvo, P. Clark, M. Cramer, M. Encinas-Lara, S. Escarzaga, J. Fernández-Guisuraga, A. Fisher, K. Gdulová, B. Gillespie, A. Griebel, N. Hanan, M. Hanggito, S. Haselberger, C. Havrilla, P. Heilman, W. Ji, J. Karl, M. Kirchhoff, S. Kraushaar, M. Lyons, I. Marzolff, M. Mauritz, C. McIntire, D. Metzen, L. Méndez-Barroso, S. Power, J. Prošek, E. Sanz-Ablanedo, K. Sauer, D. Schulze-Brüninghoff, P. Šímová, S. Sitch, J. Smit, C. Steele, S. Suárez-Seoane, S. Vargas, M. Villarreal, F. Visser, Wachendorf, H. Wirnsberger, and R. Wojcikiewicz (In Review) Global application of a drone photogrammetry protocol for predicting aboveground biomass in non-forest ecosystems. Preprint on bioRxiv, DOI:10.1101/2020.07.16.206011 Cunliffe, A., et al., (2020). Allometric modelling of plant biomass from drone-acquired photographs: drone images, ground control marker coordinates and biomass data from 36 sites, 2016-2020. NERC EIDC. https://doi.org/10.5285/1ec13364-cbc6-4ab5-a147-45a103853424 |
| Juniperus Monosperma allometric traits for biomass and sapwood area | Cunliffe, Andrew M., Cameron D. McIntire, Fabio Boschetti, Katherine J. Sauer, Marcy Litvak, Karen Anderson, and Richard E. Brazier (2021) Allometric Relationships for Predicting Aboveground Biomass and Sapwood Area of Oneseed Juniper (Juniperus Monosperma) Trees. Frontiers in Plant Science 11, no. 94: 1–12. https://doi.org/10.3389/fpls.2020.00094. Cunliffe, A., C. McIntire, F. Boschetti, K. Sauer, M. Litvak, R. Brazier, K. Anderson (2019). Field measurements of aboveground biomass, canopy area, stem diameter and sapwood area of Juniperus monosperma trees in New Mexico, collected in 2018 and 2019. NERC EIDC. Https://doi.org/10.5285/871443a9-6634-4eba-abb5-286a1ab58e9b |
| Salix richardsonii biomass, canopy height, carbon and nitrogen content | Cunliffe, Andrew M., Jakob Assmann, Gergana Daskalova, Jeffrey T. Kerby, and Isla H. Myers-Smith (2020) Aboveground Biomass Corresponds Strongly with Drone-Derived Canopy Height but Weakly with Greenness (NDVI) in a Shrub Tundra Landscape. Environmental Research Letters, 2020. https://doi.org/10.1088/1748-9326/aba470. Cunliffe, A., J. Assmann, J. Kerby and I. Myers-Smith. (2020). Canopy height, spectral reflectance (NDVI), and aboveground biomass of Salix richardsonii across a wet graminoid-shrubland ecotone on Qikiqtaruk – Herschel Island, Yukon, Canada (2016). NERC PDC. Https://doi.org/10.5285/61C5097B-6717-4692-A8A4-D32CCA0E61A9 |
| Italian Alps Plant Traits Database | Dainese M, Bragazza L (2012) Plant traits across different habitats of the Italian Alps: a comparative analysis between native and alien species. Alpine Botany 122: 11-21. |
| Leaf traits Rhizophora apiculata in Can Gio - Vietnam | Dang-Le Anh Tuan. Characterization growth of Rhizophora apiculata Blume at Can Gio Mangrove Biosphere Reserve, Vietnam. Unpublished data |
| Leaf traits (and a few seed weights) collected from plants in the Macquarie Marshes, Australia | Dawson, S. K., Warton, D. I., Kingsford, R. T., Berney, P. , Keith, D. A., Catford, J. A. and Mori, A. (2017), Plant traits of propagule banks and standing vegetation reveal flooding alleviates impacts of agriculture on wetland restoration. J Appl Ecol, 54: 1907-1918. doi:10.1111/1365-2664.12922 |
| Traits from Semi-Arid Mediterranean Ecosystems | de Frutos A, Teresa Navarro, Yolanda Pueyo, Concepción L. Alados (2015) Inferring Resilience to Fragmentation-Induced Changes in Plant Communities in a Semi-Arid Mediterranean Ecosystem DOI: 10.1371/journal.pone.0118837 |
| Cabo de Gata-Níjar Natural Park | De Frutos, Á., Navarro, T., Pueyo, Y., Alados, C.L., 2015. Inferring Resilience to Fragmentation-Induced Changes in Plant Communities in a Semi-Arid Mediterranean Ecosystem. PLoS ONE 10, e0118837. doi:10.1371/journal.pone.0118837 |
| JACARE A-Ci leaf trait data base 2017 | De Kauwe, MG, Lin, Y-S, Wright, IJ, Medlyn, BE, Crous, KY, Ellsworth, DS, Maire, V, Prentice, IC, Atkin, OK, Rogers, A, Niinemets, Ü, Serbin, SP, Meir, P, Uddling, J, Togashi, HF, Tarvainen, L, Weerasinghe, LK, Evans, BJ, Ishida, FY, Domingues, TF (2016) A test of the ‘one-point method’ for estimating maximum carboxylation capacity from field-measured, light-saturated photosynthesis. New Phytologist 210, 1130-1144. |
| Colt Park Mesocosms | De Long J, Jackson B, Wilkinson A, Pritchard W, Oakley S, Mason K, Stephan J, Ostle N, Johnson D, Baggs E, Bardgett R (2019) Data from: Relationships between plant traits, soil properties and carbon fluxes differ between monocultures and mixed communities in temperate grassland. Dryad Digital Repository. https://doi.org/10.5061/dryad.gh41n3j |
| Colt Park Mesocosms | De Long, JR, Jackson, BG, Wilkinson, A, et al. Relationships between plant traits, soil properties and carbon fluxes differ between monocultures and mixed communities in temperate grassland. J Ecol. 2019; 107: 1704– 1719. https://doi.org/10.1111/1365-2745.13160 |
| Leaf N-Retention Database | De Vries F., Bardgett R.D. (2016) Plant community controls on short-term ecosystem nitrogen retention. New Phytologist. doi: 10.1111/nph.13832 |
| Arboretum Grosspoessna 2014 leaf chemical and photosynthesis traits | Dechant, B., Cuntz, M., Vohland, M., Schulz, E., & Doktor, D. (2017). Estimation of photosynthesis traits from leaf reflectance spectra: Correlation to nitrogen content as the dominant mechanism. Remote Sensing of Environment, 196, 279–292. https://doi.org/10.1016/j.rse.2017.05.019 |
| Barbeau Leaf Minerals, Quercus petraea, Carpinus betulus | Delpierre, N., Berveiller, D., Granda, E., & Dufrêne, E. (2016). Wood phenology, not carbon input, controls the interannual variability of wood growth in a temperate oak forest. New Phytologist, 210(2), 459-470 |
| Traits of Hemiparastic Plants | Demey A, J Staelens, L Baeten, P Boeckx, M Hermy, J Kattge, K Verheyen (2013) Nutrient input from hemiparasitic litter favors plant species with a fast-growth strategy. Plant and Soil DOI 10.1007/s11104-013-1658-4 |
| Sheffield Database | Díaz, S., J. G. Hodgson, K. Thompson, M. Cabido, J. H. C. Cornelissen, A. Jalili, G. Montserrat-Martí, J. P. Grime, F. Zarrinkamar, Y. Asri, S. R. Band, S. Basconcelo, P. Castro-Díez, G. Funes, B. Hamzehee, M. Khoshnevi, N. Pérez-Harguindeguy, M. C. Pérez-Rontomé, F. A. Shirvany, F. Vendramini, S. Yazdani, R. Abbas-Azimi, A. Bogaard, S. Boustani, M. Charles, M. Dehghan, L. de Torres-Espuny, V. Falczuk, J. Guerrero-Campo, A. Hynd, G. Jones, E. Kowsary, F. Kazemi-Saeed, M. Maestro-Martínez, A. Romo-Díez, S. Shaw, B. Siavash, P. Villar-Salvador, and M. R. Zak. 2004. The plant traits that drive ecosystems: Evidence from three continents. Journal of Vegetation Science 15:295-304. |
| Sheffield-Iran-Spain Database | Díaz, S., J. G. Hodgson, K. Thompson, M. Cabido, J. H. C. Cornelissen, A. Jalili, G. Montserrat-Martí, J. P. Grime, F. Zarrinkamar, Y. Asri, S. R. Band, S. Basconcelo, P. Castro-Díez, G. Funes, B. Hamzehee, M. Khoshnevi, N. Pérez-Harguindeguy, M. C. Pérez-Rontomé, F. A. Shirvany, F. Vendramini, S. Yazdani, R. Abbas-Azimi, A. Bogaard, S. Boustani, M. Charles, M. Dehghan, L. de Torres-Espuny, V. Falczuk, J. Guerrero-Campo, A. Hynd, G. Jones, E. Kowsary, F. Kazemi-Saeed, M. Maestro-Martínez, A. Romo-Díez, S. Shaw, B. Siavash, P. Villar-Salvador, and M. R. Zak. 2004. The plant traits that drive ecosystems: Evidence from three continents. Journal of Vegetation Science 15:295-304. |
| Mont_Mégantic_Individual_Traits_2016-2017 | DOI: 10.5281/zenodo.3258342 |
| LBA ECO Tapajos: Leaf Characteristics and Photosynthesis | Domingues , T.F., Berry, J.A., Martinelli, L.A., Ometto, J.P., Ehlehringer, J.R. (2005) Parameterization of Canopy Structure and Leaf-Level Gas Exchange for an Eastern Amazonian Tropical Rain Forest (Tapajós National Forest, Pará, Brazil). Earth Interactions 9, 1. |
| TROBIT West Africa | Domingues TF, Meir P, Feldpausch TR, et al. (2010) Co-limitation of photosynthetic capacity by nitrogen and phosphorus in West Africa woodlands. Plant, Cell & Environment (33): 959-980. |
| TERN_ATN traits database | Dong N, Iain Colin Prentice, Ian J. Wright, Brad J. Evans, Henrique Furstenau Togashi, Stefan Caddy-Retalic, Francesca A. McInerney, Ben Sparrow, Emrys Leitch and Andrew J. Lowe. Components of leaf-trait variation along environmental gradients. New Phytologist, VOLUME, PAGE NUMBERS |
| Ausplot_traits | Dong, N., Prentice, I. C., Evans, B. J., Caddy-Retalic, S., Lowe, A. J., & Wright, I. J. (2017). Leaf nitrogen from first principles: field evidence for adaptive variation with climate. Biogeosciences, 14(2), 481-495. doi:10.5194/bg-14-481-2017 |
| Ukraine Wetlands Plant Traits Database | Douma, J.C., Bardin, V., Bartholomeus, R.P. and van Bodegom, P.M. (2012), Quantifying the functional responses of vegetation to drought and oxygen stress in temperate ecosystems. Funct Ecol, 26: 1355-1365. doi:10.1111/j.1365-2435.2012.02054.x |
| Trait Data for African Plants - a Photo Guide | Dressler S, M Schmidt, G Zizka (2014) Introducing African Plants—A Photo Guide—An Interactive Photo Data-Base and Rapid Identification Tool for Continental Africa. Taxon 63(5) 1159-1161 DOI: http://dx.doi.org/10.12705/635.26 |
| Orchid Trait Dataset | Dressler, R. L. 2003. Orchidaceae. in B. E. Hammel, M. H. Grayum, C. Herrera, and N. Zamora, eds. Manual de Plantas de Costa Rica. Vol. III. Monocotiledoneas (Orchidaceae-Zingiberaceae). Monogr. Syst. Bot. Mo. Bot. Gard. 93:1–595. |
| ECOQUA South American Plant Traits Database | Duarte, L. D., M. B. Carlucci, S. M. Hartz, and V. D. Pillar. 2007. Plant dispersal strategies and the colonization of Araucaria forest patches in a grassland-forest mosaic. Journal of Vegetation Science 18:847-858. |
| Leaf and Whole-Plant Traits Database: Hydraulic and Gas Exchange Physiology, Anatomy, Venation Struc | Dunbar-Co, S., M. J. Sporck, and L. Sack. 2009. Leaf Trait Diversification and Design in Seven Rare Taxa of the Hawaiian Plantago Radiation. International Journal of Plant Sciences 170:61-75. |
| BiolFlor Database | Durka, W. (2002) Phylogenie der Farn- und Blütenpflanzen Deutschlands. - In: Klotz, S., Kühn, I. & Durka, W. [Hrsg.]: BIOLFLOR - Eine Datenbank zu biologisch-ökologischen Merkmalen der Gefäßpflanzen in Deutschland. - Schriftenreihe für Vegetationskunde 38: 75-91. Bundesamt für Naturschutz, Bonn. |
| BiolFlor Database | Durka, W. (2002): Blüten- und Reproduktionsbiologie. In: Klotz, S., Kühn, I. & Durka, W. [eds.]: BIOLFLOR - Eine Datenbank zu biologisch-ökologischen Merkmalen der Gefäßpflanzen in Deutschland. - Schriftenreihe für Vegetationskunde 38: 133-175. Bundesamt für Naturschutz, Bonn. |
| BiolFlor Database | Durka, W. (2002): Chromosomenzahlen, Ploidiestufen und DNA-Gehalte. In: Klotz, S., Kühn, I. & Durka, W. [eds.]: BIOLFLOR - Eine Datenbank zu biologisch-ökologischen Merkmalen der Gefäßpflanzen in Deutschland. - Schriftenreihe für Vegetationskunde 38. Bundesamt für Naturschutz, Bonn. |
| Specific leaf area responses to environmental gradients through space and time | Dwyer, J. M., R. J. Hobbs, and M. M. Mayfield. 2014. Specific leaf area responses to environmental gradients through space and time. Ecology 95:399-410 |
| Leaf trait records of rare and endangered plant species in the Pannonian flora | E-Vojtkó A, Balogh N, Deák B, Kelemen A, Kis Sz, Kiss R, Lovas-Kiss Á, Löki V, Lukács K, Molnár VA, Nagy T, Sonkoly J, Süveges K, Takács A, Tóth E, Tóth K, Tóthmérész B, Török P, Valkó O, Vojtkó A & Lukács BA: Leaf trait records of vascular plant species in the Pannonian flora with special focus on endemics and rarities Folia Geobotanica 55, 73–79 |
| eHALOPH - Halophytes Database (2015) | eHALOPH - Halophytes Database (version 3.11) Tim Flowers, Joaquim Santos, Moritz Jahns, Brian Warburton and Peter Reed; http://www.sussex.ac.uk/affiliates/halophytes, accessed 2015 |
| eHALOPH - Halophytes Database (2018) | eHALOPH - Halophytes Database (version 3.11) Tim Flowers, Joaquim Santos, Moritz Jahns, Brian Warburton and Peter Reed; http://www.sussex.ac.uk/affiliates/halophytes, accessed 2017 |
| Caucasus Plant Traits Database | Elumeeva, T.G., Onipchenko, V.G., Egorov, A.V. et al. Long-term vegetation dynamic in the Northwestern Caucasus: which communities are more affected by upward shifts of plant species?. Alp Botany 123, 77–85 (2013). https://doi.org/10.1007/s00035-013-0122-7 |
| Plant growth form dataset for the New World | Engemann, K., Sandel, B., Boyle, B., Enquist, B. J., … Svenning, J.-C. (2016), A plant growth form dataset for the New World. Ecology, 97: 3243. doi:10.1002/ecy.1569 |
| The DIRECT Plant Trait Database | Everwand G, Fry, EL, Eggers T, Manning P (2014) Seasonal variation in the relationship between plant traits and grassland carbon and water fluxes. Ecosystems 17, 1095-1108 |
| Seed Characteristics of Ericaceae | Fagúndez J & J. Izco 2008. Seed morphology of two distinct species of Erica L. (Ericaceae). Acta Botanica Malacitana 33: 1-9 |
| Seed Characteristics of Ericaceae | Fagúndez, J., Juan, R., Fernández, I., Pastor, J., & Izco, J. (2010). Systematic relevance of seed coat anatomy in the European heathers (Ericeae, Ericaceae). Plant systematics and evolution, 284(1-2), 65-76. |
| BAAD: a biomass and allometry database for woody plants | Falster DS, Remko A. Duursma, Masae I. Ishihara, Diego R. Barneche, Richard G. FitzJohn, Angelica Våhammar, Masahiro Aiba, Makoto Ando, Niels Anten, Michael J. Aspinwall, Jennifer L. Baltzer, Christopher Baraloto, Michael Battaglia, John J. Battles, Ben Bond-Lamberty, Michiel van Breugel, James Camac, Yves Claveau, Lluís Coll, Masako Dannoura, Sylvain Delagrange, Jean-Christophe Domec, Farrah Fatemi, Wang Feng, Veronica Gargaglione, Yoshiaki Goto, Akio Hagihara, Jefferson S. Hall, Steve Hamilton, Degi Harja, Tsutom Hiura, Robert Holdaway, Lindsay B. Hutley, Tomoaki Ichie, Eric J. Jokela, Anu Kantola, Jeff W. G. Kelly, Tanaka Kenzo, David King, Brian D. Kloeppel, Takashi Kohyama, Akira Komiyama, Jean-Paul Laclau, Christopher H. Lusk, Douglas A. Maguire, Guerric le Maire, Annikki Mäkelä, Lars Markesteijn, John Marshall, Katherine McCulloh, Itsuo Miyata, Karel Mokany, Shigeta Mori, Randall W. Myster, Masahiro Nagano, Shawna L. Naidu, Yann Nouvellon, Anthony P. OGrady, Kevin L. OHara, Toshiyuki Ohtsuka, Noriyuki Osada, Olusegun O. Osunkoya, Pablo Luis Peri, Any Mary Petritan, Lourens Poorter, Angelika Portsmuth, Catherine Potvin, Johannes Ransijn, Douglas Reid, Sabina C. Ribeiro, Scott D. Roberts, Rolando Rodríguez, Angela Saldaña-Acosta, Ignacio Santa-Regina, Kaichiro Sasa, N. Galia Selaya, Stephen C. Sillett, Frank Sterck, Kentaro Takagi, Takeshi Tange, Hiroyuki Tanouchi, David Tissue, Toru Umehara, Hajime Utsugi, Matthew A. Vadeboncoeur, Fernando Valladares, Petteri Vanninen, Jian R. Wang, Elizabeth Wenk, Richard Williams, Fabiano de Aquino Ximenes, Atsushi Yamaba, Toshihiro Yamada, Takuo Yamakura, Ruth D. Yanai, and Robert A. York. 2015. BAAD: a biomass and allometry database for woody plants. Ecology 96:1445.http://dx.doi.org/10.1890/14-1889.1 |
| Global Dataset of Maximum Rooting Depth | Fan Y, Gonzalo Miguez-Macho, Esteban G. Jobbágy, Robert B. Jackson, Carlos Otero-Casal (2017) Hydrologic regulation of plant rooting depth. Proceedings of the National Academy of Sciences 114 (40) 10572-10577; DOI: 10.1073/pnas.1712381114 |
| Fazlioglu et al. 2016 | Fazlioglu F, Al-Namazi A, Bonser SP. (2016) Reproductive efficiency and shade avoidance plasticity under simulated competition, Ecology and Evolution 6: 4947–4957. Doi: 10.1002/ece3.2254. |
| Fazlioglu et al. 2018_raw data | Fazlioglu F, Wan JSH, Bonser SP. (2018) Phenotypic plasticity and specialization along an altitudinal gradient in Trifolium repens, Turkish Journal of Botany 42: 440–447. Doi: 10.3906/bot-1711-21 |
| Fazlioglu, 2011, MSc Thesis | Fazlioglu, 2011, A phenetics for infrageneric grouping of Limonium Mill. genus (Plumbaginaceae) in Turkey. Master of Science thesis submitted to METU |
| shade (for TRY)_mvk | Feng Y & van Kleunen M (2014) Responses to shading of naturalized and non-naturalized exotic woody species. Annals of Botany 114:981-989. (DOI: 10.1093/aob/mcu163) |
| ladias: leaf traits in the Czech flora | Findurova A. (2018): Variability of leaf traits SLA and LDMC in selected species of the Czech flora. Ms thesis depon in Masaryk University, Brno |
| Ecological Flora of the British Isles | Fitter, A. H. and H. J. Peat 1994. The Ecological Flora Database. Journal of Ecology 82:415-425. |
| Fruit type, fruit dimension and flowering time | Flora of Australia online 2009. Australian Biological Resources study, Canberra. |
| Growth form data for 3581 Australian species | Flora of Australia online 2009. Australian Biological Resources study, Canberra. |
| Fonseca/Wright New South Wales Database | Fonseca, C. R., J. M. Overton, B. Collins, and M. Westoby. 2000. Shifts in trait-combinations along rainfall and phosphorus gradients. Journal of Ecology 88:964-977. |
| Traits and ecological strategies of 66 subtropical tree species in the Brazilian Atlantic Forest | Forgiarini, C., Souza, A.F., Longhi, S.J., Oliveira, J.M., 2015. In the lack of extreme pioneers: trait relationships and ecological strategies of 66 subtropical tree species. J. Plant Ecol. 8, 359–367. doi:10.1093/jpe/rtu028 |
| Yasuni Ecuador Leaf ITV | Fortunel C., I.R. McFadden, R. Valencia & N.J.B. Kraft. 2019. Neither species geographic range size, climatic envelope nor intraspecific leaf trait variability capture habitat specialization in a hyperdiverse Amazonian forest. Biotropica. in press |
| The VISTA Plant Trait Database | Fortunel, C., E. Garnier, R. Joffre, E. Kazakou, H. Quested, K. Grigulis, S. Lavorel, P. Ansquer, H. Castro, P. Cruz, J. Dolezal, O. Eriksson, H. Freitas, C. Golodets, C. Jouany, J. Kigel, M. Kleyer, V. Lehsten, J. Leps, T. Meier, R. Pakeman, M. Papadimitriou, V. P. Papanastasis, F. Quetier, M. Robson, M. Sternberg, J. P. Theau, A. Thebault, and M. Zarovali. 2009. Leaf traits capture the effects of land use changes and climate on litter decomposability of grasslands across Europe. Ecology 90:598-611. |
| Plant Functional Traits of Arid Steppes in Eastern Morocco (ECWP-Morocco) | Frenette-Dussault, C., Shipley, B., Léger, J.F., Meziane, D. & Hingrat, Y. (2012). Functional structure of an arid steppe plant community reveals similarities with Grime's C-S-R theory. Journal of Vegetation Science 23: 208-222. |
| Freschet et al. 2015 - Mount Hutt | Freschet GT, Kichenin E, Wardle DA. 2015. Explaining within-community variation in plant biomass allocation: a balance between organ biomass and morphology above vs below ground? Journal of Vegetation Science 26: 431-440. |
| Freschet Mount Hutt 1450 | Freschet GT, Kichenin E, Wardle DA. 2015. Explaining within-community variation in plant biomass allocation: a balance between organ biomass and morphology above vs below ground? Journal of Vegetation Science 26: 431-440. |
| Freschet et al. 2018 | Freschet GT, Violle C, Bourget MY, Scherer-Lorenzen M, Fort F. 2018. Allocation, morphology, physiology, architecture: the multiple facets of plant above and belowground responses to resource stress. New Phytologist 219: 1338-1352. |
| Traits from Subarctic Plant Species Database | Freschet, G. T., J. H. C. Cornelissen, R. S. P. van Logtestijn, and R. Aerts. 2010. Evidence of the ‘plant economics spectrum’ in a subarctic flora. Journal of Ecology 98:362-373. |
| Traits from Subarctic Plant Species Database | Freschet, G. T., J. H. C. Cornelissen, R. S. P. van Logtestijn, and R. Aerts. 2010. Substantial nutrient resorption from leaves, stems and roots in a sub-arctic flora: what is the link with other resource economics traits? New Phytologist 186:879-889. |
| The DIRECT Plant Trait Database | Fry, E.L., Power, S.A. Manning, P. (2014) Trait based classification and manipulation of functional groups in biodiversity-ecosystem function experiments. Journal of Vegetation Science, 25, 248-261. |
| The RAINFOR Plant Trait Database | Fyllas, N. M., S. Patino, T. R. Baker, G. Bielefeld Nardoto, L. A. Martinelli, C. A. Quesada, R. Paiva, M. Schwarz, V. Horna, L. M. Mercado, A. Santos, L. Arroyo, E. M. Jimenez, F. J. Luizao, D. A. Neill, N. Silva, A. Prieto, A. Rudas, M. Silviera, I. C. G. Vieira, G. Lopez-Gonzale, and J. Lloyd. 2009. Basin-wide variations in foliar properties of Amazonian forest: phylogeny, soils and climate Biogeosciences 6:2677-2708. |
| BASECO: a floristic and ecological database of Mediterranean French flora | Gachet S, Errol Véla, Thierry Tatoni, 2005, BASECO: a floristic and ecological database of Mediterranean French flora. Biodiversity and Conservation 14(4):1023-1034 |
| Climbing plants trait dataset | Gallagher RV, MR Leishman (2012) A global analysis of trait variation and evolution in climbing plants. Journal of Biogeography 39, 1757-1771. DOI: 10.1111/j.1365-2699.2012.02773.x |
| Climbing Plants Trait Database | Gallagher, R., M. R. Leishman, and A. T. Moles. 2011. Traits and ecological strategies of Australian tropical and temperate climbing plants. Journal of Biogeography. DOI: 10.1111/j.1365-2699.2010.02455.x. |
| The VISTA Plant Trait Database | Garnier, E., S. Lavorel, P. Ansquer, H. Castro, P. Cruz, J. Dolezal, O. Eriksson, C. Fortunel, H. Freitas, C. Golodets, K. Grigulis, C. Jouany, E. Kazakou, J. Kigel, M. Kleyer, V. Lehsten, J. Leps, T. Meier, R. Pakeman, M. Papadimitriou, V. P. Papanastasis, H. Quested, F. Quetier, M. Robson, C. Roumet, G. Rusch, C. Skarpe, M. Sternberg, J.-P. Theau, A. Thebault, D. Vile, and M. P. Zarovali. 2007. Assessing the effects of land-use change on plant traits, communities and ecosystem functioning in grasslands: A standardized methodology and lessons from an application to 11 European sites. Annals of Botany 99:967-985. |
| Ambrosia artemisiifolia Gentili | Gentili, R, Gilardelli, F, Ciappetta, S, Ghiani, A, Citterio, S ( 2015). Inducing competition: intensive grassland seeding to control Ambrosia artemisiifolia. Weed Research 55, 278– 288 |
| Species and trait shifts in Apennine grasslands | Giarrizzo E., Burrascano S., Chiti T., de Bello F., Leps J., Zavattero L., Blasi C. (2017) Re-visiting historical semi-natural grasslands in the Apennines to assess patterns of changes in plant species composition and functional traits. Applied Vegetation Science 20(2):247-258, doi: 10.1111/avsc.12288 |
| Species able to reproduce after fire in a Brazilian Savanna | Giroldo, Aelton (2016) Pequenas plantas, grandes estrategias: adaptacoes e sobrevivencia no Cerrado. PhD Thesis University of Brasilia. DOI: 10.13140/RG.2.2.34455.16800 |
| Hawaiian Lobeliad | Givnish T.J., R.A. Montgomery and G. Goldstein. 2004. Adaptive radiation of photosynthetic physiology in the Hawaiian lobeliads: light regimes, static light responses, and whole-plant compensation points. American Journal of Botany 91: 228-246 |
| Leaf traits data (SLA) for 56 woody species at the Smithsonian Conservation Biology Institute-Forest | Gonzalez-Akre, E., McShea, W., Bourg, N., Anderson-Teixeira, K. 2015. Leaf traits data (SLA) for 56 woody species at the Smithsonian Conservation Biology Institute-ForestGEO Forest Dynamic Plot. Front Royal, Virginia. USA. [Data set]. Version 1.0.(www.try-db.org) |
| French Alps Trait Data | Gos P., Loucougaray G., Colace MP., Arnoldi C., Gaucherand S., Dumazel D., Girard L., Delorme S., Lavorel S. (2016) Oecologia 180:1001 doi:10.1007/s00442-016-3551-3 |
| Diurnal and nocturnal gas exchange Quercus spp. | Granda E., Jose Javier Peguero-Pina, Domingo Sancho-Knapik, Eustaquio Gil Pelegrin, Víctor Resco de Dios. Functional significance of maintaining open stomata at night: a case study of multiple Quercus species (in preparation). |
| Seasonal gas exchange photoperiod Quercus spp. | Granda, E., Baumgarten, F., Gessler, A., Gil-Pelegrin, E., Peguero-Pina, J.J., Sancho-Knapik, D., E. Zimmerman, N., Resco de Dios, V. (2020). Day-length regulates seasonal patterns of stomatal conductance in Quercus species. Plant, Cell & Environment. DOI 10.1111/pce.13665 |
| PLANTSdata USDA | Green, W. 2009. USDA PLANTS Compilation, version 1, 09-02-02. (http://bricol.net/downloads/data/PLANTSdatabase/) NRCS: The PLANTS Database (http://plants.usda.gov, 1 Feb 2009). National Plant Data Center: Baton Rouge, LA 70874-74490 USA. |
| Jena Experiment Traits | Gubsch M, Buchmann N, Schmid B, Schulze E-D, Lipowsky A, Roscher C (2011) Differential effects of plant diversity on functional trait variation of grass species. Annals of Botany 107, 157-169. |
| VirtualForests Trait Database | Gutiérrez AG, & Huth A (2012) Successional stages of primary temperate rainforests of Chiloé Island, Chile. Perspectives in plant ecology, systematics and evolution. 14: 243– 256 |
| Saskatchewan Plant Trait Database | Guy, A. L., J. M. Mischkolz, and E. G. Lamb. 2013. Limited effects of simulated acidic deposition on seedling survivorship and root morphology of endemic plant taxa of the Athabasca Sand Dunes in well watered greenhouse trials. Botany 91:176-181. http://dx.doi.org/10.1139/cjb-2012-0162 |
| branch_anatomy | Hajek P, Kurjak D, von Wühlisch G, Delzon S, Schuldt B. 2016. Frontiers in Plant Science 7: 791, DOI: 10.3389/fpls.2016.00791 |
| branch_anatomy | Hajek P, Leuschner C, Hertel D, Delzon S, Schuldt B. 2014. Tree Physiology 34: 744–756, DOI: 10.1093/treephys/tpu048 |
| Cedrus atlantica traits | Hambuckers, A., Trolliet, F., Dury, M., Henrot, A.J., Porteman, K., Hasnaoui, Y.E., Bulcke, J.V.D., Mil, T.D., Remy, C.C., Cheddadi, R., François, L. Towards a More Realistic Simulation of Plant Species with a Dynamic Vegetation Model Using Field‐Measured Traits: The Atlas Cedar, a Case Study. Forests 2022, 13. 10.3390/f13030446 |
| Chinese Leaf Traits Database | Han, W. X., J. Y. Fang, D. L. Guo, and Y. Zhang. 2005. Leaf nitrogen and phosphorus stoichiometry across 753 terrestrial plant species in China. New Phytologist 168:377-385. |
| Leaf and Whole-Plant Traits Database: Hydraulic and Gas Exchange Physiology, Anatomy, Venation Struc | Hao, G. Y., L. Sack, A. Y. Wang, K. F. Cao, and G. Goldstein. 2010. Differentiation of leaf water flux and drought tolerance traits in hemiepiphytic and non-hemiepiphytic Ficus tree species. Functional Ecology 24:731-740. |
| Rede Amazônia Sustentável | Hawes, J.E., Viera, I.C.G., Magnago, L.F.S., Berenguer, E., Ferreira, J., Aragão, L., Cardoso, A., Lees, A.C., Lennox, G.D., Tobias, J.A., Waldron, A. & Barlow, J. (in review) A large-scale assessment of plant dispersal traits across human-modified Amazonian forests. Journal of Ecology |
| Leaf economic traits in soy | Hayes, F. J., Buchanan, S. W., Coleman, B., Gordon, A. M., Reich, P. B., Thevathasan, N. V., Wright, I.J., & Martin, A. R. (2018). Intraspecific variation in soy across the leaf economics spectrum. Annals of botany, 123(1), 107-120 |
| Chinese Leaf Traits Database | He, J. S., L. Wang, D. F. B. Flynn, X. P. Wang, W. H. Ma, and J. Y. Fang. 2008. Leaf nitrogen : phosphorus stoichiometry across Chinese grassland biomes. Oecologia 155:301-310. |
| Chinese Leaf Traits Database | He, J. S., Z. H. Wang, X. P. Wang, B. Schmid, W. Y. Zuo, M. Zhou, C. Y. Zheng, M. F. Wang, and J. Y. Fang. 2006. A test of the generality of leaf trait relationships on the Tibetan Plateau. New Phytologist 170:835-848. |
| Leaf Economics Traits of Woody Species in Dinghushan Biosphere Reserve, Southern China | He, P., Wright, I. J., Zhu, S., Onoda, Y., Liu, H., Li, R., ... & Ye, Q. (2019). Leaf mechanical strength and photosynthetic capacity vary independently across 57 subtropical forest species with contrasting light requirements. New Phytologist 223: 607每618 |
| Seed mass and nutrient concentration in Grevillea and Hakea species | He, T., Fowler, W. M. & Causley, C. L. (2015). High nutrient-use efficiency during early seedling growth in diverse Grevillea species (Proteaceae). Scientific Reports 5, 17132. doi: 10.1038/srep17132. |
| Fire Related Traits in Proteaceae and Pinaceae | He, T., Lamont, B. B. & Downs, K. S. (2011) Banksias born to burn. New Phytologist 191, 184—196. doi:10.1111/j.1469-8137.2011.03663.x. |
| Fire Related Traits in Proteaceae and Pinaceae | He, T., Pausas, J.P., Belcher, C.M., Schwilk, D. W. & Lamont, B. B. (2012) Fire-adapted traits of Pinus arose in the fiery Cretaceous. New Phytologist 194, 751—759. doi: 10.1111/j.1469-8137.2012.04079.x. |
| Peel Forest New Zealand Sycamore dataset | Heberling, J.M. & Mason, N.W.H. (2019) Are endemics functionally distinct?: Leaf traits of native and invasive woody species in a New Zealand forest. PLOS ONE 13(5): e0196746 |
| Coastal grasslands with Rosa rugosa (Hokkaido, Japan) | Helsen K, Matsushima H, Somers B, Honnay O (2021) A trait-based approach across the native and invaded range to understand plant invasiveness and community impact. Oikos, in press. https://doi.org/10.1111/oik.08034 |
| riparian forb traits W-Europe | Helsen K, Smith SW, Brunet J, et al (2018) Impact of an invasive alien plant on litter decomposition along a latitudinal gradient. Ecosphere 9:e02097. doi: 10.1002/ecs2.2097 |
| Coastal grasslands invaded by Rosa rugosa (Belgium) | Helsen K, Van Cleemput E, Bassi L, Graae BJ, Somers B, Blonder B & Honnay O (2020) Inter- and intraspecific trait variation shape multidimensional trait overlap between two plant invaders and the invaded communities. Oikos, 129(5): 677–688. |
| Trait and biomass data 2014 and 2015 of the BE_LOW project | Herz, K., Dietz, S., Haider, S., Jandt, U., Scheel, D. & Bruelheide, H. (2017): Drivers of intraspecific trait variation of grass and forb species in German meadows and pastures. – Journal of Vegetation Science 28: 705–716. Doi: 10.1111/jvs.12534. Herz, K., Dietz, S., Haider, S., Jandt, U., Scheel, D. & Bruelheide, H. (2017): Predicting individual plant performance in grasslands. – Ecology and Evolution 7: 8958–8965. DOI: 10.1002/ece3.3393 |
| Herbaceous Traits from the Öland Island Database | Hickler, T. 1999. Plant functional types and community characteristics along environmental gradients on Öland's Great Alvar (Sweden) Masters Thesis, University of Lund, Sweden. |
| Panama wood anatomy | Hietz, P., Rosner, S., Hietz-Seifert, U. & Wright, S.J. (2017). Wood traits related to size and life history of trees in a Panamanian rainforest. New Phytol., 213, 170-180 |
| Araucaria Forest Database | HIGUCHI, P.; SILVA, A.C. Araucaria Forest Database. 2013 |
| PLANTATT - Attributes of British and Irish Plants | HILL, M.O., PRESTON, C.D. & ROY, D.B. (2004) PLANTATT - attributes of British and Irish Plants: status, size, life history, geography and habitats. Huntingdon: Centre for Ecology and Hydrology. |
| Morton Arboretum Experimental Prairie traitset 1, 2019 | Hipp, A.L., Glasenhardt, M.-C., Bowles, M.L., Garner, M., Scharenbroch, B.C., Williams, E.W., Barak, R.S., Byrne, A., Ernst, A.R., Grigg, E., Midgley, M.G., Wagreich, H., and Larkin, D.J. 2018. Effects of Phylogenetic Diversity and Phylogenetic Identity in a Restoration Ecology Experiment. In Phylogenetic Diversity: Applications and Challenges in Biodiversity Science. Edited by R.A. Scherson and D.P. Faith. Springer International Publishing, Cham. pp. 189–210. doi:10.1007/978-3-319-93145-6_10 |
| branch_anatomy | Hoeber S, Leuschner C, Köhler L, Arias-Aguilar D, Schuldt B. 2014. Forest Ecology and Management 330: 126–136, DOI: 10.1016/j.foreco.2014.06.039 |
| Leaf functional traits for tropical saplings from Jianfengling, Hainan Island, China | Hogan J.A., Oscar J. Valverde-Barrantes, Han Xu & Christopher Baraloto, Intraspecific root and leaf trait variation with tropical forest successional status: consequences for community-weighted patterns (in review) |
| Leaf and Whole-Plant Traits Database: Hydraulic and Gas Exchange Physiology, Anatomy, Venation Struc | Hoof, J., L. Sack, D. T. Webb, and E. T. Nilsen. 2008. Contrasting structure and function of pubescent and glabrous varieties of Hawaiian Metrosideros polymorpha (Myrtaceae) at high elevation. Biotropica 40:113-118. |
| Biomass allocation of Carex obnupta and Carex stipata | Hough-Snee N, Nackley LL, Kim S, Ewing K (2015) Does plant performance under stress explain divergent life history strategies? The effects of flooding and nutrient stress on two wetland sedges. Aquatic Botany 120(B):151–159 DOI:10.1016/j.aquabot.2014.03.001 |
| FRED - Fine Root Ecology Database | Iversen, C. M., McCormack, M. L., Powell, A. S., Blackwood, C. B., Freschet, G. T., … Violle, C. (2017), A global Fine-Root Ecology Database to address below-ground challenges in plant ecology. New Phytol, 215: 15-26. doi:10.1111/nph.14486 |
| Effect of drought on pine needle traits | Jactel, H., Castagneyrol, B. 2019. Effect of drought on maritime pine needle traits in ORPHEE (https://sites.google.com/view/orpheeexperiment/home) |
| NZ tree traits | Jager et al (2015) Journal of Ecology 103(2):374-385. https://doi.org/10.1111/1365-2745.12366 ; Simpson et al. (2016) Global Ecology and Biogeography 25:964-978 doi:10.1111/geb.12457 |
| Costa Rican Tropical Dry Forest Trees | Jennifer S. Powers and Peter Tiffin 2012 Plant functional type classifications in tropical dry forests in Costa Rica: leaf habit versus taxonomic approaches. Functional Ecology 2010, 24, 927–936 doi: 10.1111/j.1365-2435.2010.01701.x |
| SLA data La Palma 2019 (SLA project) | Jentsch A, Disturbance Ecology, University of Bayreuth sampled by Patrick Aurich, Karina Krapf, Tsu-Yun Chiu |
| Miombo tree species - SLA, leaf and seed size | Joseph, G.S., Seymour, C.L., Cumming, G.S., Cumming, D.H.M., & Mahlangu, Z. 2014. Termite mounds increase functional diversity of woody plants in African savannas. Ecosystems 17: 808–819. |
| AlpinePlants_Austria | Junker R.R., Martin H. Lechleitner, Jonas Kuppler, Lisa-Maria Ohler (in revision) INTERCONNECTEDNESS OF THE GRINNELLIAN AND ELTONIAN NICHE IN REGIONAL AND LOCAL PLANT-POLLINATOR COMMUNITIES. Front. Plant Sci. |
| Traits of Plants in Canada (TOPIC) | Kabzems, R. EP 1133 Aspen foliar Sierra / Fort Nelson. BC Ministry of Forests, Lands and Natural Resource Operations. Unpublished data. |
| Pladias: Life forms and heights of the Czech flora | Kaplan Z., Danihelka J., Chrtek J. jun., Kirschner J., Kubát K., Štech M. & Štěpánek J. (eds) (2019): Klíč ke květeně České republiky [Key to the flora of the Czech Republic]. Ed. 2. Academia, Praha. |
| Photosynthesis Type Database | Kapralov MV, Smith JAC, Filatov DA (2012) Rubisco Evolution in C4 Eudicots: An Analysis of Amaranthaceae Sensu Lato. PLoS ONE 7(12): e52974. doi:10.1371/journal.pone.0052974 |
| Functional traits and environmental conditions of Thymus praecox in Central German badlands | Karbstein K, Tomasello S, Prinz K. Desert-like badlands and surrounding (semi-)dry grasslands of Central Germany promote small-scale phenotypic and genetic differentiation in Thymus praecox. Ecology and Evolution 2019;9:14066–14084. https://doi.org/10.1002/ece3 |
| Functional traits and within habitat characteristics of Trifolium montanum (mountain clover) in Cent | Karbstein, K, Prinz, K, Hellwig, F, Römermann, C. Plant intraspecific functional trait variation is related to within?habitat heterogeneity and genetic diversity in Trifolium montanum L. Ecol Evol. 2020; 10: 5015– 5033. https://doi.org/10.1002/ece3.6255 |
| KIT herbaceous functional gradient (weekly measurements) | Kattenborn, T., & Schmidtlein, S. (2019). Radiative transfer modelling reveals why canopy reflectance follows function. Scientific reports, 9(1), 6541. |
| KIT herbaceous functional gradient (median) | Kattenborn, T., Fassnacht, F. E., & Schmidtlein, S. (2018). Differentiating plant functional types using reflectance: which traits make the difference? Remote Sensing in Ecology and Conservation, 1–15. http://doi.org/10.1002/rse2.86 |
| Leaf Physiology Database | Kattge, J., W. Knorr, T. Raddatz, and C. Wirth. 2009. Quantifying photosynthetic capacity and its relationship to leaf nitrogen content for global-scale terrestrial biosphere models. Global Change Biology 15:976-991. |
| Ecophysiological parameters of tree and shrub leaves in forest-steppe plantings | Kavelenova L.M., Rozno S.A., Kireyeva YU.V., Smirnov YU.V. K strukturno-funktsionalnym osobennostyam listyev drevesnykh rasteniy v nasazhdeniyakh lesostepi // Samarskaya Luka. Byulleten. 2007. T.16. No 3 (21). P.568 – 574. |
| Leaf and Whole Plant Traits Database | Kazakou, E., D. Vile, B. Shipley, C. Gallet, and E. Garnier. 2006. Co-variations in litter decomposition, leaf traits and plant growth in species from a Mediterranean old-field succession. Functional Ecology 20:21-30. |
| Yangambi (DR Congo) tropical forest tree traits | Kearsley, E., Verbeeck, H., Hufkens, K., Van de Perre, F., Doetterl, S., Baert, G., Beeckman, H., Boeckx, P., Huygens, D. (2017). Functional community structure of African monodominant Gilbertiodendron dewevrei forest influenced by local environmental filtering. Ecology and Evolution, 7, 295–304 |
| Competition | Kempel A, Chrobock T, Fischer M, Rohr RP & van Kleunen M (2013) Determinants of plant establishment success in a multispecies introduction experiment with native and alien species. Proceedings of the National Academy of Sciences 110:12727-12732 |
| The Americas N&P database | Kerkhoff, A. J., W. F. Fagan, J. J. Elser, and B. J. Enquist. 2006. Phylogenetic and growth form variation in the scaling of nitrogen and phosphorus in the seed plants. American Naturalist 168:E103-E122. |
| Khalil Prairie Plant Traits | Khalil, M. I., Gibson, D. J., Baer, S. G. & Willand, J. E. 2018. Functional diversity is more sensitive to biotic filters than phylogenetic diversity during community assembly. Ecosphere, Art: e02164. Doi: 10.1002/ecs2.2164 |
| Khalil Prairie Plant Traits | Khalil, M.I., D.J. Gibson, & S.G. Baer. 2019. Functional response of subordinate species to intraspecific trait variability within dominant species. Journal of Ecology 107: in press |
| Leaf Traits Mount Hutt, New Zealand | Kichenin et al. 2013. Contrasting effects of plant inter- and intraspecific variation on community-level trait measures along an environmental gradient. Functional Ecology, in press. |
| KEW African Plant Traits Database | Kirkup, D., P. Malcolm, G. Christian, and A. Paton. 2005. Towards a digital African Flora. Taxon 54:457-466. |
| Orchid Trait Dataset | Kisel, Y., Moreno-Letelier, A. C., Bogarín, D., Powell, M. P., Chase, M. W. and Barraclough, T. G. (2012), TESTING THE LINK BETWEEN POPULATION GENETIC DIFFERENTIATION AND CLADE DIVERSIFICATION IN COSTA RICAN ORCHIDS. Evolution, 66: 3035–3052. doi: 10.1111/j.1558-5646.2012.01663.x |
| PalmTraits_1.0 | Kissling, D. W., Balslev, H., Baker, W. J., Dransfield, J., Göldel, B., Lim, J. Y., Onstein, R. E., Svenning, J.-C. (2019) PalmTraits 1.0, a species-level functional trait database of palms worldwide. Scientific Data. DOI: https://doi.org/10.1038/s41597-019-0189-0 |
| The LEDA Traitbase | Kleyer, M., R. M. Bekker, I. C. Knevel, J. P. Bakker, K. Thompson, M. Sonnenschein, P. Poschlod, J. M. van Groenendael, L. Klimes, J. Klimesova, S. Klotz, G. M. Rusch, Hermy, M. , D. Adriaens, G. Boedeltje, B. Bossuyt, A. Dannemann, P. Endels, L. Götzenberger, J. G. Hodgson, A.-K. Jackel, I. Kühn, D. Kunzmann, W. A. Ozinga, C. Römermann, M. Stadler, J. Schlegelmilch, H. J. Steendam, O. Tackenberg, B. Wilmann, J. H. C. Cornelissen, O. Eriksson, E. Garnier, and B. Peco. 2008. The LEDA Traitbase: a database of life-history traits of the Northwest European flora. Journal of Ecology 96:1266-1274. |
| CLO-PLA : a Database of Clonal Growth in Plants | Klimesova J. & de Bello F. CLO-PLA: the database of clonal and bud bank traits of Central European flora. Journal of Vegetation Science 20: 511-516. |
| CLO-PLA : a Database of Clonal Growth in Plants | Klimesova J. & Klimes L. Clo-Pla3 – database of clonal growth of plants from Central Europe. http://clopla.butbn.cas.cz/ |
| BiolFlor Database | Klotz, S. & Kühn, I. (2002): Blattmerkmale. - In: Klotz, S., Kühn, I. & Durka, W. [Hrsg.]: BIOLFLOR - Eine Datenbank zu biologisch-ökologischen Merkmalen der Gefäßpflanzen in Deutschland. - Schriftenreihe für Vegetationskunde 38: 119-126. Bundesamt für Naturschutz, Bonn. |
| BiolFlor Database | Klotz, S. & Kühn, I. (2002): Indikatoren zum anthropogenen Einfluss auf die Vegetation. In: Klotz, S., Kühn, I. & Durka, W. [eds.]: BIOLFLOR - Eine Datenbank zu biologisch-ökologischen Merkmalen der Gefäßpflanzen in Deutschland. - Schriftenreihe für Vegetationskunde 38: 241-246. Bundesamt für Naturschutz, Bonn. |
| BiolFlor Database | Klotz, S. & Kühn, I. (2002): Soziologische Bindung der Arten. In: Klotz, S., Kühn, I. & Durka, W. [eds.]: BIOLFLOR - Eine Datenbank zu biologisch-ökologischen Merkmalen der Gefäßpflanzen in Deutschland. - Schriftenreihe für Vegetationskunde 38: 273-281. Bundesamt für Naturschutz, Bonn. |
| BiolFlor Database | Klotz, S. & Kühn, I. (2002): Ökologische Strategietypen. - In: Klotz, S., Kühn, I. & Durka, W. [Hrsg.]: BIOLFLOR - Eine Datenbank zu biologisch-ökologischen Merkmalen der Gefäßpflanzen in Deutschland. - Schriftenreihe für Vegetationskunde 38: 197-201. Bundesamt für Naturschutz, Bonn. |
| BiolFlor Database | Klotz, S., Kühn, I. & Durka, W. [eds.]: BIOLFLOR - Eine Datenbank zu biologisch-ökologischen Merkmalen der Gefäßpflanzen in Deutschland. - Schriftenreihe für Vegetationskunde 38. Bundesamt für Naturschutz, Bonn. |
| Global Leaf Gas Exchange Database (II) | Knauer et al. (2017) Towards physiologically meaningful water-use efficiency estimates from eddy covariance data. Global Change Biology, DOI: 10.1111/gcb.13893 |
| Plant Trait Database in East and South-East Asia | Koike, F. 2001. Plant traits as predictors of woody species dominance in climax forest communities. Journal of Vegetation Science 12: 327-336 |
| Plant Trait Database in East and South-East Asia | Koike, M. Clout, M. Kawamichi, M. De Poorter and K. Iwatsuki eds. 2006. Assessment and Control of Biological Invasion Risks. Cambridge, UK and Shoukadoh Book Sellers, Kyoto, Japan, and IUCN, Gland, Switzerland. |
| Plant traits from Andorra | Komac B, C Pladevall, M Domenech and R Fanlo (2014) Functional diversity and grazing intensity in sub-alpine and alpine grasslands in Andorra. Applied Vegetation Science Doi: 10.1111/avsc.12119 |
| branch_anatomy | Kotowska M, Hertel D, Abou Rajab Y, Barus H, Schuldt B. 2015. Frontiers in Plant Science 6: 191, DOI: 10.3389/fpls.2015.00191 |
| BiolFlor Database | Krumbiegel, A. (2002): Morphologie der vegetativen Organe (außer Blätter). In: Klotz, S., Kühn, I. & Durka, W. [eds.]: BIOLFLOR - Eine Datenbank zu biologisch-ökologischen Merkmalen der Gefäßpflanzen in Deutschland. - Schriftenreihe für Vegetationskunde 38: 93-118. Bundesamt für Naturschutz, Bonn. |
| ACi-TGlob_V1.0: A Global dataset of photosynthetic CO2 response curves of terrestrial plants | Kumarathunge, D. P., Medlyn, B. E., Drake, J. E., Tjoelker, M. G., Aspinwall, M. J., Battaglia, M. , Cano, F. J., Carter, K. R., Cavaleri, M. A., Cernusak, L. A., Chambers, J. Q., Crous, K. Y., De Kauwe, M. G., Dillaway, D. N., Dreyer, E. , Ellsworth, D. S., Ghannoum, O. , Han, Q. , Hikosaka, K. , Jensen, A. M., Kelly, J. W., Kruger, E. L., Mercado, L. M., Onoda, Y. , Reich, P. B., Rogers, A. , Slot, M. , Smith, N. G., Tarvainen, L. , Tissue, D. T., Togashi, H. F., Tribuzy, E. S., Uddling, J. , Vårhammar, A. , Wallin, G. , Warren, J. M. and Way, D. A. (2019), Acclimation and adaptation components of the temperature dependence of plant photosynthesis at the global scale. New Phytol, 222: 768-784. doi:10.1111/nph.15668 |
| Hawaii Floral traits | Kuppler J, Hoefers MK, Trutschnig W, Bathke AC, Eiben JA, Daehler CC & Junker RR (2017) Exotic flower visitors exploit large floral trait spaces resulting in asymmetric resource partitioning with native visitors. Functional Ecology, 31, 2244-2254 |
| Traits of Bornean Trees Database | Kurokawa, H. and T. Nakashizuka. 2008. Leaf herbivory and decomposability in a Malaysian tropical rain forest. Ecology 89:2645-2656. |
| BiolFlor Database | Kühn, I. Klotz, S. (2002): Angaben zu den Arealen. In: Klotz, S., Kühn, I. & Durka, W. [eds.]: BIOLFLOR - Eine Datenbank zu biologisch-ökologischen Merkmalen der Gefäßpflanzen in Deutschland. - Schriftenreihe für Vegetationskunde 38: 227-239. Bundesamt für Naturschutz, Bonn. |
| BiolFlor Database | Kühn, I., Klotz, S. (2002): Floristischer Status und gebietsfremde Arten. In: Klotz, S., Kühn, I. & Durka, W. [eds.]: BIOLFLOR - Eine Datenbank zu biologisch-ökologischen Merkmalen der Gefäßpflanzen in Deutschland. - Schriftenreihe für Vegetationskunde 38: 47-56. Bundesamt für Naturschutz, Bonn. |
| BiolFlor Database | Kühn, I., W. Durka, and S. Klotz. 2004. BiolFlor - a new plant-trait database as a tool for plant invasion ecology. Diversity and Distribution 10 363-365. |
| Plant traits of grassland species | La Pierre, KJ and Smith, MD. (2015) Functional trait expression of grassland species shift with short- and long-term nutrient additions. Plant Ecology 216: 307 doi:10.1007/s11258-014-0438-4 |
| Traits of Plants in Canada (TOPIC) | Lafleur, B. 2018. Foliar-Lafleur Peuplier 2018. Université de Québec en Abitibi-Témiscamingue. Unpublished dataset |
| Traits of Plants in Canada (TOPIC) | Lafleur, B., Fenton, N.J., Pare, D., Simard, M. and Bergeron, Y. (2010) Contrasting effects of season and method of harvest on soil properties and the growth of black spruce regeneration in the boreal forested Peatlands of Eastern Canada. Silva Fennica, 44(5): 799-813 |
| Traits of Plants in Canada (TOPIC) | Lafleur, B., Pare, D., Fenton, N.J. and Bergeron, Y. (2011) Growth and nutrition of black spruce seedlings in response to disruption of Pleurozium and Sphagnum moss carpets in boreal forested peatlands. Plant and Soil, 345(1):141-153 |
| Traits of Plants in Canada (TOPIC) | Lafleur, B., Pare, D., Fenton, N.J. and Bergeron, Y. (2011) Growth of planted black spruce seedlings following mechanical site preparation in boreal forested peatlands with variable organic layer thickness: 5-year results. Annals of Forest Science, 68:1291-1302. |
| Plant Traits for Pinus and Juniperus Forests in Arizona | Laughlin, D. C., J. J. Leppert, M. M. Moore, and C. H. Sieg. 2010. A multi-trait test of the leaf-height-seed plant strategy scheme with 133 species from a pine forest flora. Functional Ecology 24:493-501. |
| Plant Traits for Pinus and Juniperus Forests in Arizona | Laughlin, D.C., P.Z. Fulé, D.W. Huffman, J. Crouse, and E. Laliberte. 2011. Climatic constraints on trait-based forest assembly. Journal of Ecology 99:1489-1499. |
| Phalaris Arundinacea Genotypes | Lavergne S, Jane Molofsky (2007) Increased genetic variation and evolutionary potential drive the success of an invasive grass. Proceedings of the National Academy of Sciences 104(10):3883-3888; DOI: 10.1073/pnas.0607324104 |
| Phalaris Arundinacea Genotypes | Lavergne, S., Muenke, N. J. & Molofsky, J. (2009) Genome size reduction can trigger rapid phenotypic evolution in invasive plants. Annals of Botany 105, 109-116, doi:10.1093/aob/mcp271 |
| French Alps Trait Data | Lavorel, S., Grigulis, K., Lamarque, P., Colace, M.-P., Garden, D., Girel, J., Pellet, G. and Douzet, R. (2011), Using plant functional traits to understand the landscape distribution of multiple ecosystem services. Journal of Ecology, 99: 135–147. doi:10.1111/j.1365-2745.2010.01753.x |
| Palms_individual_measurements_2021 | Lenters T.P., Andrew Henderson, Caroline M. Dracxler, Guilherme A. Elias, Suzanne Mogue Kamga, Thomas L.P. Couvreur, W. Daniel Kissling (2021) Integration and harmonization of trait data from plant individuals across heterogeneous sources, Ecological Informatics, Volume 62, 101206, ISSN 1574-9541, https://doi.org/10.1016/j.ecoinf.2020.101206 |
| Saskatchewan Plant Trait Database | Letts, B., Lamb, E.G., Mischkolz, J.M. & Romo, J.T. (2015) Litter accumulation drives grassland plant community composition and functional diversity via leaf traits. Plant Ecology, 216, 357-370. |
| Traits of the Hungarian flora | Lhotsky B., Anikó Csecserits, Bence Kovács, Zoltán Botta-Dukát: New plant trait records of the Hungarian flora |
| Tree species functional traits from Dinghushan Biosphere Reserve, southern China | Li, R., Zhu, S., Chen, H. Y. H., John, R., Zhou, G., Zhang, D., Zhang, Q. and Ye, Q. (2015), Are functional traits a good predictor of global change impacts on tree species abundance dynamics in a subtropical forest?. Ecol Lett, 18: 1181–1189. doi:10.1111/ele.12497 |
| Sherbrooke | Li, Y. and Shipley, B. (2018) Community divergence and convergence along experimental gradients of stress and disturbance. Ecology, 99: 775-781. doi:10.1002/ecy.2162 |
| Heishiding_Performance_Trait_Environment_Data | Li, Y., Jiang, Y., Shipley, B., Li, B., Luo, W., Chen, Y., Zhao, K., He, D., Rodríguez-Hernández, D.I. and Chu, C. (2021), The complexity of trait–environment performance landscapes in a local subtropical forest. New Phytol, 229: 1388-1397. https://doi.org/10.1111/nph.16955 |
| Foliar_CNP_under_sheep_grazing | Liang, M., Gornish, E.S., Mariotte, P., Chen, J. & Liang, C. (2019). Foliar nutrient content mediates grazing effects on species dominance and plant community biomass. Rangeland Ecology & Management, 72, 899-906. https://doi.org/10.1016/j.rama.2019.08.001 |
| Global Leaf Gas Exchange Database (I) | Lin Y-S, Medlyn BE, Duursma RA, Prentice IC, Wang H, Baig S, Eamus D, De Dios VR, Mitchell P, Ellsworth DS, De Beeck MO, Wallin G, Uddling J, Tarvainen L, Linderson M-L, Cernusak LA, Nippert JB, Ocheltree TW, Tissue DT, Martin-StPaul NK, Rogers A, Warren JM, De Angelis P, Hikosaka K, Han Q, Onoda Y, Gimeno TE, Barton CVM, Bennie J, Bonal D, Bosc A, Löw M, Macinins-Ng C, Rey A, Rowland L, Setterfield SA, Tausz-Posch S, Zaragoza-Castells J, Broadmeadow MSJ, Drake JE, Freeman M, Ghannoum O, Hutley LB, Kelly JW, Kikuzawa K, Kolari P, Koyama K, Limousin J-M, Meir P, Da Costa ACL, Mikkelsen TN, Salinas N, Sun W, Wingate L, (2015) Optimal stomatal behaviour around the world. Nature Climate Change 5(5): 459-464 DOI: 10.1038/NCLIMATE2550 |
| Fern Traits in Subtropical Forest | Lin, D., Yang, S., Dou, P., Wang, H., Wang, F., Qian, S., ... & Fanin, N. (2020). A plant economics spectrum of litter decomposition among coexisting fern species in a sub-tropical forest. Annals of Botany, 125(1), 145-155 |
| Jena Experiment Traits | Lipowsky A, Roscher C, Schumacher J, Michalski SG, Gubsch M, Buchmann N, Schulze E-D, Schmid B (2015) Plasticity of functional traits of forb species in response to biodiversity. Perspectives in Plant Ecology, Evolution and Systematics 17, 66-77 |
| Chajul secondary forest species | Lohbeck, M., L. Poorter, H. Paz, L. Pla, M. van Breugel, M. Martínez-Ramos, and F. Bongers. 2012. Functional diversity changes during tropical forest succession. Perspectives in Plant Ecology, Evolution and Systematics 14:89–96 |
| Individual-based traits in alpine vegetation | Losapio G, De la Cruz M, Escudero A, Schmid B & Schöb C. 2018. The assembly of a plant network in alpine vegetation. Journal of Vegetation Science, 29, 999-1006 |
| French Massif Central Grassland Trait Database | Louault, F., V. D. Pillar, J. Aufrere, E. Garnier, and J. F. Soussana. 2005. Plant traits and functional types in response to reduced disturbance in a semi-natural grassland. Journal of Vegetation Science 16:151-160. |
| Plant Physiology Database | Loveys, B. R., L. J. Atkinson, D. J. Sherlock, R. L. Roberts, A. H. Fitter, and O. K. Atkin. 2003. Thermal acclimation of leaf and root respiration: an investigation comparing inherently fast- and slow-growing plant species. Global Change Biology 9:895-910. |
| Structural and biochemical leaf traits of boreal tree species in Finland | Lukeš, P., Stenberg, P., Rautiainen, M., Mõttus, M., Vanhatalo, K.M. Optical properties of leaves and needles for boreal tree species in Europe (2013) Remote Sensing Letters, 4 (7), pp. 667-676 |
| Intraspecific variation leaf traits temperate rainforest | Lusk, C. H. (2019). Leaf functional trait variation in a humid temperate forest, and relationships with juvenile tree light requirements. PeerJ, 7, e6855 |
| Traits of temperate rainforest tree seedlings from New Zealand | Lusk, C. H., Kaneko, T., Grierson, E. and Clearwater, M. (2013) Correlates of tree species sorting along a temperature gradient in New Zealand rain forests: seedling functional traits, growth and shade tolerance. Journal of Ecology, 101: 1531–1541. |
| branch_anatomy | Lübbe T, Schuldt B, Leuschner C. 2017. Tree Physiology 37: 456-468, DOI: 10.1093/treephys/tpw095 |
| Photosynthesis Traits Worldwide | Maire V, Ian J. Wright, I. Colin Prentice, Niels H. Batjes, Radika Bhaskar, Peter M. van Bodegom, Will K. Cornwell, David Ellsworth, Ülo Niinemets, Alejandro Ordoñez, Peter B. Reich, Louis S. Santiago (2015). Global soil and climate effects on leaf photosynthetic traits and rates. Global Ecology and Biogeography 24(6): 706-717. Maire V, Wright IJ, Prentice IC, Batjes NH, Bhaskar R, van Bodegom PM, Cornwell WK, Ellsworth D, Niinemets Ü, Ordoñez A, Reich PB, Santiago LS (2015) Data from: Global effects of soil and climate on leaf photosynthetic traits and rates. Dryad Digital Repository. http://dx.doi.org/10.5061/dryad.j42m7 |
| Traits of Plants in Canada (TOPIC) | Mallik, A. and St-Martin, P. 2013. Kalmia angustifolia leaf traits from Newfoundland. Lakehead University. Unpublished data. |
| Plant Hydraulic Traits | Manzoni S, Giulia Vico, Amilcare Porporato, Gabriel Katul (2013) Biological constraints on water transport in the soil–plant–atmosphere system. Advances in Water Resources 51:292–304 |
| Traits from the Wildfire Project | Marco Moretti and Colin Legg (2009) Combining plant and animal traits to assess community functional responses to disturbance. Ecography 32: 299 309. doi: 10.1111/j.1600-0587.2008.05524.x |
| Leaf and Whole-Plant Traits Database: Hydraulic and Gas Exchange Physiology, Anatomy, Venation Struc | Markesteijn, L., L. Poorter, H. Paz, L. Sack, and F. Bongers. 2011. Ecological differentiation in xylem cavitation resistance is associated with stem and leaf structural traits. Plant, Cell and Environment 34:137-148. |
| Leaf economic traits in wheat and maize | Martin A R, Christine E Hale, Bruno E L Cerabolini, Johannes H C Cornelissen, Joseph Craine, William A Gough, Jens Kattge, Cairan K F Tirona (2018) Inter- and intraspecific variation in leaf economics traits in wheat and maize, AoB PLANTS, ply006, |
| Live fuel moisture data at a pine forest | Martin S., M., Bonet, J.A., Martínez De Aragón, J., Voltas, J., Coll, L. & Resco De Dios, V. (2017) Crown bulk density and fuel moisture dynamics in Pinus pinaster stands are neither modified by thinning nor captured by the Forest Fire Weather Index. Annals of Forest Science, 74, 51. |
| Leaf economic traits in coffee | Martin, A. R., Rapidel, B., Roupsard, O., Van den Meersche, K., de Melo Virginio Filho, E., Barrios, M., & Isaac, M. E. (2017). Intraspecific trait variation across multiple scales: the leaf economics spectrum in coffee. Functional Ecology, 31(3), 604-612 |
| Leaf and Whole-Plant Traits Database: Hydraulic and Gas Exchange Physiology, Anatomy, Venation Struc | Martin, R. E., G. P. Asner, and L. Sack. 2007. Genetic variation in leaf pigment, optical and photosynthetic function among diverse phenotypes of Metrosideros polymorpha grown in a common garden. Oecologia 151:387-400. |
| LosTuxtlas_functionaltraits | Martinez-Garza, C., F. Bongers, and L. Poorter. 2013a. Are functional traits good predictors of species performance in restoration plantings in tropical abandoned pastures? Forest Ecology and Management 303:35-45 |
| CNP seed stoichiometry | Maskova T., Herben T. (2021) Interspecific differences in maternal support in herbaceous plants: CNP contents in seeds varies to match expected nutrient limitation of seedlings. Oikos, 130, 1715–1725. https://doi.org/10.1111/oik.08186 |
| Traits of Plants in Canada (TOPIC) | Masse J., Prescott C.E., Müller C. and Grayston S.J. (2016) "Gross nitrogen transformation rates differ in reconstructed oil-sands soils from naturally disturbed boreal forest soils as revealed using 15N tracing method". Geoderma. 282. |
| Woody plant traits from southeast Queensland, Australia | McCarthy, J. K. (2018). Predicting the diversity and functional composition of woody plant communities under climate change. PhD Thesis. School of Biological Sciences, The University of Queensland. https://doi.org/10.14264/uql.2018.134 |
| Woody plant traits from southeast Queensland, Australia | McCarthy, J. K., Mokany, K., & Dwyer, J. M. (2019). A regional-scale assessment of using metabolic scaling theory to predict ecosystem properties. Proceedings of the Royal Society B: Biological Sciences, doi: 10.1098/rspb.2019-2221 |
| Fonseca/Wright New South Wales Database | McDonald, P. G., C. R. Fonseca, J. M. Overton, and M. Westoby. 2003. Leaf-size divergence along rainfall and soil-nutrient gradients: is the method of size reduction common among clades? Functional Ecology 17:50-57. |
| Yasuni Ecuador Leaf Drought Tolerance and Mechanical Toughness | McFadden, I.R., M.K. Bartlett, T. Wiegand, B. Turner, L. Sack, R. Valencia and N.J.B. Kraft. Disentangling the functional trait correlates of spatial aggregation in tropical forest trees. Ecology, accepted |
| Minimum Freezing Exposure Database | McGlinn, D.J. Global plant species freezing exposure database. Data from: Three keys to the radiation of angiosperms into freezing environments. Nature. Dryad Digital Repository. http://dx.doi.org/10.5061/dryad.63q27/4. (2013). |
| Leaf and Whole Plant Traits Database | McKenna, M. F. and B. Shipley. 1999. Interacting determinants of interspecific relative growth: Empirical patterns and a theoretical explanation. Ecoscience 6:286-296. |
| ECOCRAFT | Medlyn, B. E. and P. G. Jarvis. 1999. Design and use of a database of model parameters from elevated [CO2] experiments. Ecological Modelling 124:69-83. |
| ECOCRAFT | Medlyn, B. E., C. V. M. Barton, M. S. J. Broadmeadow, R. Ceulemans, P. DeAngelis, M. Forstreuter, M. Freeman, S. B. Jackson, S. Kellomaeki, E. Laitat, A. Rey, P. Roberntz, B. D. Sigurdsson, J. Strassemeyer, K. Wang, P. S. Curtis, and P. G. Jarvis. 2001. Stomatal Conductance of forest species after long-term exposure to elevated CO2 concentration: a synthesis. New Phytologist 149:247--264. |
| ECOCRAFT | Medlyn, B. E., F.-W. Badeck, D. G. G. De Pury, C. V. M. Barton, M. Broadmeadow, R. Ceulemans, P. De Angelis, M. Forstreuter, M. E. Jach, S. Kellomäki, E. Laitat, M. Marek, S. Philippot, A. Rey, J. Strassemeyer, K. Laitinen, R. Liozon, B. Portier, P. Roberntz, K. Wang, and P. G. Jarvis. 1999. Effects of elevated CO2 on photosynthesis in European forest species: a meta-analysis of model parameters. Plant, Cell and Environment 22:1475-1495. |
| Photosynthetic Capacity Dataset | Meir, P. & Levy, P. E. 2007 Photosynthetic parameters from two contrasting woody vegetation types in West Africa. Plant Ecology. 192, 2, p. 277-287 11 p. |
| Photosynthetic Capacity Dataset | Meir, P., Kruijt, B., Broadmeadow, M., Kull, O., Carswell, F. & Nobre, A. 2002 Acclimation of photosynthetic capacity to irradiance in tree canopies in relation to leaf nitrogen concentration and leaf mass per unit area. Plant, Cell and Environment. 25, 3, p. 343-357 15 p. |
| Whole Plant Hydraulic Conductance | MENCUCCINI M., 2003. The ecological significance of long distance water transport: short-term regulation and long-term acclimation across plant growth forms. Plant, Cell and Environment, 26:163-182. |
| Tropical dry forest species traits | Méndez-Toribio, M, Ibarra-Manríquez, G. Paz, H, and Lebrija-Trejos, H. (in press) Atmospheric and soil drought risks combined shape tree species assembly in a Tropical Dry Forest. Journal of Ecology |
| Chinese Traits | Meng, T.-T., Wang, H., Harrison, S. P., Prentice, I. C., Ni, J., and Wang, G.: Responses of leaf traits to climatic gradients: adaptive variation versus compositional shifts, Biogeosciences, 12, 5339-5352, https://doi.org/10.5194/bg-12-5339-2015, 2015. |
| Mont_Mégantic_Individual_Traits_2016-2017 | Messier J, C Violle, BJ Enquist, MJ Lechowicz & B, J McGill. 2018. Similarities and differences in intrapopulation trait correlations of co-occurring tree species: Consistent water use relationships amidst widely different correlation patterns. American Journal of Botany. 105(9): 1–14. (DOI: 10.1002/ajb2.1146) |
| Leaf and Whole Plant Traits Database | Meziane, D. and B. Shipley. 1999. Interacting components of interspecific relative growth rate: constancy and change under differing conditions of light and nutrient supply. Functional Ecology 13:611-622. |
| Leaf and Whole Plant Traits Database | Meziane, D. and B. Shipley. 1999. Interacting determinants of specific leaf area in 22 herbaceous species: effects of irradiance and nutrient availability. Plant Cell and Environment 22:447-459. |
| UV-B Radiation Sensitivity of Hieracium Pilosella | Michael Beckmann, Maria Hock, Helge Bruelheide, Alexandra Erfmeier (2012) The role of UV-B radiation in the invasion of Hieracium pilosella—A comparison of German and New Zealand plants. Environmental and Experimental Botany 75 173– 180 |
| Michaletz_RMBL2013 | Michaletz ST, Weiser MD, McDowell NG, Zhou J, Kaspari M, Helliker BR, Enquist BJ. 2016. The energetic and carbon economic origins of leaf thermoregulation. Nature Plants 2:16129 |
| Michaletz_RMBL2015 | Michaletz ST, Weiser MD, McDowell NG, Zhou J, Kaspari M, Helliker BR, Enquist BJ. 2016. The energetic and carbon economic origins of leaf thermoregulation. Nature Plants 2:16129 |
| Michaletz_SUMO2015 | Michaletz ST, Weiser MD, McDowell NG, Zhou J, Kaspari M, Helliker BR, Enquist BJ. 2016. The energetic and carbon economic origins of leaf thermoregulation. Nature Plants 2:16129 |
| Leaf traits for Picea glauca and Pinus sylvestris on University of Calgary (Canada) campus | Michaletz, S.T., and E.A. Johnson. 2006. A heat transfer model of crown scorch in forest fires. Canadian Journal of Forest Research 36(11): 2839-2851 |
| Leaf traits for Picea glauca and Pinus sylvestris on University of Calgary (Canada) campus | Michaletz, S.T., and E.A. Johnson. 2008. A biophysical process model of tree mortality in surface fires. Canadian Journal of Forest Research 28: 2013-2029 |
| Thermo-Mediterranean species along Greece | Michelaki C, Nikolaos M. Fyllas, Alexandros Galanidis, Maria Aloupi, Eleftherios Evangelou, Margarita Arianoutsou, Panayiotis G. Dimitrakopoulos, 2019, An integrated phenotypic trait-network in thermo-Mediterranean vegetation describing alternative, coexisting resource-use strategies, Science of The Total Environment, Volume 672, Pages 583-592, https://doi.org/10.1016/j.scitotenv.2019.04.030 |
| Altitudinal Vicariants Spain | Milla & Reich 2011 Annals of Botany 107: 455–465, 2011. |
| Ozark glade grassland plants | Miller JED, Ives AR, Harrison SP, Damschen EI (2017) Data from: Early- and late-flowering guilds respond differently to landscape spatial structure. Dryad Digital Repository. https://doi.org/10.5061/dryad.7fq07 |
| Ozark glade grassland plants | Miller JED, Ives AR, Harrison SP, Damschen EI (2018) Early‐ and late‐flowering guilds respond differently to landscape spatial structure. J Ecol. 106:1033–1045. https://doi.org/10.1111/1365-2745.12849 |
| Antibiotics-effects on plant traits | Minden V, Deloy A, Volkert AM, Leonhardt SD, Pufal G (2017) Antibiotics impact plant traits, even at small concentrations. Annals of Botany Plants 9:1-19. doi.org/10.1093/aobpla/plx010 |
| Trait-responses of Impatiens species to light and nutrients | Minden V, Gorschlüter J (2016) Comparison of native and non-native Impatiens species across experimental light and nutrient gradients. Plant Ecology and Evolution 149: 59-72. doi.org/10.5091/plecevo.2016.1118 |
| Traits of halophytic species | Minden V, M Kleyer (2015): Ecosystem multifunctionality of coastal marshes is determined by key plant traits, Journal of Vegetation Science 26: 651-662 |
| Element contents of plant organs of halohytic species, NW-Germany | Minden V, Michael Kleyer (2014): Internal and external regulation of plant organ stoichiometry, Plant Biology, 16: 897-907 |
| Plant traits along NPK gradients | Minden V, olde Venterink H (2019) Plant traits and species interactions along gradients of N, P and K availabilities. Functional Ecology, status 04-30-2019: manuscript accepted |
| Silicon response to nutrient limitation | Minden V, Schaller J, olde Venterink H (2020) Plants increase silicon content as a response to nitrogen or phosphorus limitation: a case study with Holcus lanatus. Plant and Soil. doi.org/10.1007/s11104-020-04667-1 |
| Antibiotics-effects on plant elements | Minden V, Schnetger B, Pufal G, Leonhardt SD (2018) Antibiotic-induced effects on scaling relationships and on plant element contents in herbs and grasses. Ecology and Evolution 8: 6699-6713. doi.org/10.1002/ece3.4168 |
| Traits of Halohytic Species in North-West-Germany | Minden Vanessa, Michael Kleyer (2011): Testing the effect–response framework: key response and effect traits determining above-ground biomass of salt marshes. Journal of Vegetation Science 22: 387-401 |
| Traits of Halohytic Species in North-West-Germany | Minden Vanessa, Sandra Andratschke, Janina Spalke, Hanna Timmermann, Michael Kleyer (2012): Plant trait–environment relationships in salt marshes: Deviations from predictions by ecological concepts. Perspectives in Plant Ecology, Evolution and Systematics, 14: 183-192 |
| Saskatchewan Plant Trait Database | Mischkolz, J. M. (2013) Selecting and evaluating native forage mixtures for the mixed grass prairie. Master Thesis, University of Saskatchewan, Saskatoon, SK. http://hdl.handle.net/10388/ETD-2013-04-988 |
| Global Seed Mass, Plant Height Database | Moles, A. T., D. D. Ackerly, C. O. Webb, J. C. Tweddle, J. B. Dickie, A. J. Pitman, and M. Westoby. 2005. Factors that shape seed mass evolution. Proceedings of the National Academy of Sciences of the United States of America 102:10540-10544. |
| Global Seed Mass, Plant Height Database | Moles, A. T., D. S. Falster, M. R. Leishman, and M. Westoby. 2004. Small-seeded species produce more seeds per square metre of canopy per year, but not per individual per lifetime. Journal of Ecology 92:384-396. |
| Global Seed Mass, Plant Height Database | Moles, A.T., Warton, D.I., Warman, L., Swenson, N.G., Laffan, S.W., Zanne, A.E., Pitman, A., Hemmings, F.A. & Leishman, M.R. 2009 Global patterns in plant height. J Ecol 97, 923-932. |
| Hokkaido leaf traits | Mori, A. S., Shiono, T., Haraguchi, T. F., Ota, A. T., Koide, D., Ohgue, T., Kitagawa, R., Maeshiro, R., Aung, T. T., Nakamori, T., Hagiwara, Y., Matsuoka, S., Ikeda, A., Hishi, T., Hobara, S., Mizumachi, E., Frisch, A., Thor, G., Fujii, S., Osono, T. and Gustafsson, L. (2015), Functional redundancy of multiple forest taxa along an elevational gradient: predicting the consequences of non-random species loss. J. Biogeogr., 42: 1383–1396. doi:10.1111/jbi.12514 |
| Traits of Plants in Canada (TOPIC) | Morris, D.M. 2014. Tree growth response data set from Ontario’s black spruce long-term soil productivity (LTSP) study sites. Ont. Min. Nat. Resourc., Centre for Northern Forest Ecosystem Research. |
| Traits of Plants in Canada (TOPIC) | Morrison, I & Wickware G.M. Internal government report: project P-380 in Richardson K. (Ed.) (1990) EnFor (Energy from the Forest): Review. Forestry Canada. 45 pages |
| Leaf traits related to mesophyll conductance in wild relatives of tomato (Solanum lycopersicon) | Muir CD, RP Hangarter, LC Moyle & PA Davis (2014) Morphological and anatomical determinants of mesophyll conductance in wild relatives of tomato (Solanum sect. Lycopersicon, sect. Lycopersicoides; Solanaceae) Plant Cell Environment 37:1415–1426 doi:10.1111/pce.12245 |
| ECOQUA South American Plant Traits Database | Muller, S. C., G. E. Overbeck, J. Pfadenhauer, and V. D. Pillar. 2007. Plant functional types of woody species related to fire disturbance in forest-grassland ecotones. Plant Ecology 189:1-14. |
| The Photosynthetic Pathways of Plant Species surveyed in Australia at TERN Ecosystem Surveillance Pl | Munroe,S., McInerney,F., Andrae,J., Welti,N., Guerin,G., Leitch,E., Hall,T., Szarvas,S., Atkins,R., Caddy-Retalic,S., Sparrow,B. (2020): The Photosynthetic Pathways of Plant Species surveyed in TERN Ecosystem Surveillance Plots. Version 1.0.0. Terrestrial Ecosystem Research Network (TERN). dataset. https://doi.org/10.25901/k61f-yz90 Munroe, Samantha, Francesca A. McInerney, Jake Andrae, Nina Welti, Greg Guerin, Emrys Leitch, Tony Hall, et al. 2020. “The Photosynthetic Pathways of Plant Species Surveyed in Australia’s National Terrestrial Monitoring Network.” EcoEvoRxiv. November 22. doi:10.32942/osf.io/xkycf. |
| Leaf and Whole-Plant Traits Database: Hydraulic and Gas Exchange Physiology, Anatomy, Venation Struc | Nakahashi, C. D., K. Frole, and L. Sack. 2005. Bacterial leaf nodule symbiosis in Ardisia (Myrsinaceae): Does it contribute to seedling growth capacity? Plant Biology 7:495-500. |
| Leaf Traits of Aquatic Plants | Nekrasova G.F., Ronzhina D.A., Maleva M.G., P’yankov V.I. Photosynthetic Metabolism and Activity of Carboxylating Enzymes in Emergent, Floating, and Submersed Leaves of Hydrophytes // Russian Journal of Plant Physiology. 2003. V. 50. No. 1. P. 57–67. DOI: 10.1023/A:1021936418052 |
| Seed trait data from Neuschulz et al. 2016 Sci REp. | Neuschulz, E. L., T. Mueller, M. Schleuning & K. Böhning-Gaese (2016) Pollination and seed dispersal are the most threatened processes of plant regeneration. Scientific Reports 6, Article number: 29839 |
| Global Leaf Robustness and Physiology Database | Niinemets, U. 1999. Components of leaf dry mass per area - thickness and density - alter leaf photosynthetic capacity in reverse directions in woody plants. New Phytologist 144:35-47. |
| Global Leaf Robustness and Physiology Database | Niinemets, U. 2001. Global-scale climatic controls of leaf dry mass per area, density, and thickness in trees and shrubs. Ecology 82:453-469. |
| Tree Tolerance Database | Niinemets, U. and F. Valladares. 2006. Tolerance to shade, drought, and waterlogging of temperate Northern Hemisphere trees and shrubs. Ecological Monographs 76:521-547. |
| Mycorrhiza Database | Nina Koele, Ian A. Dickie, Jacek Oleksyn, Sarah J. Richardson and Peter B. Reich (2012) No globally consistent effect of ectomycorrhizal status on foliar traits. New Phytologist doi: 10.1111/j.1469-8137.2012.04297.x |
| Ti Tree | Nolan, R.H., Fairweather, K.A., Tarin, T., Santini, N.S., Cleverly, J., Faux, R. & Eamus, D. 2017, Divergence in plant water-use strategies in semiarid woody species, Functional Plant Biology, vol. 44, no. 11, pp. 1134-1146 |
| Poblet Ecophysiogology | Nolan, R.H., Hedo, J., Arteaga, C., Sugai, T. & Resco de Dios, V. (2018) Physiological drought responses improve predictions of live fuel moisture dynamics in a Mediterranean forest. Agricultural and Forest Meteorology, 263, 417-427 |
| Ti Tree | Nolan, R.H., Tarin, T., Fairweather, K.A., Cleverly, J. and Eamus, D., 2017. Variation in photosynthetic traits related to access to water in semiarid Australian woody species. Funct. Plant Biol., 44(11): 1087-1097 |
| Traits of urban species from Ibagué Colombia | Núñez-Florez, R., U. Pérez-Gómez, and F. Fernández-Méndez. 2019. Functional diversity criteria for selecting urban trees. Urban Forestry & Urban Greening 38:251–266. https://doi.org/10.1016/j.ufug.2019.01.005 |
| Catalonian Mediterranean Forest Trait Database | Ogaya, R. and J. Penuelas. 2003. Comparative field study of Quercus ilex and Phillyrea latifolia: photosynthetic response to experimental drought conditions. Environmental and Experimental Botany 50:137-148. |
| Catalonian Mediterranean Forest Trait Database | Ogaya, R. and J. Penuelas. 2006. Contrasting foliar responses to drought in Quercus ilex and Phillyrea latifolia. Biologia Plantarum 50:373-382. |
| Catalonian Mediterranean Forest Trait Database | Ogaya, R. and J. Penuelas. 2007. Tree growth, mortality, and above-ground biomass accumulation in a holm oak forest under a five-year experimental field drought. Plant Ecology 189:291-299. |
| Catalonian Mediterranean Forest Trait Database | Ogaya, R. and J. Penuelas. 2008. Changes in leaf delta C-13 and delta N-15 for three Mediterranean tree species in relation to soil water availability. Acta Oecologica-International Journal of Ecology 34:331-338. |
| Olson et al. 2020 Ecological Monographs vessel diameter-plant height scaling | Olson ME, Rosell JA, Martínez-Pérez C, León-Gómez C, Fajardo A, Isnard S, Cervantes-Alcayde M-A, Echeverría A, Figueroa-Abúndiz, Segovia-Rivas A, et al. in press. Xylem vessel diameter-shoot length scaling: ecological significance of porosity types and other traits. Ecological Monographs |
| Onoda 2017 leaf dataset | Onoda Y, Wright IJ, Evans JR, Hikosaka K, Kitajima K, Niinemets Ü, Poorter H, Tosesns T, Westoby M. (2017) Physiological and structural tradeoffs underlying the leaf economics spectrum. New Phytologist |
| Leaf Biomechanics Database | Onoda, Y., M. Westoby, P. B. Adler, A. M. F. Choong, F. J. Clissold, J. H. C. Cornelissen, S. Diaz, N. J. Dominy, A. Elgart, L. Enrico, P. V. A. Fine, J. J. Howard, A. Jalili, K. Kitajima, H. Kurokawa, C. McArthur, P. W. Lucas, L. Markesteijn, N. Perez-Harguindeguy, L. Poorter, L. Richards, L. S. Santiago, Jr. E. Sosinski, S. Van Bael, D. I. Warton, I. J. Wright, S. J. Wright, and N. Yamashita. 2011 . Global patterns of leaf mechanical properties. Ecology Letters 14:301-312. |
| Plant Traits from Fynbos Forests in the Cape Region | Onstein RE, Richard J. Carter, Yaowu Xing, H. Peter LinderInstitute (2014) Diversification rate shifts in the Cape Floristic Region: The right traits in the right place at the right time. Perspectives in Plant Ecology, Evolution and Systematics 16(6) 331–340 DOI:10.1016/j.ppees.2014.08.002 |
| Annonaceae_traits | Onstein, R.E., Kissling, W.D., Chatrou, L.W., Couvreur, T.L.P., Morlon, H., Sauquet, H. (2019) Which frugivory‐related traits facilitated historical long‐distance dispersal in the custard apple family (Annonaceae)? Journal of Biogeography 46: 1874– 1888 |
| Rhamnaceae_traits | Onstein, R.E., Linder, H.P. (2016). Beyond climate: convergence in fast evolving sclerophylls in Cape and Australian Rhamnaceae predates the mediterranean climate. Journal of Ecology 104: 665-677 |
| The Netherlands Plant Traits Database | Ordonez, J. C., P. M. van Bodegom, J. P. M. Witte, R. P. Bartholomeus, H. F. van Dobben, and R. Aerts. 2010. Leaf habit and woodiness regulate different leaf economy traits at a given nutrient supply. Ecology 91:3218-3228. |
| The Netherlands Plant Traits Database | Ordonez, J. C., P. M. van Bodegom, J. P. M. Witte, R. P. Bartholomeus, J. R. van Hal, and R. Aerts. 2010. Plant Strategies in Relation to Resource Supply in Mesic to Wet Environments: Does Theory Mirror Nature? American Naturalist 175:225-239. |
| Plant traits of granite outcrops’ vegetation of Southwestern Australia | Ottaviani G (2015) Using a plant functional trait approach to determine dynamics of plant community assembly on granite outcrops of southwest Western Australia. – PhD dissertation |
| Plant traits of granite outcrops’ vegetation of Southwestern Australia | Ottaviani G, Keppel G, Marcantonio M, Mucina L, Wardell‐Johnson G (2019) Woody species in resource‐rich microrefugia of granite outcrops display unique functional signatures. Aust Ecol, 44: 575-580 |
| Plant traits of granite outcrops’ vegetation of Southwestern Australia | Ottaviani G, Marcantonio M, Mucina L (2016) Soil depth shapes plant functional diversity in granite outcrops vegetation of Southwestern Australia. Plant Ecol Divers, 9: 263-276 |
| Plant traits of granite outcrops’ vegetation of Southwestern Australia | Ottaviani G, Tsakalos JL, Keppel G, Mucina L (2018) Quantifying the effects of ecological constraints on trait expression using novel trait‐gradient analysis parameters. Ecol Evol, 8: 435–440 |
| BiolFlor Database | Otto, B. (2002): Merkmale von Samen, Früchten, generativen Germinulen und generativen Diasporen. In: Klotz, S., Kühn, I. & Durka, W. [eds.]: BIOLFLOR - Eine Datenbank zu biologisch-ökologischen Merkmalen der Gefäßpflanzen in Deutschland. - Schriftenreihe für Vegetationskunde 38. Bundesamt für Naturschutz, Bonn. |
| ECOQUA South American Plant Traits Database | Overbeck, G. E. 2005. Effect of fire on vegetation dynamics and plant types in subtropical grassland in southern Brazil. Department für Ökologie. PhD Thesis Technische Universität München, Freising, 139 pages. |
| ECOQUA South American Plant Traits Database | Overbeck, G. E. and J. Pfadenhauer. 2007. Adaptive strategies in burned subtropical grassland in southern Brazil. Flora 202:27-49. |
| Pinnacle Reserve, ACT | O’Reilly‐Nugent, A, Wandrag, EM, Catford, JA, Gruber, B, Driscoll, D, Duncan, RP. Measuring competitive impact: Joint‐species modelling of invaded plant communities. J Ecol. 2019; 00: 1– 11. https://doi.org/10.1111/1365-2745.13280 |
| Impatiens Glandulifera Dataset | Pahl, A.T., Kollmann, J., Mayer, A. & Haider, S. (2013): No evidence for local adaptation in an invasive alien plant: field and greenhouse experiments tracing a colonization sequence. Annals of Botany 112 (9): 1921-1930. DOI: 10.1093/aob/mct246 |
| Functional Traits of Trees | Paine CET, Amissah L, Auge H, Baraloto C, Baruffol M, … Hector A (2015) Globally, functional traits are weak predictors of juvenile tree growth, and we do not know why. Journal of Ecology, 103, 978–989. DOI: 10.1111/1365-2745.12401 |
| The VISTA Plant Trait Database | Pakeman, R. J., E. Garnier, S. Lavorel, P. Ansquer, H. Castro, P. Cruz, J. Dolezal, O. Eriksson, H. Freitas, C. Golodets, J. Kigel, M. Kleyer, J. Leps, T. Meier, M. Papadimitriou, V. P. Papanastasis, H. Quested, F. Quetier, G. Rusch, M. Sternberg, J. P. Theau, A. Thebault, and D. Vile. 2008. Impact of abundance weighting on the response of seed traits to climate and land use. Journal of Ecology 96:355-366. |
| The VISTA Plant Trait Database | Pakeman, R. J., J. Leps, M. Kleyer, S. Lavorel, E. Garnier, and V. Consortium. 2009. Relative climatic, edaphic and management controls of plant functional trait signatures. Journal of Vegetation Science 20:148-159. |
| Balruddery barley-mixture weed experiment, 2016. | PAKEMAN, R.J., BROOKER, R.W., KARLEY, A.J., NEWTON, A.C., MITCHELL, C., HEWISON, R.L., POLLENUS, J., GUY, D.C. & SCHÖB, C. (2020) Increased crop diversity reduces the functional space available for weeds. Weed Research, 60, 121-131. |
| Ukraine Wetlands Plant Traits Database | Pan, Y, Cieraad, E, van Bodegom, PM. Are ecophysiological adaptive traits decoupled from leaf economics traits in wetlands? Funct Ecol. 2019; 33: 1202– 1210. https://doi.org/10.1111/1365-2435.13329 |
| Weiqi-Sardans-Penuelas-China-plants | Papers Wang, Sardans, Penuelas on China plants (see http://globalecology.creaf.cat/ publications page |
| Tene_Traits | Patacca M, Hérault B, Zo-Bi IC, 2019 - Bark, Leaf and Root traits of tropical trees from the semi-deciduous forests of TENE, West Africa |
| The RAINFOR Plant Trait Database | Patiño, S., J. Lloyd, R. Paiva, T. R. Baker, C. A. Quesada, L. M. Mercado, J. Schmerler, M. Schwarz, A. J. B. Santos, A. Aguilar, C. I. Czimczik, J. Gallo, V. Horna, E. J. Hoyos, E. M. Jimenez, W. Palomino, J. Peacock, A. Peña-Cruz, C. Sarmiento, A. Sota, J. D. Turriago, B. Villanueva, P. Vitzthum, E. Alvarez, L. Arroyo, C. Baraloto, D. Bonal, J. Chave, A. C. L. Costa, R. Herrera, N. Higuchi, T. Killeen, E. Leal, F. Luizão, P. Meir, A. Monteagudo, D. Neil, P. Núñez-Vargas, M. C. Peñuela, N. Pitman, N. Priante Filho, A. Prieto, S. N. Panfil, A. Rudas, R. Salomão, N. Silva, M. Silveira, S. Soares deAlmeida, A. Torres-Lezama, R. Vásquez-Martínez, I. Vieira, Malhi, Y., , and O. L. Phillips. 2009. Branch xylem density variations across the Amazon Basin. Biogeosciences 6:545-568. |
| BBB - A global Belowground Bud Bank database | Pausas, J. G., Lamont, B. B., Paula, S. , Appezzato‐da‐Glória, B. and Fidelis, A. (2018), Unearthing belowground bud banks in fire‐prone ecosystems. New Phytol, 217: 1435-1448. doi:10.1111/nph.14982 |
| Plant Traits of Acidic Grasslands in Central Spain | Peco B., de Pablos I., Traba J. , & Levassor C. (2005) The effect of grazing abandonment on species composition and functional traits: the case of dehesa Basic and Applied Ecology, 6(2): 175-183 |
| Hawaiian Leaf Traits Database | Penuelas, J., J. Sardans, J. Llusia, S. Owen, J. Carnicer, T. W. Giambelluca, E. L. Rezende, M. Waite, and Ü. Niinemets. 2010. Faster returns on "leaf economics" and different biogeochemical niche in invasive compared with native plant species. Global Change Biology 16:2171-2185. |
| Hawaiian Leaf Traits Database | Penuelas, J., J. Sardans, J. Llusia, S. Owen, J. Silva, and Ü. Niinemets. 2010. Higher allocation to low cost chemical defenses in invasive species of Hawaii. Journal of Chemical Ecology 36:1255-1270. |
| Garraf-Penuelas | Peñuelas, J., Sardans, J., Filella, I., Estiarte, M., Llusià, J., Ogaya, R., Carnicer, J., Bartrons, M., Rivas-Ubach, A., Grau, O., Peguero, G., Margalef, O., Pla-Rabés, S., Stefanescu, C., Asensio, L., Preece, C., Liu, L., Verger, A., Rico, L., Barbeta, A., Achotegui-Castells, a., Gargallo-Garriga, a., Sperlich, D., Farré-Armengol, G., Fernández-Martínez, M., Liu, D., Zhang, C., Urbina, I., Camino, C., Vives, M., Nadal-Sala, D., Sabaté, S., Gracia, C., Terradas, J. 2018. Assessment of the impacts of climate change on Mediterranean terrestrial ecosystems based on data from field experiments and long-term monitored field gradients in Catalonia. Environmental and Experimental Botany, 152 (2018) 49–59. doi: 10.1016/j.envexpbot.2017.05.012 |
| Prades-Penuelas | Peñuelas, J., Sardans, J., Filella, I., Estiarte, M., Llusià, J., Ogaya, R., Carnicer, J., Bartrons, M., Rivas-Ubach, A., Grau, O., Peguero, G., Margalef, O., Pla-Rabés, S., Stefanescu, C., Asensio, L., Preece, C., Liu, L., Verger, A., Rico, L., Barbeta, A., Achotegui-Castells, a., Gargallo-Garriga, a., Sperlich, D., Farré-Armengol, G., Fernández-Martínez, M., Liu, D., Zhang, C., Urbina, I., Camino, C., Vives, M., Nadal-Sala, D., Sabaté, S., Gracia, C., Terradas, J. 2018. Assessment of the impacts of climate change on Mediterranean terrestrial ecosystems based on data from field experiments and long-term monitored field gradients in Catalonia. Environmental and Experimental Botany, 152 (2018) 49–59. doi: 10.1016/j.envexpbot.2017.05.012 |
| Mediterranean mixed forest | Perea, A. J., Garrido, J. L., & Alcántara, J. M. (2021). Plant functional traits involved in the assembly of canopy–recruit interactions. Journal of Vegetation Science, 32(1), e12991 |
| Reliable herbarium-derived proxies for three functional traits | Perez, T. M., Rodriguez, J., and Heberling, J. M. (2020) Herbarium-based measurements reliably estimate three functional traits. American Journal of Botany 107( 10): 1457– 1464. |
| Ecotron Species Composition and Global Change Experiment | Pete Manning, John E. Newington, Helen R. Robson, Mark Saunders, Till Eggers, Mark A. Bradford,3 Richard D. Bardgett, Michael Bonkowski, Richard J. Ellis, Alan C. Gange, Susan J. Grayston, Ellen Kandeler, Sven Marhan, Eileen Reid, Dagmar Tscherko, H. Charles J. Godfray and Mark Rees (2006) Decoupling the direct and indirect effects of nitrogen deposition on ecosystem function. Ecology Letters 9: 1015–1024 doi: 10.1111/j.1461 0248.2006.00959.x |
| San Lorenzo Epiphyte Leaf Traits Database | Petter G, Wagner K, Zotz G, Cabral JS, Wanek W, Sanchez Delgado EJ, Kreft H. 2016. Distribution of functional leaf traits of vascular epiphytes: vertical trends, intra- and interspecific trait variability, and phylogenetic signals. Functional Ecology, 30: 188–198. |
| Hydrophytes Traits Database | Pierce S., Brusa G., Sartori M. & Cerabolini B.E.L. 2012. Combined use of leaf size and economics traits allows direct comparison of hydrophyte and terrestrial herbaceous adaptive strategies. Annals of Botany 109(5): 1047-1053 |
| Leaf Structure and Economics Spectrum | Pierce S., Brusa G., Vagge I., Cerabolini B.E.L. (2013) Allocating CSR plant functional types: the use of leaf economics and size traits to classify woody and herbaceous vascular plants. Functional Ecology, 27(4): 1002-1010 |
| Leaf Structure and Economics Spectrum | Pierce S., Ceriani R.M., De Andreis R., Luzzaro A. & Cerabolini B. 2007. The leaf economics spectrum of Poaceae reflects variation in survival strategies. Plant Biosystems 141(3): 337-343. |
| Leaf Structure and Economics Spectrum | Pierce S., Luzzaro A., Caccianiga M., Ceriani R.M. & Cerabolini B. 2007. Disturbance is the principal ?-scale filter determining niche differentiation, coexistence and biodiversity in an alpine community. Journal of Ecology 95: 698-706. |
| Olive Lawn Orchid Trait Database (OLO) | Pierce S., Vagge I., Brusa G., Cerabolini B.E.L. (2014) The intimacy between sexual traits and Grime’s CSR strategies for orchids coexisting in semi-natural calcareous grassland at the Olive Lawn. Plant Ecology, 215(5): 495-505 |
| ECOQUA South American Plant Traits Database | Pillar, V. D. and E. E. Sosinski. 2003. An improved method for searching plant functional types by numerical analysis. Journal of Vegetation Science 14:323-332. |
| FAPESP Brazil Rainforest Database | Pillar, V. D. and E. E. Sosinski. 2003. An improved method for searching plant functional types by numerical analysis. Journal of Vegetation Science 14:323-332. |
| Dataset of leaf inclination angles for 71 different Eucalyptus species | Pisek, J., Adamson, K. (2020). A dataset of leaf inclination angles for 71 different Eucalyptus species. Data in Brief. Data in Brief 33, 106391, doi: 10.1016/j.dib.2020.106391 |
| Leaf angles | Pisek, J., Sonnentag, O., Richardson, A.D., Mõttus, M. (2013). Is the spherical leaf inclination angle distribution a valid assumption for temperate and boreal broadleaf tree species? Agricultural and Forest Meteorology, 169, 186 - 194 |
| Ecophysiological parameters of tree and shrub leaves in forest-steppe plantings | Pomogaybin A.V., Pomogaybin Ye.A. K izucheniyu bioekologicheskikh osobennostey predstaviteley roda Juglans L. pri introduktsii v lesostepi Srednego Povolzhya // Sovremennaya botanika v Rossii. Trudy XIII syezda Russkogo botanicheskogo obshchestva T. P.156-158 |
| The Tansley Review LMA Database | Poorter, H., U. Niinemets, L. Poorter, I. J. Wright, and R. Villar. 2009. Causes and consequences of variation in leaf mass per area (LMA): a meta-analysis. New Phytologist 182:565-588. |
| Tropical Rainforest Traits Database | Poorter, L. 2009. Leaf traits show different relationships with shade tolerance in moist versus dry tropical forests. New Phytologist 181:890-900. |
| Tropical Rainforest Traits Database | Poorter, L. and F. Bongers. 2006. Leaf traits are good predictors of plant performance across 53 rain forest species. Ecology 87:1733-1743. |
| BIOPOP: Functional Traits for Nature Conservation | Poschlod, P., M. Kleyer, A. K. Jackel, A. Dannemann, and O. Tackenberg. 2003. BIOPOP - a database of plant traits and Internet application for nature conservation. Folia Geobotanica 38:263-271. |
| Chinese Traits | Prentice, I.C., Meng, T., Wang, H., Harrison, S.P., Ni, J., Wang, G., 2011. Evidence for a universal scaling relationship of leaf CO2 drawdown along a moisture gradient. New Phytologist 190: 169–180 |
| Jasper Ridge Californian Woody Plants Database | Preston, K. A., W. K. Cornwell, and J. L. DeNoyer. 2006. Wood density and vessel traits as distinct correlates of ecological strategy in 51 California coast range angiosperms. New Phytologist 170:807-818. |
| Leaf Allometry Dataset | Price, C.A. and B.J. Enquist. Scaling of mass and morphology in Dicotyledonous leaves: an extension of the WBE model. (2007) Ecology 88(5): 1132–1141. |
| Leaf Allometry Dataset | Price, C.A., B.J. Enquist and V.M. Savage. A general model for allometric covariation in botanical form and function. (2007) Proceedings of the National Academy of Sciences 104 (32): 13204-13209. |
| Functional traits of Cistus species leaf cohorts | Puglielli G., Varone L. 2018. Inherent variation of functional traits in winter and summer leaves of Mediterranean seasonal dimorphic species: evidence of a ‘within leaf cohort’ spectrum10: ply027; doi: 10.1093/aobpla/ply027 |
| NZ kettehole plant traits | Purcell, A. S. T., W. G. Lee, A. J. Tanentzap, and D. C. Laughlin. 2019. Fine root traits are correlated with flooding duration while aboveground traits are related to grazing in an ephemeral wetland. Wetlands 39(2): 291-302. |
| Leaf and Whole Plant Traits Database | Pyankov, V. I., A. V. Kondratchuk, and B. Shipley. 1999. Leaf structure and specific leaf mass: the alpine desert plants of the Eastern Pamirs, Tadjikistan. New Phytologist 143:131-142. |
| Leaf and Whole-Plant Traits Database: Hydraulic and Gas Exchange Physiology, Anatomy, Venation Struc | Quero, J. L., R. Villar, T. Maranon, R. Zamora, D. Vega, and L. Sack. 2008. Relating leaf photosynthetic rate to whole-plant growth: drought and shade effects on seedlings of four Quercus species. Functional Plant Biology 35:725-737. |
| Abisko & Sheffield Database | Quested, H. M., J. H. C. Cornelissen, M. C. Press, T. V. Callaghan, R. Aerts, F. Trosien, P. Riemann, D. Gwynn-Jones, A. Kondratchuk, and S. E. Jonasson. 2003. Decomposition of sub-arctic plants with differing nitrogen economies: A functional role for hemiparasites. Ecology 84:3209-3221. |
| FruitTraits_Ecuador_NeuschulzSchleuningBoeGae | Quitián et al. 2019 Functional Ecology, 33: 399-410 |
| FruitTraits_Ecuador_NeuschulzSchleuningBoeGae | Quitián et al. 2019 Oecologia, 189: 435–445 |
| Leaf angles Raabe et al 2015 | Raabe, K., Pisek, J., Sonnentag, O., Annuk, K. (2015). Variations of leaf inclination angle distribution with height over the growing season and light exposure for eight broadleaf tree species. Agricultural and Forest Meteorology, 214-215, 2-11 |
| BolivianBofedalTraitData | Raevel, V., Anthelme, F., Meneses, R. I., & Munoz, F. (2018). Cushion-plant protection determines guild-dependent plant strategies in high-elevation peatlands of the Cordillera Real, Bolivian Andes. Perspectives in Plant Ecology, Evolution and Systematics, 30, 103-114 |
| MediterraneanRoadcutTraitData | Raevel, V., Violle, C., & Munoz, F. (2012). Mechanisms of ecological succession: insights from plant functional strategies. Oikos, 121(11): 1761–1770. doi:10.1111/j.1600-0706.2012.20261.x Raevel, V., Munoz, F., Pons, V., Renaux, A., Martin, A., & Thompson, J.D. (2013). Changing assembly processes during a primary succession of plant communities on Mediterranean roadcuts. Journal of Plant Ecology, 6(1):19-28. https://doi.org/10.1093/jpe/rts011 |
| Reich-Oleksyn Global Leaf N, P Database | Reich, P. B., J. Oleksyn, and I. J. Wright. 2009. Leaf phosphorus influences the photosynthesis-nitrogen relation: a cross-biome analysis of 314 species. Oecologia 160:207-212. |
| Global A, N, P, SLA Database | Reich, P. B., J. Oleksyn, and I. J. Wright. 2009. Leaf phosphorus influences the photosynthesis-nitrogen relation: a cross-biome analysis of 314 species. Oecologia 160:207-212. |
| Global Respiration Database | Reich, P. B., M. G. Tjoelker, K. S. Pregitzer, I. J. Wright, J. Oleksyn, and J. L. Machado. 2008. Scaling of respiration to nitrogen in leaves, stems and roots of higher land plants. Ecology Letters 11:793-801. |
| Vegetation states from intensively monitored crop sites in western Germany | Reichenau, T. G., Korres, W., Schmidt, M., Graf, A., Welp, G., Meyer, N., Stadler, A., Brogi, C., Schneider, K. (2020) A comprehensive dataset of vegetation states, fluxes of matter and energy, weather, agricultural management, and soil properties from intensively monitored crop sites in western Germany. Earth System Sciences Data, 12, doi:10.5194/essd-12-2333-2020 |
| New Zealand Nothofagus leaf and stem traits | Richardson SJ, Allen RB, Buxton RP, Easdale TA, Hurst JM, Morse CWM, Smissen RD, Peltzer DA. 2013. Intraspecific Relationships among Wood Density, Leaf Structural Traits and Environment in Four Co-Occurring Species of Nothofagus in New Zealand. PLoS ONE 8(3): e58878. doi:10.1371/journal.pone.0058878 |
| New Zealand Alpine Granite Leaf Nutrient Concentrations | Richardson SJ, Williams PA, Mason NWH, Buxton RP, Courtney SP, Rance BD, Clarkson BR, Hoare RJB, StJohn MG, Wiser SK. 2012. Rare species drive local trait diversity in two geographically disjunct examples of a naturally-rare alpine ecosystem in New Zealand. Journal of Vegetation Science 23: 626-639. doi: 10.1111/j.1654-1103.2012.01396.x |
| leaf and flower pressure volume curve data | Roddy, A. B. and Jiang, G.-F., and Cao, K.-F. and Simonin, K. A. and Brodersen, C. R. 2019. Hydraulic traits are more diverse in flowers than in leaves. New Phytologist 223:193-203 |
| Plant Trait Dataset for Tree-Like Growth Forms | Rodrigues, A.V.; Bones, F.L.V.; Schneiders, A.; Oliveira, L.Z.; Vibrans, A.C.; Gasper, A.L. Plant Trait Dataset for Tree-Like Growth Forms Species of the Subtropical Atlantic Rain Forest in Brazil. Data 2018, 3, 16. |
| Leaf Mass Area, Leaf Carbon and Nitrogen Content, Barrow, Alaska | Rogers A, Serbin SP, Ely KS, Sloan VL, Wullschleger SD (2017) Terrestrial biosphere models underestimate photosynthetic capacity and CO2 assimilation in the Arctic. New Phytologist. doi: 10.1111/nph.14740 |
| Leaf nutrient concentrations | Rolo V., López-Díaz M. L. and Moreno G. (2012) Shrubs affect soil nutrients availability with contrasting consequences for pasture understory and tree overstory production and nutrient status in Mediterranean grazed open woodlands. Nutrient Cycling in Agroecosystems, 1–14 |
| Leaf nutrient concentrations | Rolo, V., Rivest, D., López-Díaz, M. L. and Moreno, G. (2015), Microhabitat effects on herbaceous nutrient concentrations at the community and species level in Mediterranean open woodlands: the role of species composition. Grass Forage Sci, 70: 219–228. doi:10.1111/gfs.12110 |
| Leaf Traits of Aquatic Plants | Ronzhina D.A., Ivanov L.A. Construction costs and mesostructure of leaves in hydrophytes // Russian Journal of Plant Physiology. 2014. Т. 61. № 6. P. 776-783. DOI: 10.1134/S102144371406017X |
| Leaf Traits of Aquatic Plants | Ronzhina D.A., Ivanov L.A., Lambers G., P’yankov V.I. Changes in Chemical Composition of Hydrophyte Leaves during Adaptation to Aquatic Environment // Russian Journal of Plant Physiology. 2009. V.56. No.3. P.355–362 |
| Leaf Traits of Aquatic Plants | Ronzhina D.A., Ivanov L.A., P’yankov V.I. Chemical Composition of Leaves and Structure of Photosynthetic Apparatus in Aquatic Higher Plants // Russian Journal of Plant Physiology. 2010. V.57. No.3. P.368–375 |
| Leaf Traits of Aquatic Plants | Ronzhina D.A., Nekrasova G.F., P’yankov V.I. Comparative Characterization of the Pigment Complex in Emergent, Floating, and Submerged Leaves of Hydrophytes // Russian Journal of Plant Physiology. 2004. V. 51. No. 1. P. 21–27. DOI: 10.1023/B:RUPP.0000011299.93961.8f |
| Leaf Traits of Aquatic Plants | Ronzhina D.A., P’yankov V.I. Structure of the Photosynthetic Apparatus in Leaves of Freshwater Hydrophytes: 1. General Characteristics of the Leaf Mesophyll and a Comparison with Terrestrial Plants // Russian Journal of Plant Physiology. 2001. V. 48. No. 5. P. 567–575. DOI: 10.1023/A:1016733015194 |
| Leaf Traits of Aquatic Plants | Ronzhina D.A., P’yankov V.I. Structure of the Photosynthetic Apparatus in Leaves of Freshwater Hydrophytes: 2. Quantitative Characterization of Leaf Mesophyll and the Functional Activity of Leaves with Different Degrees of Submersion // Russian Journal of Plant Physiology. 2001. V.8. No .6. P. 723–732. DOI: 10.1023/A:1012544105453 |
| Jena Experiment Traits | Roscher C, Schmid B, Buchmann N, Weigelt A, Schulze E-D (2011) Legume species differ in the responses of their functional traits to plant diversity. Oecologia 165, 437-452. |
| Jena Experiment Traits | Roscher C, Schumacher J, Lipowsky A, Gubsch M, Weigelt A, Schmid B, Buchmann N, Schulze E-D (2018) Functional groups differ in trait means, but not in trait plasticity to species richness in local grassland communities. Ecology 99, 2295-2307 |
| Dataset for Rosell et al. 2017 New Phytologist | Rosell, J. A., Olson, M. E., Anfodillo, T. and Martínez‐Méndez, N. (2017), Exploring the bark thickness–stem diameter relationship: clues from lianas, successive cambia, monocots and gymnosperms. New Phytol, 215: 569-581. doi:10.1111/nph.14628 |
| SwissNationalPark_Engadine | Rossi C., Kneubuehler M.,Schuetz M.,Shaepman M.E., Haller R.M.,Risch A.C. (2020) From local to regional: Functional diversity in differently managed alpine grasslands.Remote Sensing of Environment, Volume 236, 111415, DOI: 10.1016/j.rse.2019.111415 |
| SwissNationalPark_Engadine | Rossi,C., 2017. Three morphological plant traits in the Swiss National Park and surroundings. Swiss National Park data center MMD, doi: http://www.parcs.ch/snp/mmd_fullentry.php?docu_id=37905 |
| Seed Information Database, Royal Botanic Gardens, Kew | Royal Botanic Gardens Kew. (2019) Seed Information Database (SID). Version 7.1. Available from: http://data.kew.org/sid/ (06 2019) |
| KEW Seed Information Database (SID) | Royal Botanical Gardens KEW. 2008. Seed Information Database (SID). Version 7.1. Available from: http://data.kew.org/sid/ (May 2008). |
| KEW Seed Information Database (SID) | Royal Botanical Gardens KEW. 2008. Seed Information Database (SID). Version 7.1. Available from: http://data.kew.org/sid/ (May 2011). |
| Seed Information Database (SID) Seed Mass 2010 | Royal Botanical Gardens KEW. Seed Information Database (SID), http://data.kew.org/sid/ accessed May 2014 |
| Traits of Plants in Canada (TOPIC) | Royer-Tardif et al. 2018. "Fast-growing hybrids do not decrease understorey plant diversity compared to naturally regenerated forests and native plantations." Biodiversity and conservation 27.3: 607-631. |
| DISEQU-ALP | Rumpf S.B., Hülber K., Klonner G., Moser D., Schütz M., Wessely J., Willner W., Zimmermann N.E., Dullinger S. (2018) Range dynamics of mountain plants decrease with elevation. Proceedings of the National Academy of Sciences 115(8):1848-1853 |
| Leaf and Whole-Plant Traits Database: Hydraulic and Gas Exchange Physiology, Anatomy, Venation Struc | Sack, L. 2004. Responses of temperate woody seedlings to shade and drought: do trade-offs limit potential niche differentiation? Oikos 107:110-127. |
| Leaf and Whole-Plant Traits Database: Hydraulic and Gas Exchange Physiology, Anatomy, Venation Struc | Sack, L. and K. Frole. 2006. Leaf structural diversity is related to hydraulic capacity in tropical rain forest trees. Ecology 87:483-491. |
| Leaf and Whole-Plant Traits Database: Hydraulic and Gas Exchange Physiology, Anatomy, Venation Struc | Sack, L., M. T. Tyree, and N. M. Holbrook. 2005. Leaf hydraulic architecture correlates with regeneration irradiance in tropical rainforest trees. New Phytologist 167:403-413. |
| Leaf and Whole-Plant Traits Database: Hydraulic and Gas Exchange Physiology, Anatomy, Venation Struc | Sack, L., P. D. Cowan, N. Jaikumar, and N. M. Holbrook. 2003. The 'hydrology' of leaves: co-ordination of structure and function in temperate woody species. Plant Cell and Environment 26:1343-1356. |
| Leaf and Whole-Plant Traits Database: Hydraulic and Gas Exchange Physiology, Anatomy, Venation Struc | Sack, L., P. J. Melcher, W. H. Liu, E. Middleton, and T. Pardee. 2006. How strong is intracanopy leaf plasticity in temperate deciduous trees? American Journal of Botany 93:829-839. |
| Data for: Sancho-Knapik et al. Deciduous evergreen oaks contrasting responses in LMA | Sancho-Knapik, D., Escudero, A., Mediavilla, S., Scoffoni, C., Zailaa, J., Cavender-Bares, J., Álvarez-Arenas, T.G., Molins, A., Alonso-Forn, D., Ferrio, J.P., Peguero-Pina, J.J. and Gil-Pelegrín, E. (2021), Deciduous and evergreen oaks show contrasting adaptive responses in leaf mass per area across environments. New Phytol, 230: 521-534. https://doi.org/10.1111/nph.17151 |
| Plant Traits from Romania | Sanda V., Bita-Nicolae C.D. & Barabas N. (2003). The flora of spontane and cultivated cormophytes from Romania. Editura “Ion Borcea”, Bacau, 316 p (in Romanian) |
| California Coastal Grassland Database | Sandel, B., J. D. Corbin, and M. Krupa. 2011. Using plant functional traits to guide restoration: a case study in California coastal grassland. Ecosphere 2(2):art23. doi:10.1890/ES10-00175.1 |
| Catalonian Mediterranean Forest Trait Database | Sardans, J., J. Penuelas, and R. Ogaya. 2008. Drought-induced changes in C and N stoichiometry in a Quercus ilex Mediterranean forest. Forest Science 54:513-522. |
| Catalonian Mediterranean Forest Trait Database | Sardans, J., J. Penuelas, P. Prieto, and M. Estiarte. 2008. Changes in Ca, Fe, Mg, Mo, Na, and S content in a Mediterranean shrubland under warming and drought. Journal of Geophysical Research 113:doi:10.1029/2008JG000795. |
| Scalon bark traits | Scalon et al. Diversity of functional trade-offs enhances survival after fire in Neotropical savanna species. Journal of Vegetation Science |
| Leaf nutrient concentration_Scalon et al. 2017 | Scalon, M.C., Haridasan, M. & Franco, A.C. Plant Soil (2017). https://doi.org/10.1007/s11104-017-3437-0 |
| Biomass allocation in beech and spruce seedlings | Schall P, Lödige C, Beck M, Ammer C (2012) Biomass allocation to roots and shoots is more sensitive to shade and drought in European beech than in Norway spruce seedlings. Forest Ecology and Management 266: 246-253 |
| BIOTREE Trait Shade Experiment | Scherer-Lorenzen, M., Schulze, E.-D., Don, A., Schumacher, J. & Weller, E. (2007) Exploring the functional significance of forest diversity: A new long-term experiment with temperate tree species (BIOTREE). Perspectives in Plant Ecology, Evolution and Systematics, 9, 53-70. |
| Leaf element composition of ferns and lycophytes | Schmitt M, Mehltreter K., Sundue M., Testo W., Watanabe T., Jansen S. (2017) The evolution of aluminium accumulation in ferns and lycophytes. American Journal of Botany 104: 573-583 DOI: 10.3732/ajb.1600381 |
| Raja Ampat tree dataset | Schrader, J., Moeljono, S., Tambing, J., Sattler, C., Kreft, H. (2020) A new dataset on plant occurrences on small islands, including species abundances and functional traits across different spatial scales. Biodiversity Data Journal 8, e55275. doi: 10.3897/BDJ.8.e55275 |
| Traits of 59 grassland species | Schroeder-Georgi, T., Wirth, C., Nadrowski, K., Meyer, S. T., Mommer, L. and Weigelt, A. (2016), From pots to plots: hierarchical trait-based prediction of plant performance in a mesic grassland. J Ecol, 104: 206–218. doi:10.1111/1365-2745.12489 |
| branch_anatomy | Schuldt B, Leuschner C, Brock N, Horna V. 2013. Tree Physiology 33: 161-174, DOI: 10.1093/treephys/tps122 |
| branch_anatomy | Schumann K, Leuschner C, Schuldt B. 2019. Trees-Structure and Function, DOI: 10.1007/s00468-019-01874-x |
| FYNBASE - Database of Plant Traits From the South African Fynbos Biome | Schurr,F.M., Midgley,G.F., Rebelo,A.G., Reeves,G., Poschlod,P. & Higgins,S.I. (2007) Global Ecology and Biogeography, 16, 449-459. |
| The Xylem/Phloem Database | Schweingruber, F.H., Landolt, W.: The Xylem Database. Swiss Federal Research Institute WSL Updated (2005) |
| The Xylem/Phloem Database | Schweingruber, F.H., Poschlod, P. 2005: Growth rings in herbs and shrubs: Life span, age determination and stem anatomy. Forest, Snow and Landscape Res. 79, 195-415 |
| Leaf and Whole-Plant Traits Database: Hydraulic and Gas Exchange Physiology, Anatomy, Venation Struc | Scoffoni, C., A. Pou, K. Aasamaa, and L. Sack. 2008. The rapid light response of leaf hydraulic conductance: new evidence from two experimental methods. Plant Cell and Environment 31:1803-1812. |
| Catimbau National Park, Brazil | Sfair, J.C., de Bello, F., de França, T.Q., Baldauf, C., Tabarelli, M., 2018. Chronic human disturbance affects plant trait distribution in a seasonally dry tropical forest. Environmental Research Letters 13, 025005–025005. https://doi.org/10.1088/1748-9326/aa9f5e |
| Herbs Water Relations on Soil Moisture Gradients | Sheremetev S.N. (2005) Herbs on the soil moisture gradient (water relations and the structural-functional organization). KMK, Moscow, 271 pp. (In Russian) |
| Species Growth Forms (Angiosperms)-Update 9 | Sheremetiev S. N. and K. E. Chebotareva. Modern and Cretaceous–Cenozoic Diversification of Angiosperms. Biology Bulletin Reviews. September 2018, Volume 8, Issue 5, pp 351–374. 10.1134/S2079086418050079 |
| A Geological Age of an Angiosperm Genera and Families | Sheremetiev S. N. and K. E. Chebotareva. Modern and Cretaceous–Cenozoic Diversification of Angiosperms. Biology Bulletin Reviews. September 2018, Volume 8, Issue 5, pp 351–374. 10.1134/S2079086418050079 |
| Tropical Traits from West Java Database | Shiodera, S., J. S. Rahajoe, and T. Kohyama. 2008. Variation in longevity and traits of leaves among co-occurring understorey plants in a tropical montane forest. Journal of Tropical Ecology 24:121-133. |
| Leaf and Whole Plant Traits Database | Shipley B., 2002. Trade-offs between net assimilation rate and specific leaf area in determining relative growth rate: relationship with daily irradiance, Functional Ecology(16) 682-689 |
| Leaf and Whole Plant Traits Database | Shipley, B. 1989. The Use of above-Ground Maximum Relative Growth-Rate as an Accurate Predictor of Whole-Plant Maximum Relative Growth-Rate. Functional Ecology 3:771-775. |
| Leaf and Whole Plant Traits Database | Shipley, B. 1995. Structured Interspecific Determinants of Specific Leaf-Area in 34 Species of Herbaceous Angiosperms. Functional Ecology 9:312-319. |
| Leaf and Whole Plant Traits Database | Shipley, B. and M. J. Lechowicz. 2000. The functional co-ordination of leaf morphology, nitrogen concentration, and gas exchange in 40 wetland species. Ecoscience 7:183-194. |
| Leaf and Whole Plant Traits Database | Shipley, B. and M. Parent. 1991. Germination Responses of 64 Wetland Species in Relation to Seed Size, Minimum Time to Reproduction and Seedling Relative Growth-Rate. Functional Ecology 5:111-118. |
| Leaf and Whole Plant Traits Database | Shipley, B. and T. T. Vu. 2002. Dry matter content as a measure of dry matter concentration in plants and their parts. New Phytologist 153:359-364. |
| Traits of understory plants of western Canadian forest | Shovon, TA, Rozendaal, DMA, Gagnon, D, Gendron, F, Vetter, M, Vanderwel, MC. Plant communities on nitrogen‐rich soil are less sensitive to soil moisture than plant communities on nitrogen‐poor soil. J Ecol. 2019; 00: 1– 12. https://doi.org/10.1111/1365-2745.13251 |
| Dahra_Senegal_Sibret et al | Sibret T, Verbruggen W, Peaucelle M, Verryckt LT, Bauters M, Combe M, Boeckx P, Verbeeck H. High photosynthetic capacity of Sahelian C3 and C4 plants. Photosynth Res. 2021 Feb;147(2):161-175. doi: 10.1007/s11120-020-00801-3. Epub 2021 Jan 2. PMID: 33387194. |
| Div_Resource Pot Experiment | Siebenkäs A, Schumacher J, Roscher C (2015) Phenotypic plasticity to light and nutrient availability alters functional trait ranking across eight perennial grassland species. AoB Plants 7:plv029. doi: 10.1093/aobpla/plv029 |
| New York Old Field Plant Traits Database | Siefert, A. 2012. Spatial patterns of functional divergence in old-field plant communities. Oikos 121: 907-914 |
| Eastern US Old Field Plant Traits Database | Siefert, A., Fridley, J.D., and Ritchie, M.E. 2014. Community functional responses to soil and climate at multiple spatial scales: when does intraspecific variation matter? PLOS ONE 9: e111189 |
| Old fields of Eastern US (Siefert Data) | Siefert, A., Fridley, J.D., and Ritchie, M.E. 2014. Community functional responses to soil and climate at multiple spatial scales: when does intraspecific variation matter? PLOS ONE 9: e111189 |
| Leaf and stem traits of Eremanthus erythropappus | Silva M.C., Grazielle S.TeodorobEvelyn F.A.BragionaEduardovan den Berg, The role of intraspecific trait variation in the occupation of sharp forest-savanna ecotones, Flora Volume 253, April 2019, Pages 35-42 |
| Silva el al. 2019 | Silva V, Filipe X. Catry, Paulo M. Fernandes, Francisco C. Rego, Paula Paes, Leónia Nunes, Ana D. Caperta, Cecília Sérgio, et al. Effects of grazing on plant composition, conservation status and ecosystem services of Natura 2000 shrub-grassland habitat typ Biodivers Conserv (2019) 28:1205-1224 DOI 10.1007/s10531-019-01718-7 |
| Silva et al., 2020 | Silva, V., Catry, F.X, Fernandes, P.M, Rego, F.C., Bugalho, M.N. 2020, Trade-offs between fire hazard reduction and conservation in a Natura 2000 shrub-grassland mosaic. Applied Vegetation Science 23(1):39-52 |
| Plant growth form and resprouting capacity for mediterranean species | Silva, V., Catry, F.X, Fernandes, P.M, Rego, F.C., Bugalho, M.N. 2020, Trade-offs between fire hazard reduction and conservation in a Natura 2000 shrub-grassland mosaic. Applied Vegetation Science 23(1):39-52 |
| CMQ lettuce | Simko, I.: Predictive Modeling of a Leaf Conceptual Midpoint Quasi-Color (CMQ) Using an Artificial Neural Network. Sensors 2020, 20, 3938; doi:10.3390/s20143938 |
| Myricaria germanica | Sitzia, T., Michielon, B., Iacopino, S., & Kotze, D. J. (2016). Population dynamics of the endangered shrub Myricaria germanica in a regulated Alpine river is influenced by active channel width and distance to check dams. Ecological engineering, 95, 828-838 |
| Photosynthesis Temperature Response Panama | Slot, M. and Winter, K. (2017), In situ temperature response of photosynthesis of 42 tree and liana species in the canopy of two Panamanian lowland tropical forests with contrasting rainfall regimes. New Phytol, 214: 1103-1117. doi:10.1111/nph.14469 |
| Leaf Respiration Acclimation in Panama | Slot, M., Rey-Sanchez, C., Winter, K. and Kitajima, K. (2014) Trait-based scaling of temperature-dependent foliar respiration in a species-rich tropical forest canopy. Functional Ecology 28: 1074–1086 doi:10.1111/1365-2435.12263 |
| LCE: Leaf carbon exchange dataset for tropical, temperate, and boreal species of North and Central A | Smith, N. G. and Dukes, J. S. (2017), LCE: leaf carbon exchange data set for tropical, temperate, and boreal species of North and Central America. Ecology, 98: 2978. doi:10.1002/ecy.1992 |
| Root Traits of Grassland Species | Smith, S. W., Woodin, S. J., Pakeman, R. J., Johnson, D. and van der Wal, R. (2014), Root traits predict decomposition across a landscape-scale grazing experiment. New Phytologist. Doi: 10.1111/nph.12845 |
| LABDENDRO Brazilian Subtropical Forest Traits Database [Dataset III] | Soboleski,VF, Higuchi, P, Silva, AC, Loebens, R, Souza, K, Buzzi Junior, F, Lima, CL, Cruz, AP, Silva, MAF, & Dallabrida, JP. (2017). Variação de atributos funcionais do componente arbóreo em função de gradientes edáficos em uma floresta nebular no sul do Brasil. Rodriguésia, 68(2), 291-300. https://dx.doi.org/10.1590/2175-7860201768201 |
| La Copita (Texas, USA) woody plant foliar chemical traits | Soper FM, Boutton TW, Sparks JP. 2015. Investigating patterns of symbiotic nitrogen fixation during vegetation change from grassland to woodland using fine scale d15N measurements. Plant, Cell and Environment 38:89–100 |
| Caucasus Plant Traits Database | Soudzilovskaia et al 2013 Functional traits predict relationship between plant abundance dynamic and long-term climate warming, PNAS https://www.pnas.org/content/110/45/18180 |
| Plant traits from alpine plants on Mt. Malaya Khatipara | Soudzilovskaia, NA; Elumeeva, TG; Onipchenko, VG; Shidakov, II; Salpagarova, FS; Khubiev, AB; Tekeev, DK; Cornelissen, JHC. 2013 Functional traits predict relationship between plant abundance dynamic and long-term climate warming. PROCEEDINGS OF THE NATIONAL ACADEMY OF SCIENCES OF THE UNITED STATES OF AMERICA 110(45): 18180-18184 |
| Raw Data João Paulo de Souza | Souza JP, Melo NMJ, Pereira EG, Halfeld AD, Gomes IN, Prado CHBA (2016) Responses of woody Cerrado species to rising atmosphericCO2concentration and water stress: gains and losses. Functional Plant Biology, 43(12): 1183-1193 |
| LABDENDRO Brazilian Subtropical Forest Traits Database [Dataset II] | SOUZA K, HIGUCHI P, SILVA AC, SCHIMALSKI MB, LOEBENS R, BUZZI JUNIOR F, SOUZA CC, RODRIGUES JUNIOR LC, WALTER FF, MISSIO FF, DALLA ROSA A. (2017) Partição de nicho por grupos funcionais de espécies arbóreas em uma floresta subtropical. Rodriguésia [online] vol.68, n.4, pp.1165-1175. ISSN 0370-6583. http://dx.doi.org/10.1590/2175-7860201768401 |
| Niwot Alpine Plant Traits | Spasojevic, M. J. and K. N. Suding. 2012. Inferring community assembly mechanisms from functional diversity patterns: the importance of multiple assembly processes. Journal of Ecology 100:652-661. |
| Ozark Tree leaf traits | Spasojevic, M. J., Turner, B. L., and Myers, J. A. (2016) When does intraspecific trait variation contribute to functional beta‐diversity? J Ecol, 104: 487-496. doi:10.1111/1365-2745.12518 |
| Staples et al Australian Reforestation Tree Database | Staples, TL, Dwyer, JM, England, JR, Mayfield, MM. (2019) Productivity does not correlate with species and functional diversity in Australian reforestation plantings across a wide climate gradient. Global Ecol Biogeogr. 28: 1417– 1429. https://doi.org/10.1111/geb.12962 |
| Plant species high elevation dataset | Steyn, C., Greve, M., Robertson, M. P., Kalwij, J. M. and le Roux, P. C. (2016) Alien plant species that invade high elevations are generalists: support for the directional ecological filtering hypothesis. Journal of Vegetation Science doi:10.1111/jvs.12477 |
| Tropical Plant Traits From Borneo Database | Swaine, E. K. 2007. Ecological and evolutionary drivers of plant community assembly in a Bornean rain forest. PhD Thesis, University of Aberdeen, Aberdeen. |
| CTFS Luquillo Forest Dynamics Plot | Swenson, N.G., J.C. Stegen, S.J. Davies, D.L. Erickson, J. Forero-Montana, A.H. Hurlbert, W.J. Kress, J. Thompson, M. Uriarte, S.J. Wright and J.K. Zimmerman. 2012. Temporal turnover in the composition of tropical tree communities: functional determinism and phylogenetic stochasticity. Ecology 93:490-499 |
| CTFS Luquillo Forest Dynamics Plot | Swenson, N.G., P. Anglada-Cordero, and J.A. Barone. 2011. Deterministic tropical tree community turnover: evidence from patterns of functional beta diversity along an elevational gradient. Proceedings of the Royal Society B 278:877-884. |
| Grassland Plant Trait Database | Takkis, K. 2014. Changes in plant species richness and population performance in response to habitat loss and fragmentation. DISSERTATIONES BIOLOGICAE UNIVERSITATIS TARTUENSIS 255, 2014-04-07. Available from: http://hdl.handle.net/10062/39546 |
| Grassland Plant Trait Database | Takkis, K., Saar, L., Pärtel, M., Helm, A. Effect of environment and landscape on the traits of six plant species in fragmented grasslands. (in preparation) |
| AM fungi and plant growth | Tang X, Daijun Liu, Bo Tu, Xiaohong Yang, Xianhong Yang (2013) Promotion Efect on Growth of Mycorhiza-Inoculated Mulbery Saplings and Physiological and Biochemical Mechanism to Drought Tolerance. J. Southwest University 35, 19-26 |
| A global growth form database for 143,616 vascular plant species | Taseski, G. , Beloe, C. J., Gallagher, R. V., Chan, J. Y., Dalrymple, R. L. and Cornwell, W. K. (2019), A global growth form database for 143,616 vascular plant species. Ecology. doi:10.1002/ecy.2614 |
| BROT 2.0 | Tavsanoglu C. & Pausas J.G. 2018. A functional trait database for Mediterranean Basin plants. Scientific Data 5:180135. http://dx.doi.org/10.1038/sdata.2018.135 |
| Central Balkan Mt dataset | Terziyska, T. S., Tsakalos, J. L., Bartha, S., Apostolova, I., Sopotlieva, D., Zimmermann, Z., Szabo, G. & Wellstein, C. (2019). Species and functional differences between subalpine grasslands with and without dwarf shrub encroachment. Plant Biosystems-An International Journal Dealing with all Aspects of Plant Biology, 1-10. |
| Reliability of leaf functional traits after delayed measurements | Thakur D., Lakhbeer Singh & Amit Chawla (2020) Reliability of leaf functional traits after delayed measurements, Australian Journal of Botany |
| Tree of sex: a database of sexual systems | The Tree of Sex Consortium, Ashman T, Bachtrog D, Blackmon H, Goldberg EE, Hahn MW, Kirkpatrick M, Kitano J, Mank JE, Mayrose I, Ming R, Otto SP, Peichel CL, Pennell MW, Perrin N, Ross L, Valenzuela N, Vamosi JC (2014) Data from: Tree of sex: a database of sexual systems. Dryad Digital Repository. http://dx.doi.org/10.5061/dryad.v1908 |
| Tree of sex: a database of sexual systems | The Tree of Sex Consortium, Ashman T-L, Bachtrog D, Blackmon H, Goldberg EE, Hahn MW, Kirkpatrick M, Kitano J, Mank JE, Mayrose I, Ming R, Otto SP, Peichel CL, Pennell MW, Perrin N, Ross L, Valenzuela N, Vamosi JC (2014) Tree of sex: a database of sexual systems. Scientific Data 1:140015. http://dx.doi.org/10.1038/sdata.2014.15 |
| Traits of Plants in Canada (TOPIC) | Thiffault, N. Canuel, C.-M., Farrell, J.C.G., Hopeting, M. 2012. Conifer regeneration at Green River, New Brunswick. Canadian Forest Service. Unpublished dataset |
| Functional trait data Colombian dry Forest trees | Thomas, E, Carolina Alcazar, Luis Gonzalo Moscoso H., Luis Fernando Osorio, Beatriz Salgado, Mailyn Gonzalez, Mauricio Parra, Michele Bozzano, Judy Loo, Riina Jalonen, Wilson Ramirez (2017) The importance of species selection and seed sourcing in forest restoration for enhancing adaptive potential to climate change: Colombian tropical dry forest as a model, CBD Technical series 89:122-134, https://hdl.handle.net/10568/90681 |
| Wood carbon content database | Thomas, S.C. and A.R. Martin, 2012. Wood carbon content database. Dryad Repository: http://dx.doi.org/10.5061/dryad.69sg2 |
| LECA - Traits of the European Alpine Flora | Thuiller W - Traits of European Alpine Flora - Wilfried Thuiller - OriginAlps Project - Centre National de la Recherche Scientifique |
| Tng et al 2013 Traits | Tng DYP, Jordan GJ, Bowman DMJS (2013) Plant Traits Demonstrate That Temperate and Tropical Giant Eucalypt Forests Are Ecologically Convergent with Rainforest Not Savanna. PLoS ONE 8(12): e84378. https://doi.org/10.1371/journal.pone.0084378 |
| LMA and LDMC of cloud forest tree seedlings in an elevation gradient | Toledo-Aceves, T., García-Hernández, M. & Paz, H. Leaf functional traits predict cloud forest tree seedling survival along an elevation gradient. Annals of Forest Science 76, 111 (2019). https://doi.org/10.1007/s13595-019-0900-5 |
| LBA ECO Tapajos: Leaf Characteristics and Photosynthesis | Tomas F. Domingues, Luiz A. Martinelli, James R. Ehleringer (2007) Ecophysiological traits of plant functional groups in forest and pasture ecosystems from eastern Amazonia, Brazil. Plant Ecol (2007) 193:101–112 DOI 10.1007/s11258-006-9251-z |
| Bay of Biscay dunes | Torca, M., Campos, J.A. Herrera, M. (2019). Species composition and plant traits of south Atlantic European coastal dunes and other comparative data. Data in Brief 22: 207-213 |
| Torres-Ruiz | Torres-Ruiz JM, Cochard H, Fonseca E, Badel E, Gazarini L & Vaz M (2017) Differences in functional and xylem anatomical features allow Cistus species to co-occur and cope differently with drought in the Mediterranean region. Tree Physiology 37: 755–766 Torres-Ruiz JM, Diaz-Espejo A, Morales-Sillero A, Martín-Palomo MJ, Mayr S, Beikircher B & Fernández JE (2013) Shoot hydraulic characteristics, plant water status and stomatal response in olive trees under different soil water conditions. Plant and Soil 373:77-87 Torres-Ruiz JM, Cochard H, Mencuccini M, Delzon S & Badel E (2016) Direct observation and modelling of embolism spread between xylem conduits: a case study in Scots pine. Plant, Cell and Environment 39: 2774-2785 |
| BiolFlor Database | Trefflich, A., Klotz, S. & Kühn, I. (2002): Blühphänologie. In: Klotz, S., Kühn, I. & Durka, W. [eds.]: BIOLFLOR - Eine Datenbank zu biologisch-ökologischen Merkmalen der Gefäßpflanzen in Deutschland. - Schriftenreihe für Vegetationskunde 38: 127-131. Bundesamt für Naturschutz, Bonn. |
| Leaf Traits and Seed Mass of Cover Crops | Tribouillois H, Fort F, Cruz P, Charles R, Flores O, Garnier E, et al. (2015) A Functional Characterisation of a Wide Range of Cover Crop Species: Growth and Nitrogen Acquisition Rates, Leaf Traits and Ecological Strategies. PLoS ONE 10(3): e0122156. doi:10.1371/journal.pone.0122156 |
| Plant Traits for Grassland Species (Konza Prairie, Kansas, USA) | Tucker SS, Craine JM, Nippert JB (2011) Physiological drought tolerance and the structuring of tallgrass assemblages. Ecosphere 2(4): 48 |
| Sheffield Database | unpub. |
| Leaf and Whole Plant Traits Database | unpub. |
| Cedar Creek Plant Physiology Database | unpub. |
| Dispersal Traits Database | unpub. |
| Australian Fire Ecology Database | unpub. |
| Categorical Plant Traits Database | unpub. |
| Overton/Wright New Zealand Database | unpub. |
| Midwestern and Southern US Herbaceous Species Trait Database | unpub. |
| Frost Hardiness Database | unpub. |
| South African Woody Plants Database (ZLTP) | unpub. |
| Tundra Plant Traits Database | unpub. |
| FAPESP Brazil Rainforest Database | unpub. |
| New South Wales Plant Traits Database | unpub. |
| The Netherlands Plant Height Database | unpub. |
| Plant Traits in Pollution Gradients Database | unpub. |
| Photosynthesis Traits Database | unpub. |
| Plant Habit Database | unpub. |
| Leaf and Whole-Plant Traits Database: Hydraulic and Gas Exchange Physiology, Anatomy, Venation Struc | unpub. |
| Leaf Physiology Database | unpub. |
| French Weeds Trait Database | unpub. |
| Traits for Herbaceous Species from Andorra | unpub. |
| Traits for Submerged Species (Aquatic Macrophytes) | unpub. |
| Growth and Herbivory of Juvenil Trees | unpub. |
| Komati Leaf Trait Data | unpub. |
| Cold Tolerance, Seed Size and Height of North American Forest Tree Species | unpub. |
| Meadow Plant Traits: Biomass Allocation, Rooting depth | unpub. |
| Maxfield Meadow, Rocky Mountain Biological Laboratory - LMA | unpub. |
| Baccara - Plant Traits of European Forests | unpub. |
| Plant Functional Traits From the Province of Almeria (Spain) | unpub. |
| Traits for Common Grasses and Herbs in Spain | unpub. |
| Seed Mass from Literature | unpub. |
| Harze Trait Intravar: SLA, LDMC and Plant Height for Calcareous Grassland Species in South Belgium | unpub. |
| Diameter at Breast Height and Life Form of Amazonian Flora | unpub. |
| Functional Traits for Restoration Ecology in the Colombian Amazon | unpub. |
| Catalonian Mediterranean Shrubland Trait Database | unpub. |
| Plant Coastal Dune Traits (France, Aquitaine) | unpub. |
| Crown Architecture Database | unpub. |
| Plant Traits From Spanish Mediteranean shrublands | unpub. |
| Northern mixed-grass prairie species traits - Wyoming, USA | unpub. |
| Leaf traits from the Loess Plateau region of northern Shaanxi in China | unpub. |
| Plant traits from Costa Rica | unpub. |
| Traits of Hypochaeris radicata under shade and drought conditions | unpub. |
| Rocky Mountain Biological Laboratory WSR/gradient plant traits | unpub. |
| Rollinson DBH | unpub. |
| Traits of Ukraine native and invasive plant species | unpub. |
| Plant traits from Wisconsin, USA | unpub. |
| Sphagnum tissue CNP | unpub. |
| Plant Traits from LTER Matsch (Mazia), Italy | unpub. |
| MARGINS - leaf traits database | unpub. |
| Wood traits of trees and lianas from the Brazilian Atlantic Forest | unpub. |
| Trait Data from Niwot Ridge LTER (2016) | unpub. |
| Chinese savanna trees - aboveground trait data | unpub. |
| Iranian Plant Trait Dataset | unpub. |
| TraitDunes | unpub. |
| The Global Leaf Traits | unpub. |
| TRY Categorical Traits Dataset (update 2018) | unpub. |
| Great Basin sagebrush seedlings-greenhouse experiment | unpub. |
| Traits data for plant species from Western Australia | unpub. |
| Leaf traits of beech forest understory species | unpub. |
| Leaf and whole plant traits of Val Cervara old growth beech forest (Central Apennine, Italy) | unpub. |
| Macquarie_xylem_leaf_site_hydraulic | unpub. |
| SLA and height data of exotic plant species in highland forest of Java and Bali | unpub. |
| Leaf traits of selected trees and lianas_vivek | unpub. |
| Traits of Alpine species in GLORIA regions Hochschwab, Schrankogel, Majella and Lefka Ori | unpub. |
| Plants of the Experimental forest of the Botanical Garden Institute FEB RAS (Vladivostok, Russia) | unpub. |
| Leaf functional traits from Sino-US Dimension project (Chinese collaborators) | unpub. |
| LABDENDRO Brazilian Subtropical Forest Traits Database [Dataset IV] | unpub. |
| CLE_restored_prairie_greenhouse_traits | unpub. |
| Charidemi Database | unpub. |
| Sonnier&Boughton_ABS | unpub. |
| Traits_Arum_pictum_Farris_UNISS | unpub. |
| SERC-PREMIS Leaf Trait Dataset | unpub. |
| Yarramundi species trait data | unpub. |
| Soft traits of the Northern Swan Coastal Plain and Geraldton Sandplain kwongan vegetation, Western A | unpub. |
| Watkins, Sjoman and Hitchmough CSR ordination of trees | unpub. |
| inus_traits_Beloiu_Beierkuhnlein_06_2019 | unpub. |
| USA-China Biodiversity (USA samples) | unpub. |
| Alpine tundra plants - effects of climate warming on traits of species in mid-latitude snowbeds | unpub. |
| RASGOS FUNCIONALES ESPECIES ARBOREAS CUENCA AMAZONICA | unpub. |
| Functional Flowering Plant Traits | unpub. |
| Iranian Marl database | unpub. |
| Salguero-Gomez Cistus albidus 2019 | unpub. |
| Golden Gate NP | unpub. |
| Michaletz_CambridgeBay2019 | unpub. |
| Plant functional traits.SLA;Chlorophyll a, b;Carotenoids. Green house at Botanical garden of the Sam | unpub. |
| Functional traits and phylogenetics help explain coastal grassland restoration planting survival and | unpub. |
| Italian Prealps dry grasslands | unpub. |
| Stem hydraulic and anatomical properties of Malagasy Magnoliids | unpub. |
| Leaf, trap, and seed traits of carnivorous plants | unpub. |
| 2018 Talladega National Forest: Foliar Traits | unpub. |
| Functional traits in regenerating Atlantic Forest | unpub. |
| Thom 2019 | unpub. |
| Intraspecific trait variation of dominant grasses in North American Tallgrass Prairie | unpub. |
| ROSALBA MIREYA HERNANDEZ HERRERA | unpub. |
| Coffee plant and leaf trait data in monoculture/agroforestry in CATIE, Costa Rica | unpub. |
| Tundra Tazovsky | unpub. |
| Baccharis pilularis traits growing in Drought Net 2019-20 - Santa Cruz, CA, USA | unpub. |
| Agroforestry tree and leaf trait data from CATIE, Costa Rica | unpub. |
| Pyrenean Alpine Plant Traits | unpub. |
| Independent evolutionary changes in fine-root traits among main clades during the diversification of | Valverde-Barrantes, Oscar; Maherali, Hafiz; Baraloto, Christopher; Blackwood, Christopher (2020), Independent evolutionary changes in fine-root traits among main clades during the diversification of seed plants, v2, Dataset, https://doi.org/10.5061/dryad.2280gb5p9 |
| Forbs and grasses in North East Belgium | Van Cleemput,E., Helsen, K., Feilhauer, H., Honnay, O., Somers, B. (submitted) Spectrally defined plant functional types adequately capture multidimensional trait variation in herbaceous communities |
| Forbs and grasses in North East Belgium | Van Cleemput,E., Roberts, D., Honnay, O., Somers, B. (2019). A novel procedure for measuring functional traits of herbaceous species through field spectroscopy. Methods in Ecology and Evolution. XXX(XXX). https://doi.org/10.1111/2041-210X.13237 |
| LMA, leaf tissue density and N&P content along the Amazon-Andes gradient in Peru | van de Weg MJ, Meir P,Grace J, Atkin O (2009) Altitudinal variation in leaf mass per unit area, leaf tissue density and foliar nitrogen and phosphorus content along the Amazon-Andes gradient in Peru, Plant Ecology& Diversity, 2: 3, 243-254 |
| Photosynthetic parameters, respiration and leaf traits of a Peruvian tropical montane cloud forest | van de Weg MJ, Patrick Meir John Grace, Guilmair Damian Ramos (2011) Photosynthetic parameters, dark respiration and leaf traits in the canopy of a Peruvian tropical montane cloud forest Oecologia DOI 10.1007/s00442-011-2068-z |
| Montane_grassland_FT | van der Merwe S., Greve, M., Olivier, B. & le Roux, P.C. 2019. The role of facilitation in functional trait expression: testing the generality in two contrasting systems. in prep |
| sub-Antarctic_tundra_FT | van der Merwe S., Greve, M., Olivier, B. & le Roux, P.C. 2019. The role of facilitation in functional trait expression: testing the generality in two contrasting systems. in prep |
| Traits of savannah trees in the Hluhluwe-iMfolozi Game reserve, South Africa | van der Plas, F. & Olff, H. (2014) Mesoherbivores affect grasshopper communities in a megaherbivore-dominated South African savannah. Oecologia 175: 639. doi:10.1007/s00442-014-2920-z |
| Traits of savannah trees in the Hluhluwe-iMfolozi Game reserve, South Africa | Van der Plas, F., Howison, R., Reinders, J., Fokkema, W. and Olff, H. (2013), Functional traits of trees on and off termite mounds: understanding the origin of biotically-driven heterogeneity in savannas. J Veg Sci, 24: 227–238. doi:10.1111/j.1654-1103.2012.01459.x |
| Trait data Pibiri - Masha van der Sande | van der Sande, M.T., Arets, E.J.M.M., Peña-Claros, M., Hoosbeek, M.R., Cáceres-Siani, Y., van der Hout, P. & Poorter, L. (2018) Soil fertility and species traits, but not diversity, drive productivity and biomass stocks in a Guyanese tropical rainforest. Functional Ecology, 32, 461–474 |
| Mean leaf trait values of woody seedlings of a Caatinga Dry Forest | Vanderlei, R. S., Barros, M. F., Leal, I. R., & Tabarelli, M. (2022). Impoverished woody seedling assemblages and the regeneration of Caatinga dry forest in a human-modified landscape. Biotropica, 54, 670– 681. https://doi.org/10.1111/btp.13081 |
| Western Pamirs | Vanselow, K.A., Samimi, C., Breckle, S.-W. (2016): Preserving a Comprehensive Vegetation Knowledge Base – An Evaluation of Four Historical Soviet Vegetation Maps of the Western Pamirs (Tajikistan). PLoS ONE 11(2): e0148930. doi:10.1371/journal.pone.0148930 |
| functional traits native and invasive species in tropical dry forest | Vásquez-Valderrama, M. (2016).Efecto de especies con potencial invasor en procesos de regulación hídrica del suelo en un ecosistema seco tropical. Maestría en Manejo, Uso y Conservación del Bosque, Universidad Distrital Francisco José de Caldas, Facultad del Medio Ambiente y Recursos Naturales. Bogotá, Colombia |
| Functional Traits Of Bulgarian Grasslands | Vassilev, K., Pedashenko, H., Nikolov, S., Apostolova, I., Dengler, J. 2011. The effect of land abandonment on the vegetation of upland semi-natural grasslands in the Western Balkans MTS, Bulgaria. - Plant Biosystems, 145: 654-665. |
| Delpierre_QuercusPetraea_Photosynthesis_Seasonal_ClimateChambers | Verdier, B., Jouanneau, I., Simonnet, B., Rabin, C., Van Dooren, T. J., Delpierre, N., ... & Le Galliard, J. F. (2014). Climate and atmosphere simulator for experiments on ecological systems in changing environments. Environmental science & technology, 48(15), 8744-8753. Delpierre et al. (unpublished) |
| Nutrient Resorption Efficiency Database | Vergutz, L., Manzoni, S., Porporato, A., Novais, R. F., and Jackson, R. B. 2012. Global resorption efficiencies and concentrations of carbon and nutrients in leaves of terrestrial plants, Ecological Monographs 82:2, 205-220. doi: 10.1890/11-0416.1 |
| Nutrient Resorption Efficiency Database | Vergutz, L., S. Manzoni, A. Porporato, R.F. Novais, and R.B. Jackson. 2012. A Global Database of Carbon and Nutrient Concentrations of Green and Senesced Leaves. Data set. Available on-line [http://daac.ornl.gov] from Oak Ridge National Laboratory Distributed Active Archive Center, Oak Ridge, Tennessee, U.S.A. http://dx.doi.org/10.3334/ORNLDAAC/1106 |
| Leaf and Whole Plant Traits Database | Vile, D. 2005. Significations fonctionnelle et ecologique des traits des especes vegetales: exemple dans une succession post-cultural mediterraneenne et generalisations, PHD Thesis. |
| Plant Traits, Virginia, USA | Von Holle, B. and D. Simberloff. 2004. Testing Fox's assembly rule: Does plant invasion depend on recipient community structure? Oikos 105:551-563. |
| Wagenführ Woodatlas | Wagenführ R. (2007) Holzatlas. 6., neu bearbeitete und erweiterte Auflage. Fachbuchverlag Leipzig |
| Leaf and Whole-Plant Traits Database: Hydraulic and Gas Exchange Physiology, Anatomy, Venation Struc | Waite, M. and L. Sack. 2010. How does moss photosynthesis relate to leaf and canopy structure? Trait relationships for 10 Hawaiian species of contrasting light habitats. New Phytologist 185:156-172. |
| A Global Data Set of Leaf Photosynthetic Rates, Leaf N and P, and Specific Leaf Area | Walker AP, AP Beckerman, L Gu , J Kattge, LA Cernusak, TF Domingues, JC Scales, G Wohlfahrt, SD Wullschleger and FI Woodward (2014) The relationship of leaf photosynthetic traits – Vcmax and Jmax – to leaf nitrogen, leaf phosphorus, and specific leaf area: a meta-analysis and modeling study. Ecology and Evolution doi: 10.1002/ece3.1173 |
| A Global Data Set of Leaf Photosynthetic Rates, Leaf N and P, and Specific Leaf Area | Walker, A.P. 2014. A Global Data Set of Leaf Photosynthetic Rates, Leaf N and P, and Specific Leaf Area. Data set. Available on-line [http://daac.ornl.gov] from Oak Ridge National Laboratory Distributed Active Archive Center, Oak Ridge, Tennessee, USA. http://dx.doi.org/10.3334/ORNLDAAC/1224 |
| Weiqi-Sardans-Penuelas-China-plants | Wang W, Min Q, Sardans J, Wang C, Asensio D, Bartrons M, Peñuelas J. Organic cultivation of jasmine and tea increases carbon sequestration by changing plant and soil stoichiometry. Agronomy Journal, 2016, 108: 1-13. |
| Weiqi-Sardans-Penuelas-China-plants | Wang W, Sardans J, Tong C, Wang C, Ouyang L, Bartrons M, Peñuelas J. Typhoon enhancement of N and P release from litter and changes in the litter N:P ratio in a subtropical tidal wetland.Environmental Research Letter, 2016, 11 :014003 |
| Weiqi-Sardans-Penuelas-China-plants | Wang W, Sardans J, Wang C, Pan T, Zeng C, Lai D Y F, Bartrons M, Peñuelas J. Straw Application strategy to optimize nutrient release in a southeastern China rice cropland. Agronomy, 2017, 7: 84 |
| Weiqi-Sardans-Penuelas-China-plants | Wang W, Sardans J, Wang C, Zeng C, Tong C, Asensio D, Peñuelas J. Ecological stoichiometry of C, N, and P of invasive Phragmites australis and native Cyperus malaccensis species in the Minjiang River tidal estuarine wetlands of China. Plant Ecology, 2015, 216: 809-822. |
| Weiqi-Sardans-Penuelas-China-plants | Wang W, Sardans J, Wang C, Zeng C, Tong C, Bartrons M, Peñuelas J. Steel slag amendment increases nutrient availability and rice yield in a subtropical paddy field in china. Experimental Agriculture, 2018, 54: 842-856 |
| Weiqi-Sardans-Penuelas-China-plants | Wang W, Wang C, Sardans J, Tong C, Ouyang L, Asensio D, Gargallo-Garriga A, Peñuelas J. Storage and release of nutrients during litter decomposition for native and invasive species under different flooding intensities in a Chinese wetland. Aquatic Botany, 2018, 149: 5-16 |
| Weiqi-Sardans-Penuelas-China-plants | Wang W, Zeng C, Sardans J, Wang C, Tong C, Peñuelas J. Soil methane production, anaerobic and aerobic oxidation in porewater of wetland soils in the Minjiang River estuarine, China. Wetlands, 2018, 38: 627-640. |
| Weiqi-Sardans-Penuelas-China-plants | Wang W, Zeng C, Sardans J, Zeng D, Wang C, Bartrons M, Peñuelas J. Industrial and agricultural wastes decreased greenhouse-gas emissions and increased rice grain yield in a subtropical paddy field. Experimental Agriculture, 2018, 54: 623–640 |
| The China Plant Trait Database | Wang, Han; Harrison, Sandy P; Prentice, Iain Colin; Yang, Yanzheng; Bai, Fan; Furstenau Togashi, Henrique; Wang, Meng; Zhou, Shuangxi; Ni, Jian (2017): The China Plant Trait Database. PANGAEA, https://doi.org/10.1594/PANGAEA.871819 |
| Weiqi-Sardans-Penuelas-China-plants | Wang, W. Q., Wang, C., Sardans, J., Zeng, C. S., Tong, C., & Peñuelas, J. (2015). Plant invasive success associated with higher n-use efficiency and stoichiometric shifts in the soil–plant system in the minjiang river tidal estuarine wetlands of china. Wetlands Ecology & Management,23(5), 865-880. |
| Traits of Plants in Canada (TOPIC) | Wei, L. and Fenton, N.J. 2016. stem specific density and specific leaf area measured for project "Silviculture based on plant functional traits in boreal forests". Université de Québec en Abitibi-Témiscamingue. Unpublished data |
| Whole plant traits and leaf traits of four grassland species in Central Apennines (Italy) | Wellstein C., Chelli S., Campetella G., Bartha S., Galiè M., Spada F., Canullo R. (2013): Intraspecific phenotypic variability of plant functional traits in contrasting mountain grasslands habitats. Biodiversity and Conservation, 22(10), 2353-2374 |
| Leaf Ash Content in China's Terrestrial Plants | Wenxuan Han, Yahan Chen, Fang-Jie Zhao, Luying Tang, Rongfeng Jiang and Fusuo Zhang, 2012, Floral, climatic and soil pH controls on leaf ash content in China’s terrestrial plants. Global Ecology and Biogeography, DOI: 10.1111/j.1466-8238.2011.00677.x |
| Mycorrhizal Association Database | Werner, G. D. A., Cornelissen, J. H. C., Cornwell, W. K., Soudzilovskaia, N. A., Kattge, J., West, S. A., Kiers, E. T. (2018) Symbiont switching and alternative resource acquisition strategies drive mutualism breakdown. Proceedings of the National Academy of Sciences (PNAS) 201721629; DOI: 10.1073/pnas.1721629115 |
| BIOME-BGC Parameterization Database | White, M. A., P. E. Thornton, S. W. Running, and R. R. Nemani. 2000. Parameterization and sensitivity analysis of the BIOME-BGC terrestrial ecosystem model: Net primary production controls. Earth Interactions 4:1-85. |
| Lariz Occidentalis branch section specific leaf area and dry mass | Williams, G.M. and Nelson, A.S. 2018. Spatial variation in specific leaf area and horizontal distribution of leaf area in juvenile western larch (Larix occidentalis Nutt.). Trees – Structure and Function. 32(6): 1621-1631. DOI: 10.1007/s00468-018-1738-4 |
| LBA-ECO CD-09 Soil and Vegetation Characteristics, Tapajos National Forest, Brazil | Williams, M., Y.E. Shimabokuro and E.B. Rastetter. 2012. LBA-ECO CD-09 Soil and Vegetation Characteristics, Tapajos National Forest, Brazil. Data set. Available on-line [http://daac.ornl.gov] from Oak Ridge National Laboratory Distributed Active Archive Center, Oak Ridge, Tennessee, U.S.A. http://dx.doi.org/10.3334/ORNLDAAC/1104 |
| Leaf Photosynthesis and Nitrogen at Oak Ridge Dataset | Wilson K., D. Baldocchi, P. Hanson (2000) Spatial and seasonal variability of photosynthetic parameters and their relationship to leaf nitrogen in a deciduous forest. Tree Physiology 20, 565–578 |
| The Functional Ecology of Trees (FET) Database - Jena | Wirth, C. and J. W. Lichstein. 2009. The Imprint of Species Turnover on Old-Growth Forest Carbon Balances - Insights From a Trait-Based Model of Forest Dynamics. Pages 81-113 in C. Wirth, G. Gleixner, and M. Heimann, editors. Old-Growth Forests: Function, Fate and Value. Springer, New York, Berlin, Heidelberg. |
| European Mountain Meadows Plant Traits Database | Wohlfahrt, G., M. Bahn, E. Haubner, I. Horak, W. Michaeler, K. Rottmar, U. Tappeiner, and A. Cernusca. 1999. Inter-specific variation of the biochemical limitation to photosynthesis and related leaf traits of 30 species from mountain grassland ecosystems under different land use. Plant, Cell and Environment 22:1281-1296. |
| IanWrightNTsavannaTraits | Wright IJ, CER Lehmann & AE Richards, unpublished |
| IanWrightNTsavannaTraits | Wright IJ, Cooke J, Cernusak LA, Hutley LB, Scalon MC, Tozer WC, Lehmann CER (2019). Stem diameter growth rates in a fire-prone savanna correlate with photosynthetic rate and branch-scale biomass allocation, but not specific leaf area. Austral Ecol 44:339-350 |
| Leaf Economic Traits Across Varying Environmental Conditions | Wright JP, Sutton-Grier A (2012) Does the leaf economic spectrum hold within local species pools across varying environmental conditions? Functional Ecology 2012 doi: 10.1111/1365-2435.12001 |
| Neotropical Plant Traits Database | Wright, I. J., D. D. Ackerly, F. Bongers, K. E. Harms, G. Ibarra-Manriquez, M. Martinez-Ramos, S. J. Mazer, H. C. Muller-Landau, H. Paz, N. C. A. Pitman, L. Poorter, M. R. Silman, C. F. Vriesendorp, C. O. Webb, M. Westoby, and S. J. Wright. 2007. Relationships among ecologically important dimensions of plant trait variation in seven Neotropical forests. Annals of Botany 99:1003-1015. |
| Global leaf size dataset | Wright, I. J., N. Dong, V. Maire, I. C. Prentice, M. Westoby, S. Díaz, R. V. Gallagher, B. F. Jacobs, R. Kooyman, E. A. Law, M. R. Leishman, Ü. Niinemets, P. B. Reich, L. Sack, R. Villar, H. Wang and P. Wilf (2017). Global climatic drivers of leaf size. Science 357(6354): 917-921. DOI:10.1126/science.aal4760 |
| GLOPNET - Global Plant Trait Network Database | Wright, I. J., P. B. Reich, M. Westoby, D. D. Ackerly, Z. Baruch, F. Bongers, J. Cavender-Bares, T. Chapin, J. H. C. Cornelissen, M. Diemer, J. Flexas, E. Garnier, P. K. Groom, J. Gulias, K. Hikosaka, B. B. Lamont, T. Lee, W. Lee, C. Lusk, J. J. Midgley, M. L. Navas, U. Niinemets, J. Oleksyn, N. Osada, H. Poorter, P. Poot, L. Prior, V. I. Pyankov, C. Roumet, S. C. Thomas, M. G. Tjoelker, E. J. Veneklaas, and R. Villar. 2004. The worldwide leaf economics spectrum. Nature 428:821-827. |
| GLOPNET - Global Plant Trait Network Database | Wright, I. J., P. B. Reich, O. K. Atkin, C. H. Lusk, M. G. Tjoelker, and M. Westoby. 2006. Irradiance, temperature and rainfall influence leaf dark respiration in woody plants: evidence from comparisons across 20 sites. New Phytologist 169:309-319. |
| Panama Plant Traits Database | Wright, S. J., K. Kitajima, N. J. B. Kraft, P. B. Reich, I. J. Wright, D. E. Bunker, R. Condit, J. W. Dalling, S. J. Davies, S. Díaz, B. M. J. Engelbrecht, K. E. Harms, S. P. Hubbell, C. O. Marks, M. C. Ruiz-Jaen, C. M. Salvador, and A. E. Zanne. 2011 . Functional traits and the growth-mortality tradeoff in tropical trees. Ecology 91:3664-3674. |
| Plant three traits (SLA,LA, Height) of 14 plots in Easten Tibetan subalpine meadow | Xin-e Li; Yingying Nie; Xiaoyu Song; Renyi Zhang and Gang Wang*.Patterns of species diversity and functional diversity along a south-to north-facing slope in a sub-alpine meadow. Community Ecology, 2011, 12（2）：179-187 |
| Photosynthesis Traits Database | Xu, L. K. and D. D. Baldocchi. 2003. Seasonal trends in photosynthetic parameters and stomatal conductance of blue oak (Quercus douglasii) under prolonged summer drought and high temperature. Tree Physiology 23:865-877. |
| Leaf Nitrogen and Phosphorus for China's Terrestrial Plants | Yahan Chen , Wenxuan Han , Luying Tang , Zhiyao Tang and Jingyun Fang 2011 Leaf nitrogen and phosphorus concentrations of woody plants differ in responses to climate, soil and plant growth form. Ecography 34, doi: 10.1111/j.1600-0587.2011.06833.x |
| Shoot N/P stoichiometry of Inner Mongolia grassland species | Yu Q., Elser J.J., He N., Wu H., Chen Q., Zhang G., Han X. (2011) Stoichiometric homeostasis of vascular plants in the Inner Mongolia grassland. Oecologia 166:1 doi:10.1007/s00442-010-1902-z |
| Global Leaf Phenology Database | Zanne, A.E., Cornwell, W.K., McGlinn, D.J., Beaulieu, J.M., Eastman, J.M., FitzJohn, R.G., Smith, S.A., Aarssen, L., Bertin, R.I., Calaminus, A., Govaerts, R., Hemmings, F., Leishman, M.R., Moles, A.T., Oleksyn, J., Ordonez, A., Reich, P.B., Royer, D.L., Soltis, D.E., Soltis, P.S., Stevens, P.F., Swenson, N.G., Warman, L., Wright, I.J., Tank, D.C. (2013) Data from: Three keys to the radiation of angiosperms into freezing environments. Dryad Digital Repository http://dx.doi.org/10.5061/dryad.63q27/1 |
| Global Leaf Phenology Database | Zanne, A.E., Tank, D.C., Cornwell, W.K., Eastman, J.M., Smith, S.A., FitzJohn, R.G., McGlinn, D.J., Moles, A.T., O'Meara, B.C., Royer, D.L., Wright, I.J., Aarssen, L., Bertin, R.I., Govaerts, R., Hemmings, F., Leishman, M.R., Oleksyn, J., Reich, P.B., Sargent, R., Soltis, D.E., Soltis, P.S., Stevens, P.F., Swenson, N.G., Warman, L., Westoby, M., Beaulieu, J.M. Three keys to the radiation of angiosperms into freezing environments. (2013) Nature. doi:10.1038/nature12872 |
| Global Woodiness Database | Zanne, A.E., Tank, D.C., Cornwell, W.K., Eastman, J.M., Smith, S.A., FitzJohn, R.G., McGlinn, D.J., Moles, A.T., O'Meara, B.C., Royer, D.L., Wright, I.J., Aarssen, L., Bertin, R.I., Govaerts, R., Hemmings, F., Leishman, M.R., Oleksyn, J., Reich, P.B., Sargent, R., Soltis, D.E., Soltis, P.S., Stevens, P.F., Swenson, N.G., Warman, L., Westoby, M., Beaulieu, J.M. Three keys to the radiation of angiosperms into freezing environments. (2013) Nature. doi:10.1038/nature12872 |
| Minimum Freezing Exposure Database | Zanne, A.E., Tank, D.C., Cornwell, W.K., Eastman, J.M., Smith, S.A., FitzJohn, R.G., McGlinn, D.J., Moles, A.T., O'Meara, B.C., Royer, D.L., Wright, I.J., Aarssen, L., Bertin, R.I., Govaerts, R., Hemmings, F., Leishman, M.R., Oleksyn, J., Reich, P.B., Sargent, R., Soltis, D.E., Soltis, P.S., Stevens, P.F., Swenson, N.G., Warman, L., Westoby, M., Beaulieu, J.M. Three keys to the radiation of angiosperms into freezing environments. (2013) Nature. doi:10.1038/nature12872 |
| TRY_Metasequoia glyptostroboides_Shanghai_China | Zheng, J., Blanco, J. A., Wei, X., & Liu, C. (2018). Sustainable management of metasequoia glyptostroboides plantation forests in shanghai. Forests, 9(2), 64 |
| TRY_Metasequoia glyptostroboides_Shanghai_China | Zheng, J., Zang, H., Yin, S., Sun, N., Zhu, P., Han, Y., Kang, H., Liu, C. (2018). Modeling height-diameter relationship for artificial monoculture Metasequoia glyptostroboides in sub-tropic coastal megacity Shanghai, China. Urban Forestry & Urban Greening, 34, 226-232 |
| Maximum Height of Chinese Tree Species (From Silva Sinica) | Zheng, W. 1983. Silva Sinica: Volume 1-4. China Forestry Publishing House, Beijing. |
| Wood anatomy and wood density - Australia | Ziemińska, K., Butler, D. W., Gleason, S. M., Wright, I. J. & Westoby, M. Fibre wall and lumen fractions drive wood density variation across 24 Australian angiosperms. AoB PLANTS 5, (2013). |
| Wood anatomy and wood density - Australia | Ziemińska, K., Westoby, M. & Wright, I. J. Broad Anatomical Variation within a Narrow Wood Density Range—A Study of Twig Wood across 69 Australian Angiosperms. PLoS ONE 10, e0124892 (2015). |
| SW Michigan restored prairies | Zirbel CR, Bassett T, Grman E, Brudvig LA (2017) Plant functional traits and environmental conditions shape community assembly and ecosystem functioning during restoration. Journal of Applied Ecology 54(4): 1070-1079. https://doi.org/10.1111/1365-2664.12885 Zirbel CR, Basset T, Grman E, Brudvig LA (2017) Data from: Plant functional traits and environmental conditions shape community assembly and ecosystem functioning during restoration. Dryad Digital Repository. https://doi.org/10.5061/dryad.2175q |
